# EnrichRBP: an automated and interpretable computational platform for predicting and analysing RNA-binding protein events

**DOI:** 10.1093/bioinformatics/btaf018

**Published:** 2025-01-13

**Authors:** Yubo Wang, Haoran Zhu, Yansong Wang, Yuning Yang, Yujian Huang, Jian Zhang, Ka-chun Wong, Xiangtao Li

**Affiliations:** School of Artificial Intelligence, Jilin University, Changchun 130012, China; School of Artificial Intelligence, Jilin University, Changchun 130012, China; School of Artificial Intelligence, Jilin University, Changchun 130012, China; Information Science and Technology, Northeast Normal University, Changchun 130024, China; College of Computer Science and Cyber Security, Chengdu University of Technology, Chengdu 610059, China; School of Computer and Information Technology, Xinyang Normal University, Xinyang 464000, China; Department of Computer Science, City University of Hong Kong, Hong Kong SAR 999077, China; School of Artificial Intelligence, Jilin University, Changchun 130012, China

## Abstract

**Motivation:**

Predicting RNA-binding proteins (RBPs) is central to understanding post-transcriptional regulatory mechanisms. Here, we introduce EnrichRBP, an automated and interpretable computational platform specifically designed for the comprehensive analysis of RBP interactions with RNA.

**Results:**

EnrichRBP is a web service that enables researchers to develop original deep learning and machine learning architectures to explore the complex dynamics of RBPs. The platform supports 70 deep learning algorithms, covering feature representation, selection, model training, comparison, optimization, and evaluation, all integrated within an automated pipeline. EnrichRBP is adept at providing comprehensive visualizations, enhancing model interpretability, and facilitating the discovery of functionally significant sequence regions crucial for RBP interactions. In addition, EnrichRBP supports base-level functional annotation tasks, offering explanations and graphical visualizations that confirm the reliability of the predicted RNA-binding sites. Leveraging high-performance computing, EnrichRBP provides ultra-fast predictions ranging from seconds to hours, applicable to both pre-trained and custom model scenarios, thus proving its utility in real-world applications. Case studies highlight that EnrichRBP provides robust and interpretable predictions, demonstrating the power of deep learning in the functional analysis of RBP interactions. Finally, EnrichRBP aims to enhance the reproducibility of computational method analyses for RBP sequences, as well as reduce the programming and hardware requirements for biologists, thereby offering meaningful functional insights.

**Availability and implementation:**

EnrichRBP is available at https://airbp.aibio-lab.com/. The source code is available at https://github.com/wangyb97/EnrichRBP, and detailed online documentation can be found at https://enrichrbp.readthedocs.io/en/latest/.

## 1 Introduction

RNA-binding proteins (RBPs) constitute a diverse class of biomolecules that are decisive in many cellular functions. By interacting with RNA, they facilitate a spectrum of key activities such as, functional execution, regulation of RNA synthesis and degradation, and control of protein–protein interactions (Gerstberger *et al.* 2014, Hentze *et al.* 2018). This interaction makes elucidating the RNA–RBP binding mechanisms essential for a deeper understanding of biological processes. To date, various high-throughput methods, such as UV cross-linking and immunoprecipitation (CLIP-seq) (Hafner *et al.* 2010), have been developed to identify RNA-binding sites and assess the affinity of RBPs. Moreover, advanced variants of CLIP-seq, such as individual-nucleotide resolution CLIP (iCLIP) (König *et al.* 2010) and enhanced CLIP (eCLIP) (Van Nostrand *et al.* 2016), enable the detection of protein–RNA cross-linking events at single-nucleotide resolution. In addition to the mainstream CLIP technologies mentioned above, many derivative CLIP techniques have also been developed. For instance, Sugimoto *et al.* (2017) introduced an RNA hybridization and single-nucleotide resolution CLIP technique (hiCLIP) to identify RNA double strands bound by RBPs. Since the above methods mainly focus on the stable binding state of RNA–RBP interaction at a specific timepoint, they are unable to capture the dynamic mechanistic changes of intermolecular binding. To address this limitation, Cordiner *et al.* (2023) developed a multi-timepoint iCLIP technique (temporal-iCLIP, tiCLIP), which allows for the study of the temporal nature within RNA–RBP binding. However, these techniques still rely on traditional, labor-intensive experimental approaches, which pose substantial challenges. To overcome those problems, the rapid development of computational models has opened new avenues for understanding the structural and functional mechanisms of RBPs.

In recent years, computational web servers have been created to overcome limitations in biological sequence analysis. These servers are categorized into two main types. The first category focuses on generalized prediction, employing a wide range of algorithms to comprehensively predict RBP binding sites. For instance, catRAPID omics (Agostini *et al.* 2013) is a server designed to predict protein–RNA interactions at proteomic and transcriptomic levels. catRAPID omics v2.0 (Armaos *et al.* 2021) extends these capabilities to analyse protein–RNA interaction propensities across eight model organisms, demonstrating the broad utility of the tool. RNAincoder (Wang *et al.* 2023) offers a comprehensive suite of RNA-encoded features and employs a machine learning or deep learning-based strategy for representing RNA-related interactions. Although these platforms include RNA–RBP binding prediction modules, they often fall short in providing comprehensive biological insight, with limited model interpretability and accuracy. From the perspective of interpretability, catRAPID omics and catRAPID omics v2.0 fail to incorporate motif discovery, restricting their capacity to elucidate the intermolecular binding mechanisms. RNAincoder also demonstrates limited model interpretability, as minimal insight is provided into the sequence or structural basis of RNA–RBP interactions. In terms of accuracy, catRAPID omics and catRAPID omics v2.0 rely on generic RBP predictive models, leading to inconsistent performance across specific tasks. RNAincoder, on the other hand, adopts a relatively simple machine learning or deep learning framework, which makes it difficult to capture the complex contextual interaction mechanism of RNA–RBP. Detailed comparisons of the accuracy of these platforms are presented in Section 3.1.

The second type of web server consists of dedicated servers equipped with specialized algorithms to perform specific computational tasks, especially identifying particular classes of RNA–RBP binding sites. For instance, RBPmap (Paz *et al.* 2014) provides a platform for predicting and mapping RBP binding sites across RNA sequences, serving as a valuable resource for researchers. RBPsuite (Pan *et al.* 2020) integrates two deep learning-based methods, iDeepS (Pan *et al.* 2018) and CRIP (Zhang *et al.* 2019), for the prediction of binding preference of linear RNA and circular RNA, respectively. PrismNet (Xu *et al.* 2023) enhances this framework by integrating RNA secondary structure (icSHAPE) (Spitale *et al.* 2015) with sequential data to predict cell type-specific RNA–RBP interactions. The input of two types of existing web servers typically requires more than just the basic RNA sequence; additional information such as RBP sequences, RNA secondary structure, or specific motif details is often necessary to improve prediction accuracy. As for the output, these servers usually provide the probability of RBP binding, motif analysis, or model interpretability. However, each server is usually not comprehensive in terms of result analysis. Therefore, there is an urgent need for an integrated and intuitive platform, enabling researchers to efficiently and accurately identify binding sites in various RNAs and RBPs across diverse environments while providing robust and comprehensive result analyses.

In this study, we introduce EnrichRBP, a novel computational platform that employs automated machine learning and deep learning techniques for RNA sequence prediction. EnrichRBP offers advanced result visualization and biological interpretability. The platform distinguishes itself with several unique attributes: (i) EnrichRBP features two major prediction modules that include nine state-of-the-art methods for rapid RNA–RBP binding prediction and 61 customizable approaches for RNA–RBP binding prediction. Moreover, these modules include 28 sequence characterization techniques, 18 feature selection methods, and 15 prediction algorithms. The platform also performs a comprehensive comparative analysis of model predictions against experimental data to verify the reliability of the predictions. (ii) Extensive visualization and analysis capabilities are embedded within EnrichRBP, offering 27 interactive visual analyses across five domains: dataset statistical analysis, training process analysis, result visualization, feature analysis, and biological interpretability. Overall, EnrichRBP represents a comprehensive workflow for the reliable and interpretable analysis of RBP interactions.

## 2 Materials and methods

### 2.1 The overall framework of EnrichRBP

**Figure 1.**
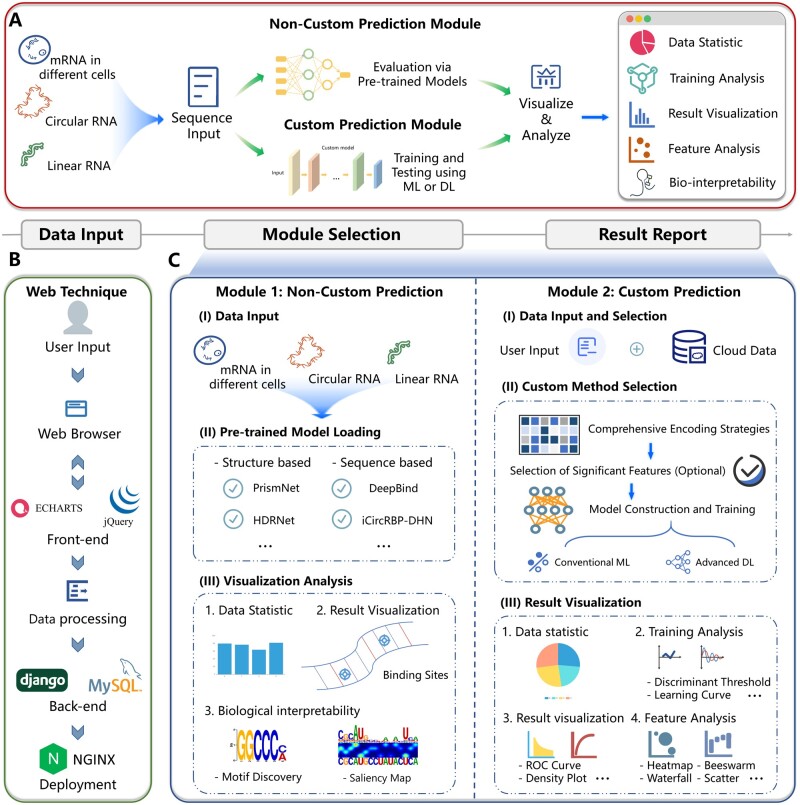
Overview of the EnrichRBP platform. (A) EnrichRBP contains four modules: the data input module, the non-custom prediction module, the custom prediction module, and the result reporting module. (B) The web technique of EnrichRBP. (C) The workflow of the two functional modules. The non-custom prediction module has three stages: (i) data input; (ii) pre-trained model loading; and (iii) visualization analysis. The custom prediction module has the following stages: (i) data input and selection; (ii) custom method selection; and (iii) results visualization.

The EnrichRBP platform integrates a comprehensive, automated pipeline leveraging 70 state-of-the-art (SOTA) advanced deep learning and traditional machine learning methodologies to predict RBP sites across a wide range of RNA sequences inputted by users. Figure 1A conceptualizes EnrichRBP’s overall framework, split into four primary modules: (i) data input, (ii) non-custom prediction, (iii) custom prediction, and (iv) result reporting. The workflow of EnrichRBP is as follows. In the beginning, EnrichRBP receives RNA sequence data from the data input module, where it can handle transcriptomic sequence types: circular RNA (circRNA), linear RNA, and RNA in various cellular or tissue contexts. The process is depicted in Fig. 1B, which provides a granular view of the web architecture and delineates the interaction dynamics between the frontend user interface and the backend processing unit. Furthermore, the EnrichRBP platform contains two distinct functional modules tailored to specific tasks: The first is a non-custom prediction module that utilizes existing computational models for rapid binding site identification. The second is a custom prediction module, enabling the generation of potential feature maps from biological representations considering dynamic and static semantic information, physicochemical properties, and RNA structure. This module efficiently reduces the complexity of the high-dimensional feature space by selecting the most relevant features, which precedes the autonomous training and prediction.

The non-custom prediction module employs computational models for the rapid identification of CircRNA-RBPs, Linear RNA-RBPs, and RNA-RBP in cells, encompassing three key stages depicted in Fig. 1C: (i) data input; (ii) existing computational model loading; and (iii) visualization analysis. Additionally, the custom prediction module facilitates the automatic training, evaluation, and comparison of machine learning and deep learning models based on user-provided data, structured into three phases shown in Fig. 1C: (i) data input and selection; (ii) custom method selection; and (iii) results visualization. The results reporting module offers a suite of visualization tools for analysis, with results readily available in a one-click download, enhancing accessibility for researchers with limited programming skills by eliminating computational infrastructure challenges. Subsequent sections will detail these four modules.

### 2.2 Benchmark datasets

We collected four benchmark datasets for training and prediction in the platform, including 37 circRNA-RBP binding site fragment datasets, 31 linear RNA-RBP binding site datasets, a comprehensive dataset of 740 full-length circRNA-RBP binding sites, and 261 datasets of mRNA from various cell lines from multiple databases. Specifically, we used a dataset comprising 32 216 circRNA sequences with associated binding proteins from the circRNA interactome database (Dudekula *et al.* 2016). To enhance quality, we employed CD-HIT-EST software (Li and Godzik 2006) to remove redundancies and merge positive and negative circRNA fragments. Positive samples comprised 101-nucleotide sequences adjacent to each CLIP-seq peak, while negative samples were randomly selected from non-overlapping fragments. Moreover, we also incorporated 31 datasets of linear RNA-RBP binding sites, encompassing 19 RBPs involved in splicing (e.g. hnRNPs, U2AF2, ELAVL1, TDP-43, FUS) and 3′ UTR processing (e.g. Ago, IGF2BP), sourced from the iONMF database. Each dataset contains 6000 RBP binding sites, with positive and negative samples generated from the literature (Stražar *et al.* 2016). Furthermore, we analysed 261 RBP binding site datasets from multiple databases, focusing on six cell lines: K562, HepG2, HEK293, HEK293T, HeLa, and H9. In these datasets, 172 RBPs were identified using a uniform flag-marking technique. We included 65 CLIP-seq datasets for 61 RBPs from the POSTAR database (Zhu *et al.* 2019) and 196 eCLIP datasets for 111 RBPs from the ENCODE project (Van Nostrand *et al.* 2020). For a detailed overview of the databases used in EnrichRBP, please refer to Supplementary Table S17.

### 2.3 Data input module

In the initial module, EnrichRBP is designed to process input sequences of three specific RNA categories: circRNA, linear RNA, and RNA from diverse cellular environments. Users can upload a single FASTA file containing the RNA sequences to the webserver. In addition, users can select the model and specific RBP types (or an unknown RBP type). EnrichRBP subsequently conducts data integrity and availability checks to confirm the validity of the sequences before processing them in the subsequent modules. Initially, the platform assesses the conformity of the input sequence data to the required FASTA format. Subsequently, we verify the absence of illicit characters, ensuring that the input sequences comprise only permissible characters, namely A, G, C, and U (T).

### 2.4 Non-custom prediction module

This module employs existing computational models to facilitate rapid identification, including nine methods, i.e. CSCRSites (Wang *et al.* 2019), HCRNet (Yang *et al.* 2022), DeepBind (Alipanahi *et al.* 2015), iCircRBP-DHN (Yang *et al.* 2021), PrismNet (Sun *et al.* 2021), HDRNet (Zhu *et al.* 2023), CircSLNN (Ju *et al.* 2019), CRIP (Zhang *et al.* 2019), iDeep (Pan and Shen 2017). These methods are used to predict RBP binding sites in circRNA, linear RNA, and RNA from various cellular contexts. A brief description of these state-of-the-art methods can be found in Supplementary Section S2 and Supplementary Table S1.

To improve the confidence of the prediction, EnrichRBP enables users to execute all methods for either circular or linear RNA with a single button click. The platform provides pre-trained model files for 37 circRNA and 31 linear RNA types. Additionally, a versatile model trained on a combination of 37 RBPs is available, designed to predict binding sites of unknown RBP types in circRNAs. While this model does not limit the RBP type, selecting a specific RBP may yield more accurate results. The available classifiers, trained with a broad selection of public RNA–RBP binding sites datasets, offer effective solutions for users lacking labeled training data. These ready-to-use classifiers significantly reduce memory usage and computational time.

### 2.5 Custom prediction module

EnrichRBP offers a customized prediction module that allows users to initiate classification attempts and obtain a series of interpretable analyses at the results reporting stage. In this module, EnrichRBP implements custom wrapper functions at each step to provide security and convenience of computation. Each wrapper is tailored to perform the following tasks:

#### 2.5.1 Comprehensive encoding strategies

To capture the characteristics of RNA sequences involved in RBP interactions, EnrichRBP integrates various sequence feature representation methods. These include dynamic semantic information, static semantic information, RNA physicochemical properties, and RNA secondary structure information.

- Dynamic semantic information captures long-distance nucleotide dependencies in RNA sequences. We developed RBPBERT, a specialized BERT-based model, specifically designed for predicting RBP interactions. Inspired by transformer-based models (Ji *et al.* 2021, Yang *et al.* 2024), RBPBERT’s training process begins by converting RNA sequences into a series of tokens to enhance their linguistic properties. This process involves selecting token lengths—3, 4, 5, and 6-mers—determined to be most effective in generating semantic sequence information through rigorous experimental validation. For instance, with a token length of 3, the ‘AAGACU’ fragment is segmented into AAG, AGA, GAC, ACU, covering all permutations of 3-mers. RBPBERT was subsequently pre-trained on a collection of masked RNA sequences aimed at protein interactions. These sequences, designed with intentional omissions, challenge the model to infer missing segments, thus learning the contextual relationships critical to RNA–protein interactions. To analyse the weighted sum of feature vectors across all tokens, we employ a multi-head self-attention mechanism that captures inter-token dependencies, allowing for exhaustive encoding of specific words. The self-attention module functions as follows:
  $$\begin{array}{c} \text{Multihead}\left(Q,K,V\right)=\text{Concat}\left({\text{head}}_{1},\ldots ,{\text{head}}_{n}\right)W^{O}\\ {\text{head}}_{i}=\text{Attention}(Q,K,V)\\ \text{Attention}\left(Q,K,V\right)=\text{softmax}\left(\frac{QK^{T}}{\sqrt{d_{k}}}\right)\cdot V\\ \{\begin{array}{c} Q=A\cdot W_{i}^{Q}\\ K=A\cdot W_{i}^{K}\\ V=A\cdot W_{i}^{\nu } \end{array} \end{array}$$
  where *Q* (Query), *K* (Key), and *V* (Value) represent linear projections of input sequences while $W^{O}$ denotes the output projection matrix. Matrices $W_{i}^{Q},\;W_{i}^{K}$, and $W_{i}^{V}$ serve as parameter matrices for the *i*th attention head. Here, $d_{k}$ indicates the dimensionality of the key vectors, and matrix *A* is the weight matrix applied to the concatenated output of the attention heads. In the fine-tuning stage, the original head of the pre-trained model is replaced with a randomly initialized classification layer. The last hidden layer’s state in RBPBERT is used to capture the dynamic semantic information of the input sequences. For a detailed discussion of the model parameters, please refer to 3UTRBERT (Yang *et al.* 2024) and DNABERT (Ji *et al.* 2021).
- To derive static local semantics from RNA sequences, our platform employs FastText, GloVe, Word2Vec, and Doc2Vec models, following methodologies similar to those previously described.
- Physicochemical features serve as crucial descriptors for RNA. Recognizing the established link between the physicochemical properties of RNA sequences physicochemical properties and their functional roles (Wang *et al.* 2019, Jia *et al.* 2020, Yang *et al.* 2021), we investigated seven distinct coding techniques to capture a diverse array of RNA physicochemical characteristics. Detailed descriptions of these physicochemical properties and their t-SNE visualizations can be found in Supplementary Section S3 and Supplementary Fig. S15.
- Structure-based features are several descriptors that depict the established RNA secondary and tertiary structures, which are decisive for many RNA functions (Koodli *et al.* 2019). Specifically, the structure of RNA sequences is inferred from their secondary structures, primarily characterized by nucleotide base pairing patterns. Indeed, using RNAplfold, we calculated position-specific probabilities for each input sequence (including paired regions, hairpin loops, multi-loops, inner loops, and external elements), thereby capturing secondary structure information.

To identify the optimal encoding method, we combined all encoding features and analysed all possible combinations to determine the best-performing feature set. This process includes the following steps: (a) Using a previously published feature ranking method (Tong and Liu 2019), we ranked all combinations of RNA encoding features; (b) We created feature subsets by iteratively removing the lowest-performing feature; (c) We extracted embedded features using the ResNet mentioned in Section 2.5.3; (d) We obtained predictive results by inputting the embedded features into a downstream classifier. Ultimately, our research indicates that decomposing sequences into 3-mer forms and utilizing dynamic semantic information from RBPBERT yields the best results. Through these efforts, we ultimately developed 28 unique methods of sequence feature representation, detailed in Supplementary Section S3 and Supplementary Table S8.

#### 2.5.2 Selection of significant features

To address the potential issue of increased computational cost in subsequent models due to high feature dimensions (Verleysen and François 2005), we have integrated 18 feature selection methods. These include information-theoretic approaches, similarity-based methods, sparse learning techniques, and statistical methods. Users can choose an appropriate feature selection method to reduce the dimensions of the generated feature encoding matrix. For detailed information, please refer to Supplementary Section S4 and Supplementary Table S9.

#### 2.5.3 Model construction and training

We then feed significant features into the classical machine learning and advanced deep learning models to identify RBP binding sites. To train and evaluate the model, the sequence dataset on the web server is randomly split into a training and test set in a 7:3 ratio. Specifically, EnrichRBP incorporates fundamental classifiers from various machine learning approaches, including Logistic Regression, K-nearest neighbor, Decision Tree, Gaussian NB, Bagging, Random Forest, AdaBoost, gradient boosting, SVM, Linear Discriminant Analysis (LDA), and ExtRa Trees. Besides, EnrichRBP comprises convolutional neural network (CNN), recurrent neural network (RNN), multilayer perceptron (MLP), and residual network (ResNet). Furthermore, EnrichRBP allows users to select multiple models simultaneously, and to train and compare their performance on the same dataset. Users can independently build and train models based on their unique needs, facilitating the discovery of RNA–RBP interactions through customized analytical methods. Supplementary Section S5 and Supplementary Table S10 provide detailed descriptions of the 15 methods mentioned in this module, outlining their functions and applications in predicting RNA–RBP interactions.

### 2.6 Result reporting

In this module of the platform, EnrichRBP provides 27 visualizations of the analytical results from the two main modules, designed to illustrate model performance and interpretability, as detailed in Supplementary Section S6 and Supplementary Tables S11–S14. All results data and figures are publicly accessible and can be downloaded by users. Notably, submitters receive an instant notification on the completion of each task.

### 2.7 Webserver implementation

The development environment for EnrichRBP utilizes Ubuntu 18.04.4 and Python 3.7.16, with four Quadro RTX 6000 GPUs for training the dynamic semantic model. The EnrichRBP server operates on a Linux server with two AMD EPYC 7B12 3.80 GHz CPUs featuring 64 cores supporting two threads each, 128 GB of RAM, and an NVIDIA GeForce GTX 3090 GPU. We designed a user-friendly interface to align with common user practices. The technical stack includes Nginx for web deployment, MySQL for database management, Django for backend operations, jQuery for front-end development, and Bootstrap 5 for enhancing interface esthetics. The web server supports Mac, Linux, and Windows operating systems and ensures stable performance on popular browsers such as Edge, Firefox, Safari, and Google Chrome. EnrichRBP is freely available to all users without the need for login and can be accessed at https://airbp.aibio-lab.com/.

## 3 Results

### 3.1 Comparison of RBP binding site identification methods for circular and linear RNAs

**Figure 2.**
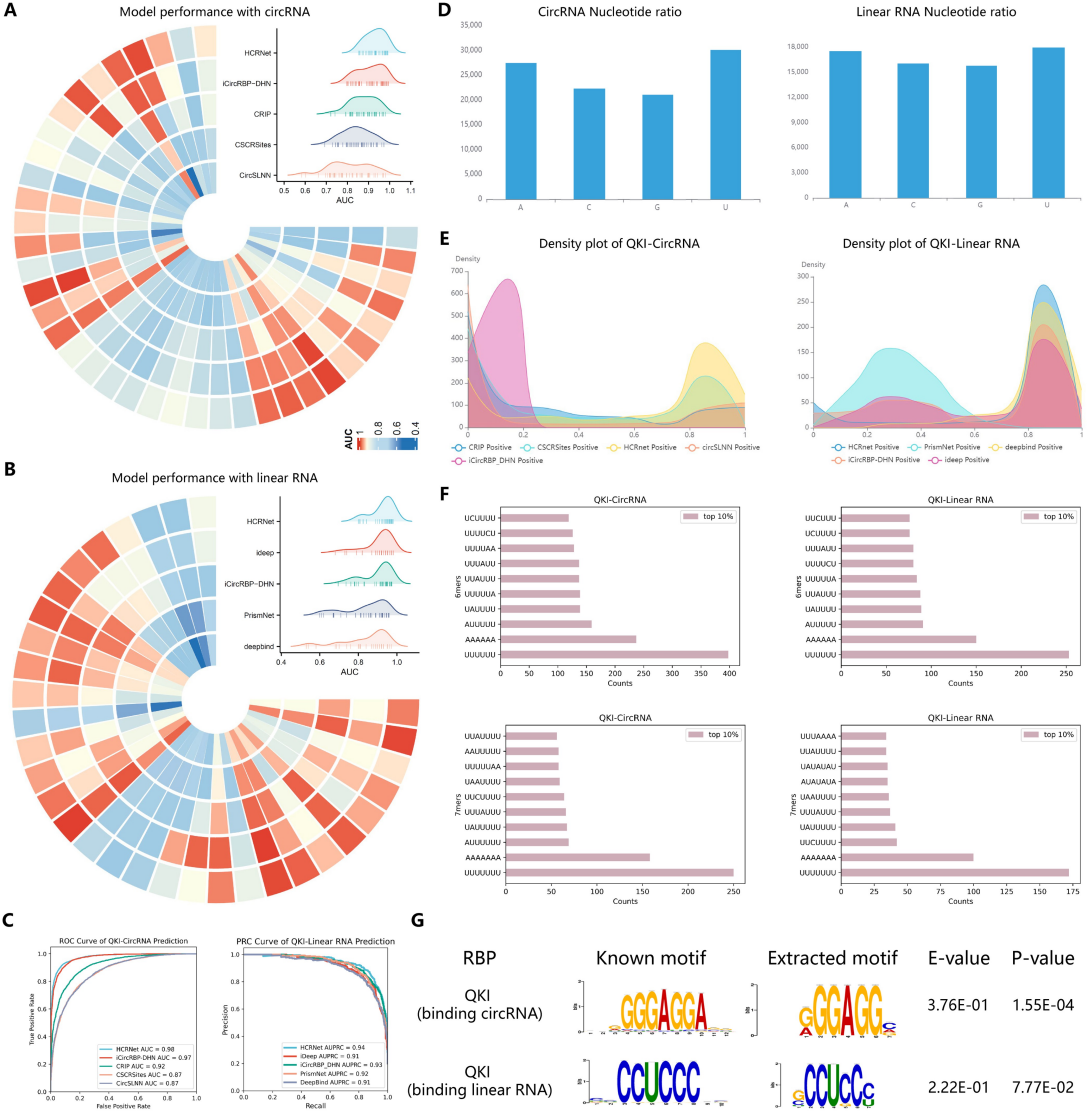
The prediction analysis of different models in EnrichRBP. (A and B) The Circos heatmaps and Mountain plots of the AUC scores for each of the five methods on the 37 and 31 RBP datasets, respectively. (C) The ROC curve representing the model’s performance in predicting QKI binding sites on circRNA datasets and the PRC curve showcasing the prediction performance for QKI binding sites on linear RNA datasets. (D) Histogram of the nucleotide frequency distribution of the target set sequences. (E) Density distribution of the prediction confidence for the different deep-learning models. (F) The 6, 7-mer counts in the top 10% of the positive samples for QKI, ranked by their prediction scores. (G) The motif analysis on QKI datasets using the MEME method.

Several computational methods have been developed to identify RBP binding sites in datasets. We evaluated the performance of eight state-of-the-art computational methods, namely HCRNet (Yang *et al.* 2022), iCircRBP-DHN (Yang *et al.* 2021), CRIP (Zhang *et al.* 2019), CSCRSites (Wang *et al.* 2019), CircSLNN (Ju *et al.* 2019), iDeep (Pan and Shen 2017) PrismNet (Sun *et al.* 2021), and DeepBind (Alipanahi *et al.* 2015) on 37 circular RNA (circRNA) and 31 linear RNA datasets. We used the area under the curve (AUC) as the performance metric to assess all methods. Each RBP binding site dataset was processed using a uniform pipeline (Yang *et al.* 2022) to ensure accuracy, consistency, and fairness of comparison. Figure 2A and B displays Circos and Mountain plots, respectively, showing the experimental results of the different methods on the circRNA and linear RNA datasets. HCRNet consistently achieved the highest prediction performance on both types of RNA datasets. In addition, QKI, a protein that binds to RNA, is a critical pre-mRNA alternative splicing regulator of cardiac myofibrillogenesis and contractile function (Chen *et al.* 2021). We analysed the predictive performance of QKI in binding with the circRNA datasets. Specifically, we employed five models (HCRNet, iCircRBP-DHN, CRIP, CSCRSites, and CircSLNN) for the circRNA and linear RNA datasets and provided the receiver operating characteristic (ROC) curve in Fig. 2C for each RNA dataset type. The ROC analysis demonstrated that HCRNet had a higher true positive rate (TPR) compared to the other methods, indicating greater sensitivity in identifying RBP binding sites. We also assessed five models for the linear RNA dataset: HCRNet, iCircRBP-DHN, iDeep, PrismNet, and DeepBind. Since the linear RNA dataset has an imbalanced positive-to-negative ratio (1:4), precision–recall curves (PRC) were used to evaluate model performance. The PRC results further highlight that HCRNet outperforms competing methods, showcasing its robustness and reliability in predicting QKI binding sites across different RNA types. Given that existing platforms (RNAincoder, catRAPID omics, and catRAPID omics v2.0) fail to explicitly report accuracy metrics for RNA interactions with various RBPs, and considering the limited input capacity (only 10 samples at a time), we further conducted a comparison experiment to evaluate the false positive rate rather than the AUC. The test circRNA sequences were derived from 1000 non-binding samples (negative samples) from QKI. Compared to the best-performing method, HCRNet, which achieved a false positive rate of 4.1%, RNAincoder, catRAPID omics, and catRAPID omics v2.0 exhibited higher false positive rates of 6.7%, 8.4%, and 7.0%, respectively. These results highlight the challenges faced by platforms employing generic prediction models, as their performance is less robust, leading to higher false positive rates in certain datasets. Figure 2D shows the dataset statistics of the target sequences to be predicted as a histogram chart, highlighting the nucleotide composition differences. To understand the prediction preferences of each model and therefore anticipate the binding of QKI preferences to different transcripts, we visualized the density distribution of the prediction confidence of the different models in Fig. 2E. For circRNA, the prediction confidence of iCircRBP-DHN was concentrated at 0.18 on the *x*-axis. In contrast, the other four models (CRIP, CSCRSites, HCRNet, and CircSLNN) exhibited higher prediction confidence, indicating a better classification performance on the example dataset compared to iCircRBP-DHN. For linear RNA, PrismNet had the lowest prediction confidence for the QKI binding site, compared to the other models. Variations in classification performance may come from differences in the quality of individual CLIP datasets, such as false negatives or false positives, and the complexity of RBPs due to their participation in complexes (Ghanbari and Ohler 2020). In addition, we calculated and display in Fig. 2F the 6–7 mer counts in the top 10% of the positive samples for QKI binding, ranked by prediction score, which intuitively shows the preferences for enrichment of nucleotide fragments in the dataset. Notably, the 6–7 mer had a remarkable similarity, with significant U enrichment across datasets, suggesting a potential role in predicting the binding domain of QKI. The comparison of 8–9 mer counts in the QKI datasets can be seen in Supplementary Fig. S1. To further indicate biological interpretability, we used a MEME mining approach (Bailey et al. 1994) to identify conserved sequence motifs in RNA sequences (Fig. 2G). In addition, EnrichRBP can identify structure preferences as depicted in Supplementary Fig. S18. The detected sequence motifs were compared to the JASPAR database (Castro-Mondragon *et al.* 2022) using the TOMTOM algorithm (Gupta *et al.* 2007), identifying motifs ‘GGGAGG’ and ‘CCUCCC’ in the QKI dataset. However, these motifs ranked low in the 6-mer counts, affecting identification (Fig. 2F). Therefore, leveraging the MEME method can help discover potential patterns of binding sequences associated with biological functions.

### 3.2 Identification and analysis of protein–RNA interaction across different cellular environments

**Figure 3.**
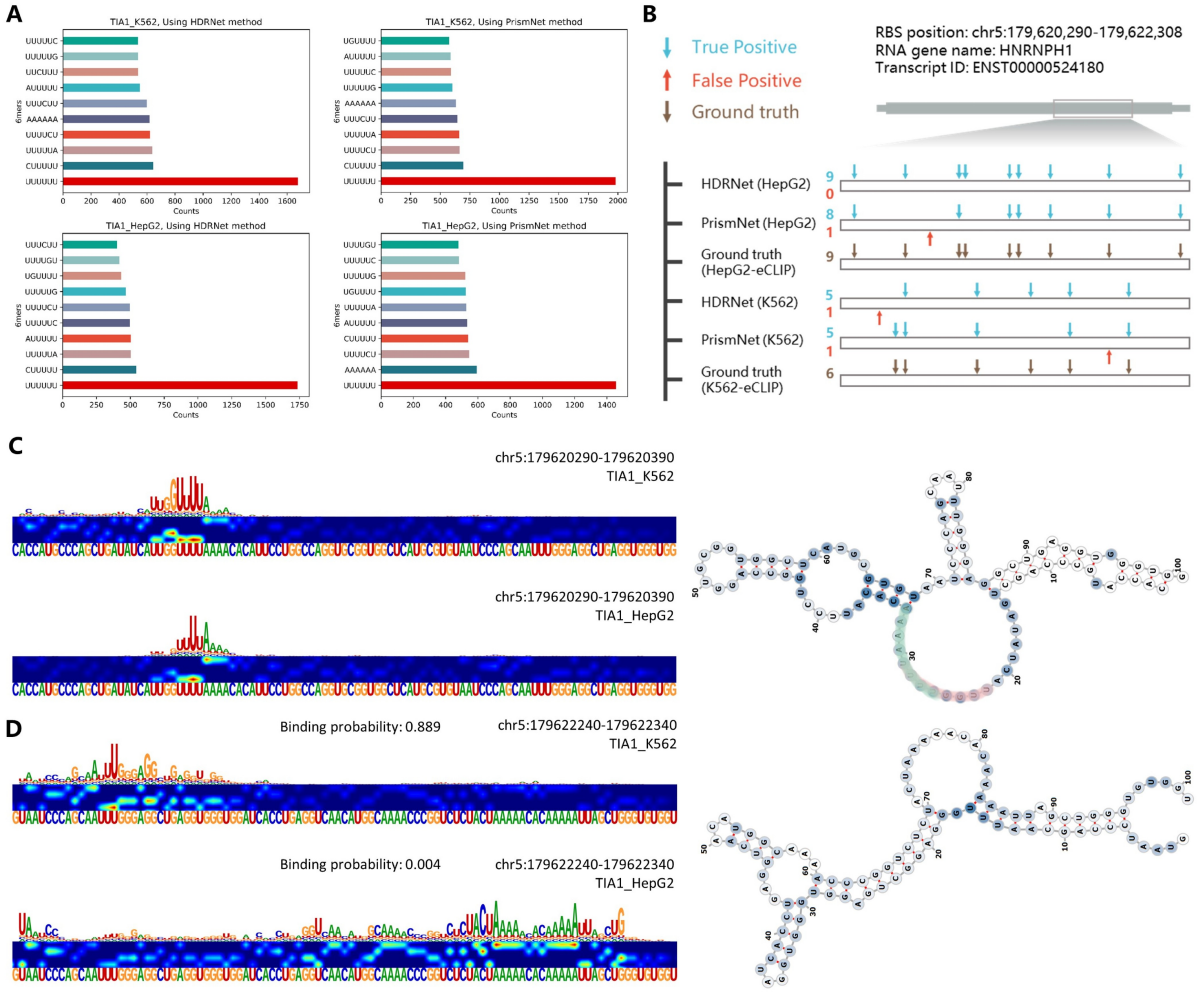
(A) The 6-mer counts in the top 10% of the positive samples for TIA1 in K562 and HepG2 cell lines, using HDRNet and PrismNet methods, ranked by their prediction scores. (B) Predicted versus observed binding sites of TIA1 on the HNRNPH1 transcript. (C and D) TIA1 binding probabilities and saliency maps in HepG2 and K562 cells and the visualization of the RNA secondary structure.

To investigate the identification function of the EnrichRBP platform across different cell lines, we analysed the interaction of 261 RBPs in the K562 cellular environment to the TIA1 transcript as input, a transcript with RNA structure characterized in prior studies (Sun *et al.* 2021). Results from this analysis are detailed in Supplementary Table S2 and are highlighted in Fig. 3A showing consistency between the 6-mer motifs enriched in TIA1 binding sites across the different cell lines. The motifs predominantly found were sequences ‘UUUUUU’, ‘UA’, and ‘UUG’, suggesting the potential roles of these motifs in TIA1 binding affinity. In a comparative analysis using HDRNet (Zhu *et al.* 2023) and PrismNet (Sun *et al.* 2021), HDRNet accurately predicted all nine TIA1 binding sites in the HNRNPH1 transcript in HepG2 cells with no false positives, and it identified five correct sites and one incorrect site in K562 cells (Fig. 3B). PrismNet, detected eight out of the nine sites in HepG2 cells and five out of the six sites in K562, capturing one site in K562 missed by HDRNet. The source data are provided in Supplementary Table S3. TIA1 binds with high affinity to multiple U-sequence motifs in single-stranded regions (Sun *et al.* 2021). As shown in Fig. 3C, HNRNPH1 has a high affinity for TIA1 in both K562 and HepG2 cell lines, and the ‘UUU’ motifs identified by the saliency map corroborate the ability of the algorithm to identify potential RBP binding sites. The secondary structure of the RNA is shown on the right of 3D, with the predicted binding regions in K562 and HepG2 cell lines highlighted in pink and green, respectively. As depicted in Fig. 3D, we understand that HDRNet was able to identify dynamic binding events on the same transcript in different cells, with a binding probability of 0.889 in K562 cells and 0.004 in HepG2 cells, indicating diverse gene expression in different cells. Overall, our results emphasize the importance of method selection based on specific predictive confidence levels and cellular environments.

### 3.3 EnrichRBP shows the effects of mutations in RBP binding sites

**Figure 4.**
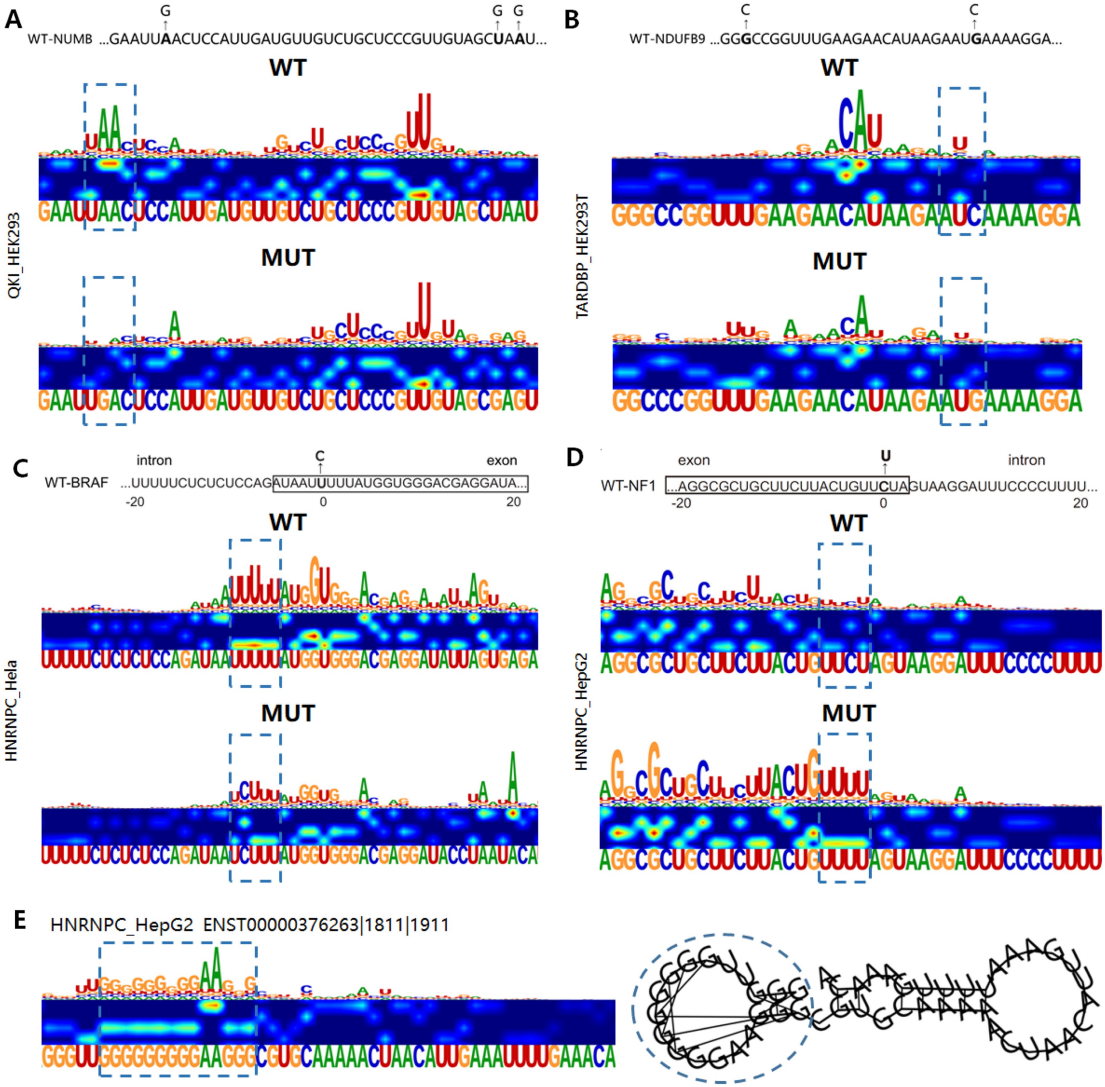
Assessing the impact of sequence variants using saliency maps. Sequences of the wild-type and mutant constructs, where mutations are shown in bold letters and the corresponding saliency maps for QKI, TARDBP, and HNRNPC. WT binding sites before and potential binding sites after mutation are shown by dashed boxes. (WT) wild-type; (Mut) mutant. (A and B) Potential variants of QKI and TARDBP binding events in the *NUMB* and *NDUFB9* genes, respectively. (C and D) The exonic mutations of the HNRNPC binding targets near splice sites for genes *BRAF* and *NF1*, respectively. (E) Identified G-quadruplex structures.

Recent studies have demonstrated that mutations near splice sites can affect RNA splicing and contribute to human diseases as they disrupt RBP interactions (Scotti and Swanson 2016). In our study, we show that mutations near the 3′ splice site of intron 12 abolished the QKI binding site. As shown in Fig. 4A, the saliency map of the wild-type sequence demonstrated robust binding activity, whereas the mutated sequence completely lost this activity. Additionally, mutations in *NDUFB9*, which encodes the I subunit of the mitochondrial complex, result in mitochondrial complex I deficiency. The expression of wild-type *NDUFB9* in patient-derived cells mitigates this disease (Haack *et al.* 2012). Our analysis in HEK293T cells also revealed that mutations in NDUFB9 affect TARDBP binding near the mutated sites, potentially contributing to disease pathogenesis (Fig. 4B). Further examples include a U to C mutation near the splice site of the proto-oncogene *BRAF*, which reduces binding of HNRNPC (Fig. 4C), and a C to U substitution near the 5′ splice site of the *NF1* gene, which enhances binding of HNRNPC (Fig. 4D). These mutations correlate with established neurological disorders (Viskochil 2002). RNA structural changes due to sequence mutations are discussed in detail in Supplementary Section S11. Furthermore, studies have shown that the formation of G-quadruplexes (G4) in cancer cells is associated with synthetic lethality, making G4 structures a potential therapeutic target for human diseases (Varshney *et al.* 2020). EnrichRBP effectively identifies critical HNRNPC and AARS binding domains, including those associated with G4 structures (Fig. 4E and Supplementary Fig. S2). HNRNPC shows a preference for G4 structures as binding motifs in RNAs, suggesting the platform’s potential for uncovering biologically significant insights (Herviou *et al.* 2020).

### 3.4 EnrichRBP can more accurately identify RBP binding sites through customized prediction modules

**Figure 5.**
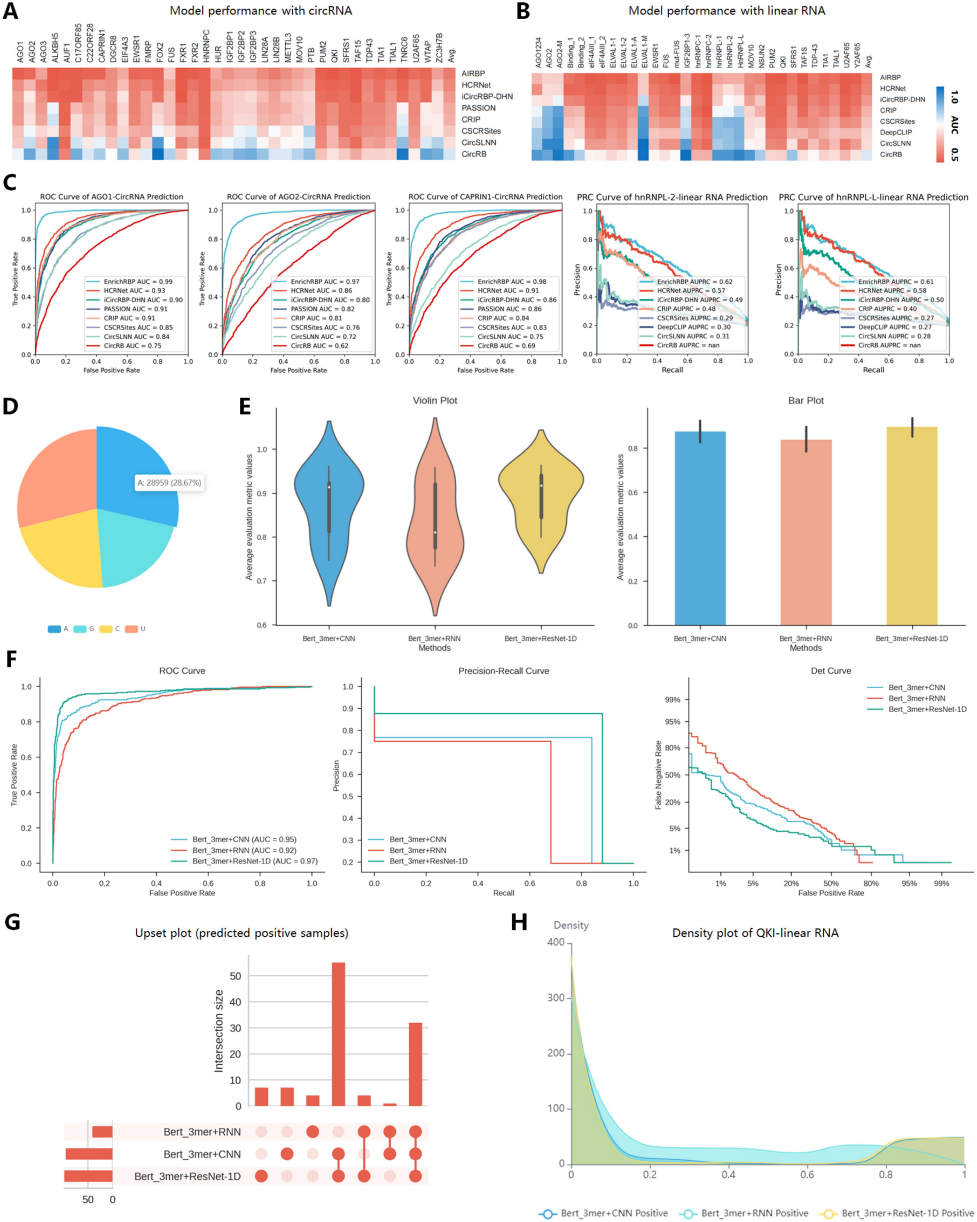
The prediction analysis investigating different models of EnrichRBP. Analysis of results using customized methods to predict QKI binding sites. (A and B) The heatmap of the respective AUC scores of prediction of EnrichRBP and other baseline methods. (C) The prediction ROC and PRC curve of the five RBP datasets using EnrichRBP and the other methods. (D) Pie chart of the number and proportion of nucleotide composition in the target set sequences. (E) The violin plot and bar chart of the performance of the different methods, where ‘average evaluation metric value’ refers to the average of eight evaluation metrics. (F) ROC, PR, and Det curves of the different models. (G) Upset plot to express the relationship of prediction results for the different models (only sequences predicted as binding sites are shown). (H) Density distribution of prediction confidence for different deep learning models.

To evaluate the feature characterization capabilities of EnrichRBP, we utilized 37 circRNA and 31 linear RNA datasets from the study by Yang *et al.* (2022) as benchmarks. EnrichRBP’s performance was compared with eight well-established algorithms: HCRNet (Yang *et al.* 2022), iCircRBP-DHN (Yang *et al.* 2021), Passion (Jia *et al.* 2020), CRIP (Zhang *et al.* 2019), CSCRSites (Wang *et al.* 2019), CircSLNN (Ju *et al.* 2019), CircRB (Wang and Lei 2021), and DeepCLIP (Grønning *et al.* 2020). To ensure a fair comparison, we applied the optimal parameters for each algorithm as specified in their respective studies and performed five-fold cross-validation. As illustrated in Fig. 5A and B, EnrichRBP demonstrated superior performance, over the other methods on 34 of the 37 circRNA datasets and 27 of the 31 linear RNA datasets. This indicates EnrichRBP’s robust feature characterization capabilities, which perform consistently well across various dataset scales and types. The enhanced performance and stability of EnrichRBP can be attributed to using RBPBERT mentioned in the custom prediction module to extract contextual semantic information, which enhances prediction accuracy. Detailed results for all methods are available in Supplementary Tables S4 and S5. Furthermore, Fig. 5C presents the ROC and PRC curves comparing EnrichRBP with seven other algorithms on the binding scores of the AGO family (AGO1 and AGO2), CAPRIN1 to circRNAs, and the hnRNPL family (hnRNPL-2 and hnRNPL-L) to linear RNAs. Notably, EnrichRBP showed superior performance compared to the second-best algorithm, HCRNet, achieving AUROC improvements of 6% for AGO1, 7% for AGO2, and 11% for CAPRIN1; and the AUPRC indicators improved by 3% (hnRNPL-2) and 5% (hnRNPL-L), respectively. Further, we employed the QKI dataset, comprising 1200 binding site sequences (positives) and 4800 non-binding site sequences (negatives), to illustrate protein–RNA interaction identification analysis. We initially partitioned the dataset into a training and test set using a 4:1 ratio. Additionally, we added another 1000 sequences for prediction that were subsequently processed through our online platform to demonstrate the data analysis capabilities and prediction functionalities of EnrichRBP. We selected three models (Bert_3mer+CNN, Bert_3mer+RNN, and Bert_3mer+ResNet) for training and evaluation on the RBP binding dataset. Figure 5D displays the dataset statistics for the QKI dataset using a pie chart to highlight the nucleotide composition differences. For model prediction analysis, as depicted in Fig. 5E, violin and bar plots show the prediction performances of these models based on an average of eight evaluation metrics. Box and point plots, are available in Supplementary Fig. S3. The three models demonstrated slight variations in the evaluation metrics, and all performed with high scores. Bert_3mer+ResNet had an excellent performance with AUC (0.967), ACC (0.943), Recall (0.870), *F*1-score (0.857), MCC (0.822), precision (0.844), specificity (0.961), and AP (0.911). Scores for the other models can be found in Supplementary Table S6 of the Supplementary Materials. For a more comprehensive evaluation, Fig. 5F illustrates the ROC, precision–recall (PR) curves, and detection error tradeoff (DET) curves of the compared models. As seen in the figure, Bert_3mer+ResNet achieved the highest AUC value and superior performance on PR and det curves, confirming its superiority over the other models. Moreover, we included an Upset plot in Fig. 5G, revealing that most positive samples are successfully predicted from the overlapping predictions of the compared models. Furthermore, Fig. 5H visualizes the density distribution of the prediction confidence for the different models, which indicates that all three models exhibited high prediction confidence on both positive and negative samples, demonstrating their robust classification ability on the dataset.

In addition, we integrated diverse biological features, as well as the ReliefF feature selection method to effectively capture relevant feature information. Subsequently, we utilized conventional machine learning algorithms to identify RNA–RBP binding sites. Detailed information and the result visualization can be found in Supplementary Section S8 and Supplementary Figs S4–S8 of the Supplementary Material.

### 3.5 Feature analysis and visualization

**Figure 6.**
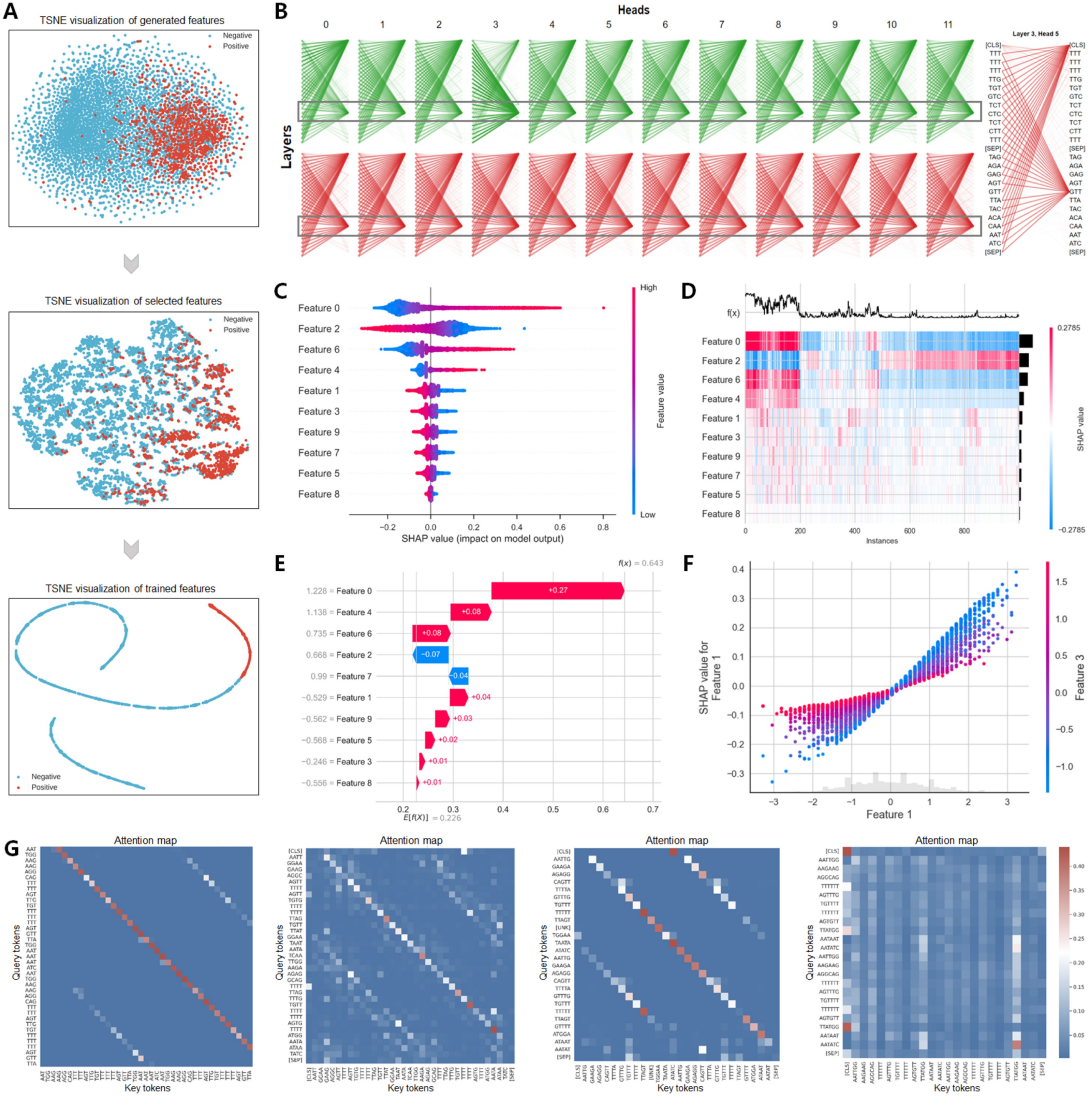
The visualization of feature analysis and model interpretation by EnrichRBP. (A) The t-SNE flow graph shows the clustering effect of the output of the different intermediate layers of the EnrichRBP platform. (B) Visualization of attention distribution using dynamic global contextual embedding, with attention concentrated in T/G-rich regions. (C and D) The top 10 features that have the greatest impact on RBP binding site identification, revealing how they act in predicting non-binding sites and bindings sites, respectively. (E) Waterfall plot for explanations of individual predictions. (F) Beeswarm plot for the interaction between feature 3 and the other feature. (G) Attention maps illustrating that both local and global contextual semantics can be extracted.

To elucidate the insights gained during model training, we provide a detailed feature analysis and visualization, as shown in Fig. 6. Figure 6A illustrates the entire learning process of the multi-source biological information. We projected the hidden layer outputs from the machine learning-based sequence network onto a 2D plane using t-SNE. Initially disorganized, these projections evolved into complex hierarchical attributes through iterative training and feature selection, enriching each nucleotide’s contextual information. Moreover, Fig. 6B shows the attention distribution across different heads within two BERT layers. Each row represents a layer with 12 heads, where attention scores increasingly focus on key RNA sequence regions, including [CLS] and [SEP] tokens. The platform identified a significant RBP-propensity binding site, confirmed by the saliency map in Supplementary Fig. S9. The hidden semantic relationship in the ‘GTT’ token highlighted contributions from various regions. For feature analysis, Fig. 6C displays the top ten features that affected the RNA-RBP binding site recognition. For instance, a low value for Feature 0 typically classifies samples as non-binding sites, whereas a low value for Feature 2 suggests binding sites. Furthermore, Fig. 6D is a heatmap plot, using the SHAP values of the features to group samples with the same cause and the same model output in a supervised clustering manner. The outputs of the models are displayed above the heatmap matrix, and the global importance of each model’s input is displayed in a bar on the right side of the heatmap. Additionally, Fig. 6E illustrates each feature’s contribution to the output, showing how the initial value *E*(*x*) changes towards the predicted value *f*(*x*). The interaction between features was more significant than individual feature impacts. Figure 6F shows that as Feature 1 increased from −3 to 3, its SHAP value increased almost linearly, indicating a strong positive correlation with model predictions. Smaller values of Feature 3 showed increased sensitivity to changes in Feature 1, revealing a strong correlation between Feature 1 and Feature 3. For additional feature output plots, please refer to Supplementary Fig. S10. To enhance the interpretability of our trained RBPBERT, we calculated the importance of bases at each position in a user-provided sequence based on the attention matrix of the model and visualized it using normalized attention scores. In Fig. 6G, we present attention maps of the last attention heads for RBPBERT sequences divided into 3, 4, 5, and 6-mers, respectively. In sequences split into 3, 4, or 5-mers, attention was primarily aligned along the diagonal, indicating the model’s emphasis on the sequential order of input and each marker’s focus on itself and its immediate neighbors. In the 6-mer segmentation, attention displayed a dispersed pattern, suggesting the model’s ability to learn complex dependencies between non-adjacent tokens across different layers and attention heads. This attention graph illustrates the extent of the model’s focus on each marker during processing, shedding light on how it discerned both local and global relationships within the RNA sequence. Detailed attention maps for the remaining attention heads are available in Supplementary Figs S11–S14.

## 4 Conclusion

To facilitate a comprehensive analysis of the binding of RBPs to RNA molecules, we have developed EnrichRBP, a novel RNA-binding site prediction platform with multiple functionalities. Firstly, it identifies RNA–RBP binding sites using both conventional and advanced prediction methods. Secondly, it offers users a rich array of RNA coding features, including sequence dynamics, static semantic information, and physicochemical and structure-based features. It provides robust representations of RNA–RBP interactions through well-established machine-based or deep learning embedding strategies. This enables researchers to select the most effective prediction modalities using detailed visualization via graphs. Unlike other web servers focusing solely on identifying RNA–RBP binding, EnrichRBP combines rapid prediction, RNA sequence characterization, feature selection, model training, and prediction, and thorough analysis and interpretation of results. In comparison, RBPsuite provides useful predictions, but with limited accuracy and biological interpretability. Additionally, it fails to predict RBP binding affinities in specific cell types, where RBP binding can vary significantly due to cellular context (Sun *et al.* 2021). EnrichRBP addresses many of the above limitations of RBPsuite and other similar platforms.

In the future, EnrichRBP’s functionality will be further expanded to include the prediction of RNA structural motifs, building on the capabilities demonstrated by tools like BEAM (Pietrosanto *et al.* 2018) and GraphProt (Maticzka *et al.* 2014). Another promising direction is its application in understanding RBP-mediated post-transcriptional regulatory mechanisms, such as alternative splicing and mRNA stability. For instance, EnrichRBP could assess whether RBP binding sites are associated with splicing by examining their presence at key regions, such as the 5′ splice sites of exons. Additionally, we plan to investigate the correlation between predicted RBP binding sites and RNA half-life, similar to the approach in PrismNet (Sun *et al.* 2021), to further elucidate the role of RBPs in RNA stability. We believe EnrichRBP will serve as a valuable resource for researchers studying RBPs, providing insights into the functional roles in post-transcriptional regulation.

## Author contributions

Yubo Wang (Data curation-Equal, Methodology-Equal, Software-Equal, Visualization-Equal, Writing—original draft-Equal), Haoran Zhu (Investigation-Equal, Methodology-Equal, Writing—original draft-Equal), Yansong Wang (Data curation-Equal, Methodology-Equal, Software-Equal), Yuning Yang (Data curation-Equal, Methodology-Equal, Writing— original draft-Equal), Yujian Huang (Investigation-Equal, Validation-Equal, Visualization-Equal), Jian Zhang (Supervision-Supporting, Validation-Equal, Visualization-Equal), Ka-chun Wong (Conceptualization-Supporting, Validation-Equal, Writing—review & editing-Equal) and Xiangtao Li (Conceptualization-Lead, Validation-Equal, Visualization-Equal, Supervision-Lead, Writing—review & editing-Equal)

## Supplementary Material

btaf018_Supplementary_Data

## Data Availability

As an online platform for RNA-RBP binding site identification and analysis, EnrichRBP is freely available to all users at https://airbp.aibio-lab.com/. All codes used for data analysis and manuscript preparation, alongside a description of necessary steps to reproduce the results, can be found in a GitHub repository accompanying this manuscript: https://github.com/wangyb97/EnrichRBP. In terms of the circRNA sequences, 37 circRNA datasets were extracted from the circRNA interactome database (https://circinteractome.nia.nih.gov/). We also utilized a collection of 740 full-length circRNAs from CRIP. In addition, linear RNA datasets were downloaded from iCount (http://icount.biolab.si/), including 31 experimental CLIP datasets with 19 RBPs involved in splicing. Moreover, we collected 261 RBP binding site datasets from multiple databases, including 172 RBPs constructed in the K562, HepG2, HEK293, HEK293T, HeLa and H9 cell lines. These datasets include 65 CLIP-seq datasets for 61 RBPs from the http://111.198.139.65/index.html database and 196 eCLIP datasets for 111 RBPs from the ENCODE project.
