# Supplementary material for: EnrichRBP: an automated and interpretable computational platform for predicting and analysing RNA-binding protein events
Source: Bioinformatics. 2025 Jan 13;41(1):btaf018. doi: 10.1093/bioinformatics/btaf018 (PMC11783304; doi:10.1093/bioinformatics/btaf018)
Supplement: btaf018_Supplementary_Data [file btaf018_supplementary_data.zip › efe76_Supplement Data_v4_bio.pdf]

EnrichRBP: an automated and interpretable computational platform for predicting and analyzing RNA-binding protein events
Supplementary Information

Yubo Wang [1], Haoran Zhu [1], Yansong Wang [1], Yuning Yang [2], Yujian Huang [3*], Jian Zhang [4*], Ka-chun Wong [5] and Xiangtao Li [1*]

[1]School of Artificial Intelligence, Jilin University, 130012 Changchun, China,

[2]Information Science and Technology, Northeast Normal University, 130012 Changchun, China,

[3]College of Computer Science and Cyber Security, Chengdu University of Technology, 610059 Chengdu, China,

[4]School of Computer and Information Technology, Xinyang Normal University, Xinyang, 464000 China and [5]Department of Computer Science, City University of Hong Kong, Hong Kong SAR.

Section S1: Inputs and outputs of EnrichRBP

Inputs of EnrichRBP

EnrichRBP serves as an advanced automated platform designed to predict RNA-RBP interactions, beginning with the submission of RNA sequences in the FASTA format. Each RNA sequence, composed of nucleotides A, G, C, U, or T, can be of any length. However, to optimize computational efficiency and resource allocation, the platform restricts the total number of sequence entries to 1000. Prior to advancing through the subsequent modules, EnrichRBP rigorously validates the FASTA format of the RNA sequences, ensuring data integrity and seamless functionality. For each sequence, the length is standardized to 101 nucleotides. Sequences shorter than 101 nucleotides are extended symmetrically from the center, while those exceeding 101 nucleotides are truncated evenly from both ends. This preprocessing step guarantees that the input data is compliant, facilitating smooth progression to the next module.

In the Non-Custom Prediction module, users are required to select both the prediction method and the specific type of RNA-binding protein (RBP). In the Custom Prediction module, users can submit transcriptome sequence data and choose an RNA cloud dataset from our curated collection of RBP binding sites to train the model tailored to the specified RBP binding type. Additionally, users can select features for sequence characterization, including dynamic global features, static local attributes, physicochemical properties, and RNA secondary structure information. A variety of feature selection techniques are employed for dimensionality reduction, followed by the application of machine learning and deep learning models as classifiers. Additionally, EnrichRBP includes user-friendly features such as an 'example' button, which loads sample data automatically, enabling users to experiment with pre-loaded examples. A download button is available for users to save the formatted RNA sequence file, ensuring they can verify data formatting requirements prior to submission.

Outputs of EnrichRBP

The results pages presented by EnrichRBP in its Non-Custom Prediction and Custom Prediction modules exhibit notable differences tailored to specific research needs. In the CircRNA-RBP and Linear RNA-RBP Binding Prediction section of the Non-Custom Prediction module, the results page initiates with a detailed summary of job information, delineating the RNA and RBP types specified by the user. Following this preliminary information, there is a table showing the probabilistic predicted values for RNA-RBP binding sites. A dynamic bar graph then visualizes the count of nucleotides provided by the user, offering a comprehensive overview of the nucleotide composition critical for understanding sequence properties. Subsequently, motif plots are presented, showcasing novel or recurring motifs identified in RNA sequences predicted as RBP-binding sites through the MEME method [1]. These plots are instrumental in elucidating potential binding motifs and understanding their sequence context. Upon completion of all algorithms, EnrichRBP displays the confidence density distribution of the selected method via a density plot, facilitating the precise selection of binding sites by visualizing the confidence levels of predictions. In the RNA-RBP Binding Prediction in Cells module, the choice of prediction method further influences the results page. Selecting the PrismNet [2] method results in an output consistent with the aforementioned description. Conversely, opting for the HDRNet [3] method generates a saliency plot, which elucidates potential binding motifs and sequence attention scores. To optimize the user experience and preserve server resources, saliency plots for the initial three RNA sequences input by the user are automatically generated by default, thereby minimizing the wait time.

The custom prediction module offers users access to a diverse array of visualizations tailored to both traditional machine learning techniques and advanced deep learning methods. Specifically, it provides 18 distinct visualizations for traditional machine learning and 11 specialized visualizations for deep learning, as detailed in Tables S12 and S13 of the EnrichRBP documentation. Users can download all generated files for

further investigation and comprehensive analysis.

The runtime for EnrichRBP is highly variable, contingent upon factors such as the number of uploaded RNA sequences and the selected prediction method, with durations ranging from a few seconds to several hours. Generally, the custom prediction module requires a longer runtime compared to the non-custom prediction module due to the additional computational demands of model training, evaluation, and feature analysis. This variability underscores the importance of selecting appropriate methods and managing computational resources effectively in RNA-RBP binding studies.

Section S2: A brief description of the state-of-the-art methods in the Non-Custom Prediction Module

CSCRSites [4] is a deep learning-based approach to identify cancer-specific circRNA-RBP binding sites using nucleotide sequences only as input.

CRIP [5] consists of a stacked codon-based encoding scheme and a hybrid deep learning architecture where a convolutional neural network (CNN) learns high-level abstract features and a recurrent neural network (RNN) learns long dependency in the sequences.

CircSLNN [6] is driven by pre-trained RNA embedding vectors and a composite labelling model for identifying specific locations of RBP binding sites in circular RNA.

iCircRBP-DHN [7] proposes a new coding scheme by integrating CircRNA2Vec and K-tuple nucleotide frequency patterns to represent the different degrees of nucleotide dependencies. It also utilizes deep multi-scale residual networks, as well as bi-directional gated recursive units (BiGRUs) with self-attention mechanism, to extract both local and global contextual information.

HCRNet [8] is an end-to-end framework for identifying circRNA-RBP binding events. To capture hierarchical relationships, it incorporates multiple sources of biological information to represent circRNAs, including various natural language sequence features. In addition, a deep temporal convolutional network incorporating a global

expectation pool is included to exploit potential nucleotide dependencies in an exhaustive manner to reliably identify RBP binding sites.

DeepBind [9] is a CNN-based deep learning model that predicts RBP binding sites based on RNA sequences only.

iDeep [10] leverages a novel hybrid CNN network and a deep belief network to predict the RBP interaction sites and motifs in RNAs by converting the original data into a high-level abstraction feature space using multiple layers of learning blocks, where the shared representations across different domains are integrated.

PrismNet [2] is a recent study that developed a convolutional neural network (CNN)-based deep learning approach, which effectively incorporates in vivo RNA structural data and RNA-binding protein (RBP) binding data to make precise predictions of RBP binding sites. This method applies an "attention" strategy to precisely identify RBP binding nucleotides. Notably, PrismNet is the first tool designed for dynamic prediction tasks.

HDRNet [3] extracts dynamic global contextual embeddings and in vivo RNA secondary structure representations and then feeds the feature space information into a hierarchical multi-scale residual network to identify RNA-RBP interaction sites in different cell lines or tissues with high accuracy.

Section S3: Description of collected RNA encoding features in the Custom Prediction Module

Physicochemical properties

1. **PseudoKNC (Pseudo K-tuple Nucleotide Composition):** To extract local contextual features from the RNA sequences, PseudoKNC is employed to encode the nucleotide sequences, which can embrace the adjacent information of each nucleotide in the sequences [11]. Specifically, the regular k-tuple is a vector that represents a nucleotide sequence with size of $4k$. The PseudoKNC can be applied

by aggregating the set of k-tuples that contains all tuples consisting of less than or equal to k nucleotides. It can be defined as follows:

$$V_i = [f_1^{i-tuple}, f_2^{i-tuple}, f_3^{i-tuple}, \ldots, f_t^{i-tuple}, \ldots, f_{4^i}^{i-tuple}](1 \le i \le k)$$

where V_i represents the vector generated by i-tuple and f_t^i denotes the frequency of t-th i-tuple in a sequence. We set $k = 3$, which yields vectors corresponding to mononucleotide tuples, dinucleotide tuples and trinucleotide tuples. On this basis, each RNA sequence would be depicted as a one-dimensional vector with size $V1 + V2 + V3$.

2. **Zigzag Coding:**

The Z-curve theory is a geometric approach utilized for visualizing genome sequences within three-dimensional space, as introduced by [12] and further elaborated by [13]. By employing the Z-transform technique, the frequencies of nucleotides A, C, G, and U, as well as their various combinations (k-mer), in a sequence or open reading frame, are transformed into a three-dimensional space, as originally proposed by [14]. In our feature engineering process, we embraced the subsequent representation of the Z-curve:

Phase-independent frequency: This encoding of Z-curve is represented by a 48-bit descriptor as follows:

$$\begin{cases} x_{LM} = [(p(LMA) + p(LMG)) - (p(LMC) + p(LMT)] \\ y_{LM} = [(p(LMA) + p(LMC)) - (p(LMG) + p(LMT)] \\ z_{LM} = [(p(LMA) + p(LMT)) - (p(LMC) + p(LMG)] \end{cases}$$

where the normalized frequency of trinucleotides JLM, LMC, LMG, LMT are represented by $p(LMA$, $p(LMC)$, $p(LMG)$, $p(LMT)$, respectively. The dimension of the feature matrix is 48.

Phase-specific frequency: This is succinctly represented utilizing Z-curve parameters, encapsulated within a 144-bit descriptor as outlined below:

$$\begin{cases} x_{LM}^k = [(p^k(LMA) + p^k(LMG)) - (p^k(LMC) + p^k(LMT)] \\ y_{LM}^k = [(p^k(LMA) + p^k(LMC)) - (p^k(LMG) + p^k(LMT)] \\ z_{LM}^k = [(p^k(LMA) + p^k(LMT)) - (p^k(LMC) + p^k(LMG)] \end{cases}$$

where k represents the three positions of nucleotides at potential codons. The normalized frequency of trinucleotides LMA, LMC, LMG, LMT at different positions were represented by $p^k(LMA)$, $p^k(LMC)$, $p^k(LMG)$, $p^k(LMT)$ respectively generating a feature vector of dimension 144.

3. **Positional gapped k-m-tuple pairs (PGKM):** To capture interactions between non-adjacent residues, gapped k-mer feature generation method is often employed to represent the RNA sequence for the classification tasks [15]. However, such a method discards information about the positions of the different functional subsequences (motifs), which have an important role in recording the distinction between the particular functional sequences (e.g. RNAs). To overcome this limitation, we introduce the positional gap k-m-tuple pair (PGKM) as one of the feature descriptors. PGKM contains three parts: k-tuple ($\{Nu \times k\}$), m-tuple ($\{Nu \times m\}$), and gap (G). The feature generation procedure can be characterized as follows:

$$PGKM = \{Nu \times k\}, (Gap), \{Nu \times m\}$$

where $Nu \in \{A, C, G, T\}$, Gap represents the number of nucleotide intervals between tuples, for $Gap = n$, PGKM will calculate the nucleotides between two tuples less than or equal to n, with a lower bound of 1. k denotes the number of nucleotides in the first tuple, and m denotes the number of nucleotides in the second tuple, respectively. Therefore, in general, when $Gap = n$, PGKM can generate $4^k \times 4^m \times n$ features for an RNA sequence. Considering the sequence 'UGCAAC' as an example, PGKM counts the number of times each tuple pair appears in the sequence and uses this number as the value of the corresponding feature, when Gap = 3, k = 1, m = 1, 48 tuple-pairs (features) can be generated, including three cases:

(1) when Gap = 1, the following features are calculated: A_A, A_C, A_G, A_U, C_A, C_C, C_G, C_U, G_A, G_C, G_G, G_U, U_A, U_C, U_G, U_U;

(2) when Gap =2, A_ _A, A_ _ C, A_ _G, A_ _U, C_ _A, C_ _C, C_ _G, C_ _U,

G_ _A, G_ _C, G_ _G, G_ _U, U_ _A, U_ _C, U_ _G, U_ _U are calculated;

(3) when Gap =3, there are 16 features as follows: A_ _ _A, A_ _ _C, A_ _ _G, A_ _ _U, C_ _ _A, C_ _ _C, C_ _ _G, C_ _ _U, G_ _ _A, G_ _ _C, G_ _ _G, G_ _ _U, U_ _ _A, U_ _ _C, U_ _ _G and U_ _ _U. On this basis, the given sequence has: ΣU_C =1, ΣG_A =1, ΣC_A =1, ΣA_C =1, ΣU_ _A =1, ΣG_ _A =1, ΣC_ _C =1, ΣU_ _ _A =1, ΣG_ _ _ C =1. In addition, the value is set to 0 for the remaining features as they do not appear in the sequence 'UGCAAC'.

4. **Guanine Cytosine Quantity (GC_Quantity):** Quantitatively, each Guanine/Cytosine (G/C) base pair was held by three hydrogen bonds, while Adenine/Thymine (A/T) and Adenine/ Uracil (A/U) base pairs are held by two hydrogen bonds. For a long nucleotide sequence, interactions of base stacking had an obvious influence on molecular stability. So, it's easy to understand that nucleic acid with low GC content (the percentage of nitrogenous bases guanine and cytosine on a nucleic acid molecule) was less stable than nucleic acid with high GC content [16], which may be important for the structural integrity and biological activity of RNA molecules. This method is a straightforward way to infer certain biochemical properties of RNA sequences. GC content has been applied for the functional classification of lncRNAs [17] and lncRNA identification and functional annotation based on deep learning [18].

5. **Nucleotides Tilt:** Nucleotide tilt refers to the angular displacement of base pairs from the helical axis in RNA structures. This feature provides information about the spatial orientation of bases within RNA molecules. By analyzing nucleotide tilt, researchers can gain insights into the three-dimensional conformation and potential interactions of RNA molecules. Understanding nucleotide tilt is important for elucidating the structural dynamics of RNA and for predicting how RNA molecules fold and interact with other biomolecules.

6. **Percentage of Bases:** This method calculates the percentage distribution of individual nucleotide bases—Adenine (A), Cytosine (C), Guanine (G), and Uracil (U)—within RNA sequences. It offers a straightforward analysis of the nucleotide composition, which can provide insights into the genetic makeup and potential functional roles of the RNA. By understanding the base composition, researchers can infer evolutionary relationships, identify conserved regions, and predict functional elements within RNA sequences.

$$\text{Base\%} = \frac{\text{Number of specific base}}{\text{Total number of bases}} \times 100$$

7. **Dinucleotide Physicochemical Properties (DPCP):** We utilized 15 DPCPs to analyze and characterize dinucleotide sequences. These properties include: F-roll, F-tilt, F-twist, F-slide, F-shift, F-rise, roll, tilt, twist, slide, shift, rise, energy, enthalpy, and entropy (denoted as PC1-PC15). Following the normalization procedure described by Liu et al [19], the value of each property was scaled to the range [0, 1], which are formulated as below:

$$\text{DPCP}(i) = \text{Norm}(f_i) \cdot \text{PC}(X_i)$$

where X represents one of the 15 physicochemical properties, and i corresponds to one of the 16 possible dinucleotides $\text{Norm}(f_i)$ is the normalized frequency of dinucleotide i, and $\text{PC}(X_i)$ is the value of physicochemical property X for the dinucleotide i. Those properties are then encoded into a 240-dimensional vector.

To mitigate overfitting due to feature redundancy, our server restricts users to selecting a maximum of four physicochemical features for feature generation. The t-SNE visualization of these physicochemical features can be found in Supplementary Figure S15.

Static semantic information

3, 4, 5, 6mer Word2Vec Semantic Information

Word2Vec [20] is a neural network-based method developed by Google for learning continuous vector representations of words. It comes in two flavors: Continuous Bag of Words (CBOW) and Skip-gram. CBOW predicts a word based on its context, while Skip-gram predicts the context words given a target word. In the CBOW model, the distributed representations of context (or surrounding words) are combined to predict the word in the middle. While in the Skip-gram model, the distributed representation of the input word is used to predict the context. In the RNA context, k-mers are treated as words, and sequences of k-mers are treated as sentences. Word2Vec learns to represent each k-mer based on its surrounding k-mers within a defined window size, capturing local context. Word2Vec can effectively capture local contextual relationships between k-mers. The method can also handle large datasets efficiently, making it suitable for large-scale RNA sequence analysis. In this study, word2vec word embedding was used for static characterization of RNA sequences.

3, 4, 5, 6mer Doc2Vec Semantic Information

Doc2Vec [21], an extension of Word2Vec, is designed to learn vector representations for entire documents, in addition to individual words. It introduces the concept of a paragraph vector that captures the semantics of the entire document (or sequence). In this approach, entire RNA sequences are treated as documents, and k-mers within these sequences are treated as words. Doc2Vec learns to represent both the k-mers and the entire sequences in a continuous vector space, capturing the relationships between subsequences and the overall sequence context. By learning embeddings for entire RNA sequences, Doc2Vec captures comprehensive sequence information, including both local and global contexts. The embeddings can be used to measure the similarity between RNA sequences more accurately, aiding in clustering and classification.

3, 4, 5, 6mer FastText Semantic Information

FastText [22] is an extension of the Word2Vec model developed by Facebook AI Research. It improves on Word2Vec by taking into account subword information, which is particularly useful for capturing the semantic meanings of words and subwords in languages with rich morphology. FastText represents each word as a bag of character n-grams, allowing it to generate meaningful vectors even for words not seen during training. In the context of RNA sequence analysis, FastText can be employed to encode subsequences of RNA, specifically k-mers (where k can be 3, 4, 5, or 6). By treating these k-mers as words, FastText captures the semantic relationships between different parts of RNA sequences. The model learns to represent each k-mer based on the characters (nucleotides) it contains and their position within the k-mer, thereby capturing intricate details about the sequence composition.

3, 4, 5, 6mer GloVe Semantic Information

Global Vectors for Word Representation (GloVe) [23] is a word embedding method developed by researchers at Stanford University. Unlike Word2Vec, which relies on local context windows to predict words, GloVe leverages global word-word co-occurrence statistics from a corpus to learn word embeddings. The main idea is to construct a matrix of word co-occurrence counts and factorize it to obtain word vectors. Applying GloVe to RNA involves treating k-mers as words and computing the global co-occurrence statistics of these k-mers within RNA sequences. By factoring this co-occurrence matrix, GloVe generates embeddings that capture the global semantic relationships between different k-mers.

Dynamic semantic information

We evaluated EnrichRBP's performance using k-mer sizes ranging from 3-mers to 6-mers. As shown in Supplementary Figure S16 and Tables 15 and 16, the 3-mer model

achieved the highest AUC on both circRNA (0.951) and linear RNA (0.934) datasets, outperforming the 4-mer, 5-mer, and 6-mer models. Specifically, the 4-mer, 5-mer, and 6-mer models achieved AUCs of 0.938, 0.931, and 0.927 on circRNA, and 0.922, 0.915, and 0.910 on linear RNA. These results suggest that shorter k-mers (3-mers) are more effective in capturing key sequence patterns relevant to RNA-RBP binding. Notably, we observed a performance decline with increasing k mers, consistent with results from previous studies, such as HCRNet [8], which reported similar trends in RNA sequence analysis.

RNA secondary structure information

RNA Partition Function Local Fold (RNAplfold) [24] is designed to predict the local secondary structure of RNA sequences. Unlike traditional RNA folding algorithms that predict the global minimum free energy (MFE) structure for the entire RNA sequence, RNAplfold focuses on predicting local secondary structures within a sliding window along the sequence. This approach allows for the analysis of local structural features, which can be crucial for understanding RNA function and interactions. Firstly, RNAplfold divides the RNA sequence into overlapping windows and computes the secondary structure for each window separately. This method captures the local base-pairing probabilities, providing insights into the stability and structure of small regions within the RNA. Secondly, RNAplfold calculates the partition function for each window, which provides the base-pairing probabilities rather than just the single MFE structure. Finally, the partition function approach accounts for the ensemble of possible structures, offering a more comprehensive view of RNA folding. The output includes probabilities for each possible base pair within the windows, helping to identify regions with strong or weak structural preferences. These probabilities are visualized in a dot plot format, where the intensity of each dot represents the likelihood of the corresponding base pair forming. RNAplfold is used to identify local RNA structural motifs that are critical for biological function, such as stem-loop structures, hairpins, and base pairings. RNAplfold is a powerful tool for analyzing local RNA secondary

structures. By focusing on local regions and computing base-pairing probabilities, it provides detailed insights into RNA structure. Researchers can use RNAplfold to predict protein binding sites for RNAs.

Section S4: Selection of significant feature methods in the Custom Prediction Module

Selection of significant features is a crucial process in machine learning and data mining, aiming to select a subset of relevant features for model construction. It enhances model performance, reduces computational cost, and improves interpretability. Since multi-source biological information yields excessive features, this leads to a laborious training process of the model and also prevents the model from capturing the most critical information that distinguishes the different subsequences. To address these limitations, we propose to use a feature selection module to identify the best subset of features from high-dimensional features. Here, various approaches for feature selection that we integrate can be classified into four broad categories: information-theoretic, similarity-based, statistics-based, and sparse learning-based methods. Information-theoretic methods such as JMI, MIM, and mRMR focus on maximizing relevance and minimizing redundancy. Similarity-based methods like ReliefF and Fisher Score prioritize features that distinguish between classes. Statistics-based methods including CFS, t-Score, and Chi-Square evaluate features based on statistical measures. Sparse learning-based methods like llL21 and lsL21 promote sparsity in feature selection, enhancing model robustness and interpretability. This section provides an overview of these methods, including core formulas and key literature.

Information-Theoretic Methods

1. Joint Mutual Information (JMI)

JMI [25] evaluates the relevance of a feature X_i by considering the mutual information between X_i and the target variable Y, conditioned on the already selected features X_j.

This method addresses redundancy among features.

$$\mathrm{JMI}(X_i) = \sum_{X_j \in S} I\left(X_i; Y | X_j\right)$$

where $I(X_i; Y|X_j)$ is the conditional mutual information between feature X_i and the target variable Y given another feature X_j.

2. Mutual Information Maximization (MIM)

MIM [26] selects features that have the highest mutual information with the target variable, emphasizing individual relevance without considering redundancy.

$$\mathrm{MIM}(X_i) = I(X_i; Y)$$

where $I(X_i; Y)$ denotes the mutual information between feature X_i and the target variable Y.

3. Conditional Infomax Feature Extraction (CIFE)

CIFE [27] balances relevance and redundancy by maximizing mutual information with the target while penalizing redundancy with other selected features. The method maximizes mutual information with the target while penalizing redundancy, regulated by a trade-off parameter λ.

$$\mathrm{CIFE}(X_i) = I(X_i; Y) - \lambda \sum_{X_j \in S} I(X_i; X_j)$$

where $I(X_i; Y)$ is the mutual information between feature X_i and the target variable, $I(X_i; X_j)$ is the mutual information between feature X_i and a previously selected feature X_j, and λ is a parameter that controls the trade-off between relevance and redundancy.

4. Conditional Mutual Information Maximization (CMIM)

CMIM [28] selects features by maximizing the minimum conditional mutual information with the target variable, ensuring each selected feature contributes new

information.

$$\mathrm{CMIM}(X_i) = \min_{X_j \in S} \; I(X_i; Y | X_j)$$

where $I(X_i; Y|X_j)$ represents the conditional mutual information between the feature X_i and the target variable Y, given another feature X_j that is already selected in the subset S. By focusing on maximizing the minimum conditional mutual information, CMIM ensures that each new feature adds distinct and valuable information to the model, enhancing its predictive performance.

5. Double Input Symmetrical Relevance (DISR)

DISR [29] selects features by considering both their individual relevance and their interdependencies with already selected features, normalized by the entropy of the target.

$$\mathrm{DISR}(X_i) = \frac{I(X_i; Y) + \sum_{X_j \in S} I(X_i; X_j)}{H(Y)}$$

where $H(Y)$ is the entropy of the target variable.

where $H(Y)$ represents the entropy of the target variable Y, $I(X_i; Y)$ is the mutual information between feature X_i and the target variable Y, and $I(X_i; X_j)$ is the mutual information between feature X_i and a previously selected feature X_j.

6. Interaction Capping (ICAP)

ICAP [30] focuses on selecting features that provide unique information about the target variable, penalizing redundant interactions with already selected features.

$$\mathrm{ICAP}(X_i) = I(X_i; Y) - \sum_{X_j \in S} [I(X_i; X_j) - I(X_i; X_j | Y)]$$

where $I(X_i; Y)$ represents the mutual information between feature X_i and the target variable Y, and the summation term accounts for the redundancy by considering the mutual information between X_i and previously selected features X_j, adjusted by their conditional mutual information given Y. This approach ensures that each selected feature contributes distinct and valuable information towards predicting the target

variable.

7. Mutual Information Feature Selection (MIFS)

MIFS [31] selects features by maximizing mutual information with the target variable while penalizing redundancy with already selected features, controlled by β.

$$\mathrm{MIFS}(X_i) = I(X_i; Y) - \beta \sum_{X_j \in S} I(X_i; X_j)$$

where β is a parameter controlling redundancy penalty.

8. Minimum Redundancy Maximum Relevance (mRMR)

mRMR [32] selects features that maximize relevance to the target variable while minimizing redundancy among selected features.

$$\mathrm{mRMR}(X_i) = I(X_i; Y) - \frac{1}{|S|} \sum_{X_j \in S} I(X_i; X_j)$$

where $I(X_i; Y)$ is the mutual information between feature X_i and the target variable Y. $|S|$ is the number of features already selected in subset S. $I(X_i; X_j)$ represents the mutual information between feature X_i and feature X_j.

Similarity-Based Methods

1. ReliefF

ReliefF [33] evaluates the importance of features based on their ability to distinguish between instances that are near each other, selecting features that contribute to class separation. The mathematical formulation for ReliefF is:

$$\mathrm{ReliefF}(X_i) = \frac{1}{m} \sum_{j=1}^{m} \left(\frac{1}{k} \sum_{X_k \in \mathrm{NearestHits}(j)} |X_i^j - X_i^k| - \frac{1}{k} \sum_{X_k \in \mathrm{NearestMisses}(j)} |X_i^j - X_i^k| \right)$$

where m is the number of sampled instances, k is the number of nearest neighbors considered, $\mathrm{NearestHits}(j)$ are the k nearest neighbors of the same class as instance j, and $\mathrm{NearestMisses}(j)$ are the k nearest neighbors of a different class. The term

$|X_i^j - X_i^k|$ measures the difference in the value of feature X_i between instances j and k. This method favors features that have small differences for instances within the same class (nearest hits) and large differences for instances from different classes (nearest misses), thus contributing to effective class separation.

2. Trace Ratio

Trace Ratio [34] method selects features that maximize the ratio of between-class scatter to within-class scatter, enhancing class separability.

$$\text{Trace Ratio}(S) = \frac{\text{trace}(S_w)}{\text{trace}(S_b)}$$

where S_w and S_b are the within-class and between-class scatter matrices, respectively.

3. Fisher Score

Fisher Score [35] evaluates features based on their ability to distinguish between different classes, selecting those that maximize between-class variance while minimizing within-class variance.

$$\text{FisherScore}(X_i) = \frac{\sum_{k=1}^{c} n_k (\mu_i^k - \mu_i)^2}{\sum_{k=1}^{c} n_k \sigma_i^k}$$

where n_k is the number of instances in class k, μ_i^k and σ_i^k are the mean and variance of feature X_i in class k.

Statistics-Based Methods

1. Correlation-based Feature Selection (CFS)

CFS [36] selects feature subsets that are highly correlated with the target variable while having low inter-correlation, promoting features that contribute unique information.

$$\text{CFS}(S) = \frac{k \cdot \bar{r}_{cf}}{\sqrt{k + k(k-1)\bar{r}_{ff}}}$$

where k is the number of features, $\bar{r}_{cf}$ is the average feature-class correlation, and $\bar{r}_{ff}$ is the average feature-feature correlation.

2. t-Score

t-Score [37] evaluates features based on the difference in means between two classes, normalized by the variance within each class, selecting features that show significant class separation.

$$\mathrm{tScore}(X_i) = \frac{\left|\mu_i^1 - \mu_i^2\right|}{\sqrt{\frac{(\sigma_i^1)^2}{n_1} + \frac{(\sigma_i^2)^2}{n_2}}}$$

where μ_i^1 and μ_i^2 are the means of feature X_i in classes 1 and 2, respectively. σ_i^1 and σ_i^2 are the variances of feature X_i in classes 1 and 2, respectively. n_1 and n_2 are the number of samples in classes 1 and 2, respectively.

3. F-Score

F-Score [38] generalizes the t-Score to multi-class problems, evaluating features based on their ability to distinguish between multiple classes.

$$\mathrm{FScore}(X_i) = \frac{\sum_{k=1}^{c} n_k(\mu_i^k - \mu_i)^2}{\sum_{k=1}^{c} (n_k - 1)\sigma_i^k}$$

where c is the number of classes. n_k is the number of samples in class k. μ_i^k is the mean of feature X_i in class k. μ_i is the overall mean of feature X_i across all classes. σ_i^k is the variance of feature X_i in class k.

4. Chi-Square (χ^2) Test

The Chi-Square test [39] evaluates the independence of features and the target variable, selecting features that show significant dependence.

$$\chi^2(X_i) = \sum_{k=1}^{c} \frac{(O_k - E_k)^2}{E_k}$$

where O_k and E_k are the observed and expected frequencies of feature X_i in class

k.

5. Gini Index

In building a decision tree, we need to select a feature and a cut point to split the dataset. To find the optimal split point, we can use the Gini index [40]. Gini index is used to measure the weighted Gini impurity obtained after selecting features for segmentation. Higher Gini impurity indicates greater impurity or disorder in the data. The Gini Index measures the impurity of a feature with respect to the target variable, selecting features that provide better class separation.

$$\text{GiniIndex}(X_i) = 1 - \sum_{k=1}^{c} p_k^2$$

where p_k is the probability of class k given feature X_i.

Sparse Learning-Based Methods

1. ℓ1/ℓ2,1-Norm Minimization (llL21)

ℓ1/ℓ2,1-Norm Minimization [41] selects features by promoting sparsity in the weight matrix W, emphasizing features that contribute significantly to the prediction model.

$$\min_{W} \| XW - Y \|_F^2 + \lambda \| W \|_{2,1}$$

where $\| W \|_{2,1} = \sum_{i=1}^{d} \sqrt{\sum_{j=1}^{k} W_{ij}^2}$ is the ℓ2,1-norm of the weight matrix W.

2. Least Squares with ℓ2,1-Norm Minimization (lsL21)

lsL21 [41] is similar to llL21, but it specifically minimizes the least squares loss with an ℓ2,1-norm regularization, promoting feature sparsity and robustness.

$$\min_{W} \| XW - Y \|_2^2 + \lambda \| W \|_{2,1}$$

where $\| W \|_{2,1} = \sum_{i=1}^{d} \sqrt{\sum_{j=1}^{k} W_{ij}^2}$ is the ℓ2,1-norm of the weight matrix W.

Section S5: Sequence prediction methods in the Custom Prediction Module

Traditional machine learning and Advanced deep learning provide powerful tools for analyzing and interpreting complex data, and have a strong contribution to RBP-RNA binding site identification. EnrichRBP integrates major machine learning algorithms, including regression, K-Nearest Neighbor (KNN), decision trees, Gaussian Naive Bayes (GaussianNB), bagging, random forest, AdaBoost, gradient boosting, support vector machines (SVM), Linear Discriminant Analysis (LDA), and extra trees. Additionally, we delve into deep learning methods such as Convolutional Neural Networks (CNN), Recurrent Neural Networks (RNN), Multi-Layer Perceptrons (MLP), and ResNet. The core formulas and explanatory notes of the above algorithm are detailed below.

Traditional Machine Learning Methods

1. Logistic Regression (Regression)

Regression [42] analysis involves identifying the relationship between a dependent variable y and one or more independent variables $x_1, x_2, \ldots, x_p$. The objective is to fit a linear equation to observed data. The coefficients β are estimated to minimize the sum of squared differences between observed and predicted values. Linear regression is foundational in statistics and is often used for prediction and forecasting.

$$y = \beta_0 + \beta_1 x_1 + \beta_2 x_2 + \cdots + \beta_p x_p + \epsilon$$

where ϵ denotes the error term, is foundational in statistics and is extensively utilized for prediction and forecasting. This methodology is particularly valuable in bioinformatics, where it can be applied to understand the influence of multiple biological variables on an outcome of interest.

2. K-Nearest Neighbor (KNN)

KNN [43] is a widely utilized non-parametric method for classification and regression tasks. For a given data point, it identifies the k nearest neighbors in the feature space and predicts the output based on the majority class (for classification) or the average (for regression) of the neighbors' outputs. Due to its simplicity and effectiveness, KNN is a popular choice in data mining. The prediction formula for KNN regression can be expressed as:

$$\hat{y} = \frac{1}{k} \sum_{i \in \mathcal{N}_k(x)} y_i$$

where $\hat{y}$ is the predicted output, k is the number of nearest neighbors, $\mathcal{N}_k(x)$ represents the set of the k nearest neighbors of the data point x, and y_i denotes the output of the i-th neighbor.

3. Decision Tree

Recursive splitting based on metrics such as Gini impurity:

$$\text{Gini impurity} = 1 - \sum_{i=1}^{c} p_i^2$$

or entropy:

$$\text{Entropy} = -\sum_{i=1}^{c} p_i \log(p_i)$$

is fundamental to the operation of Decision trees. Decision trees [44] partition the data into subsets based on the values of input features, with the process continuing recursively until a stopping criterion is met. The choice of splitting criterion, whether Gini impurity or entropy, influences the formation of these subsets. Decision trees are particularly valuable for classification tasks due to their intuitive structure and ease of interpretation. This makes them an important tool in various fields, including bioinformatics, where they can be used to classify biological data and derive meaningful insights from complex datasets.

4. Gaussian Naive Bayes (GaussianNB)

Gaussian Naive Bayes (GaussianNB) [45] operates under the assumption that features

follow a normal distribution and calculates the posterior probability of each class given the data. This method is effective for classifying RNA sequences based on the likelihood of RBP binding. The posterior probability $P(y|x)$ is proportional to the product of the prior probability $P(y)$ and the likelihoods of the features given the class, as described by:

$$P(y|x) \propto P(y) \prod_{i=1}^{n} P(x_i|y)$$

The likelihood $P(x_i|y)$ for each feature is modeled using a Gaussian (normal) distribution:

$$P(x_i|y) = \frac{1}{\sqrt{2\pi\sigma_y^2}} \exp\left(-\frac{(x_i - \mu_y)^2}{2\sigma_y^2}\right)$$

where μ_y and σ_y^2 are the mean and variance of the feature x_i for class y, respectively. GaussianNB is thus well-suited for applications in bioinformatics, where it can be employed to predict RBP binding sites with high accuracy.

5. Bagging (Bootstrap Aggregating)

Bagging [46] involves training multiple models on different bootstrap samples of the dataset and averaging their predictions to reduce variance and improve stability and accuracy. For RNA-RBP binding site identification, Bagging can significantly improve predictive performance by aggregating results from various models, thus mitigating the effects of overfitting and ensuring more robust predictions. The aggregated prediction $\hat{f}(x)$ is calculated as follows:

$$\hat{f}(x) = \frac{1}{B} \sum_{b=1}^{B} \hat{f}^{(b)}(x)$$

where B represents the number of bootstrap samples, and $\hat{f}^{(b)}(x)$ denotes the prediction from the b-th model. This approach leverages the diversity of the models to achieve a more accurate and stable overall prediction.

6. Random Forest (RF)

A Random Forest [47] is an ensemble method that builds multiple decision trees and merges their results to improve accuracy and control overfitting. Each tree is trained on a random subset of features, contributing to the model's robustness and diversity. This approach leverages the strengths of individual trees while averaging out their weaknesses. The final prediction $\hat{f}(x)$ is obtained by averaging the predictions from all the trees in the forest:

$$\hat{f}(x) = \frac{1}{B}\sum_{b=1}^{B} \hat{f}^{(b)}(x)$$

where B is the number of trees in the forest, and $\hat{f}^{(b)}(x)$ represents the prediction from the b-th tree. Random forests are widely used in various applications, including bioinformatics, due to their high accuracy and resilience against overfitting. This makes them particularly effective for complex tasks such as predicting RNA-RBP binding sites, where they can handle the intricacies of biological data and provide reliable predictions.

7. AdaBoost (Adaptive Boosting)

AdaBoost [48] combines weak classifiers to create a strong classifier by iteratively adjusting the weights of misclassified instances, thereby focusing on hard-to-classify cases in subsequent iterations. This method enhances the performance of simple models like decision trees and is effective in improving prediction accuracy. The final strong classifier $\hat{F}(x)$ is a weighted sum of the weak classifiers:

$$\hat{F}(x) = \sum_{m=1}^{M} \alpha_m h_m(x)$$

where α_m is the weight assigned to the m-th weak classifier $h_m(x)$, and M is the total number of weak classifiers. Each α_m reflects the accuracy of the corresponding weak classifier, giving more influence to more accurate classifiers.

8. Gradient Boosting

Gradient Boosting [49] builds an additive model in a forward stage-wise manner. Each

new model is trained to correct the errors made by the combined previous models, using gradient descent to minimize the loss function. This method is powerful for identifying subtle patterns in RNA sequences that influence RBP binding. The model at the m-th stage, $\hat{F}_m(x)$, is given by:

$$\hat{F}_m(x) = \hat{F}_{m-1}(x) + \nu h_m(x)$$

where $h_m(x)$ is the base learner at stage m and ν is the learning rate, controlling the contribution of each base learner to the overall model.

9. Support Vector Machines (SVM)

SVM [50] constructs a hyperplane or set of hyperplanes in a high-dimensional space to classify data points. The objective is to find the maximum margin between different classes, providing the most significant separation between them. SVM is effective in high-dimensional spaces and is used for both classification and regression tasks. The optimization problem for SVM can be formulated as:

$$\min_{\mathrm{w},b} \frac{1}{2} \| \mathrm{w} \|^2 \text{ subject to } y_i(\mathrm{w} \cdot \mathrm{x}_i + b) \geq 1$$

where w is the weight vector, b is the bias, x_i are the input features, and y_i are the class labels.

10. Linear Discriminant Analysis (LDA)

Linear Discriminant Analysis (LDA) [51] aims to find a linear combination of features that best separates two or more classes. It models the difference between classes by assuming that the data for each class follows a Gaussian distribution with the same covariance matrix. LDA is particularly useful for dimensionality reduction before classification. The decision function for LDA can be expressed as:

$$\delta_k(x) = x^T \Sigma^{-1} \mu_k - \frac{1}{2} \mu_k^T \Sigma^{-1} \mu_k + \log(\pi_k)$$

where Σ is the covariance matrix, μ_k is the mean vector for class k, and π_k is the prior probability of class k.

11. Extra Trees (Extremely Randomized Trees)

Similar to Random Forests, Extra Trees [52] build an ensemble of decision trees. However, they use the entire original sample and randomize the choice of split points to reduce variance further. This additional randomness often leads to better performance in certain datasets. The aggregated prediction $\hat{f}(x)$ for Extra Trees is given by:

$$\hat{f}(x) = \frac{1}{B}\sum_{b=1}^{B} \hat{f}^{(b)}(x)$$

where B is the number of trees in the ensemble, and $\hat{f}^{(b)}(x)$ represents the prediction from the b-th tree. This additional randomness often enhances the model's robustness and accuracy.

Advanced Deep Learning Methods

1. Convolutional Neural Networks (CNN)

CNNs [53] use convolutional layers to detect local patterns. Key components include convolutional layers, pooling layers, and fully connected layers. CNNs are effective in capturing motifs and structural patterns critical for RBP binding. The function of a CNN layer can be expressed as:

$$f(x) = \sigma(\sum_{i=1}^{n} w_i \cdot x_i + b)$$

where σ is the activation function, w_i are the weights, x_i are the input features, and b is the bias.

2. Recurrent Neural Networks (RNN)

RNNs [54] are designed for sequential data, where current inputs depend on previous inputs. They have an internal state that is updated recursively. Variants like Long Short-Term Memory (LSTM) [55] and Gated Recurrent Unit (GRU) [56] address the vanishing gradient problem. RNNs are suitable for modeling dependencies in RNA sequences for RBP binding. The hidden state update in an RNN can be represented as:

$$h_t = \sigma(W_{hh}h_{t-1} + W_{xh}x_t + b_h)$$

where h_t is the hidden state at time t, W_{hh} and W_{xh} are weight matrices, x_t is the input at time t, and b_h is the bias.

3. Multi-Layer Perceptrons (MLP)

MLPs [57] are the simplest form of neural networks with multiple layers of perceptrons (neurons) fully connected. Each neuron applies a linear transformation followed by a non-linear activation function. MLPs are used for tasks where data is not spatially and to predict RNA-RBP interactions based on sequence features. The output of an MLP layer can be expressed as:

$$f(x) = \sigma(W \cdot x + b)$$

where W is the weight matrix, x is the input vector, b is the bias, and σ is the activation function.

4. ResNet (Residual Networks)

ResNet [58] introduces residual learning by adding shortcut connections that bypass one or more layers. This approach helps in training very deep networks by addressing the vanishing gradient problem. Residual blocks allow gradients to flow directly through the identity mapping, making deep networks easier to train. ResNet has achieved state-of-the-art performance in various bioinformatics tasks, particularly in those requiring deep network architectures. The residual block can be represented as:

$$y = F(x, \{W_i\}) + x$$

where $F(x, \{W_i\})$ is the residual function representing the layers to be learned, and x is the input to the block.

Section S6: Visualization analysis in the Prediction Modules

In the Non-Custom Prediction Module

To provide users with intuitive (i) data statistics, (ii) result visualization and (iii) biological interpretability in the results section, we designed and illustrate multiple

visual presentation formats.

For data statistics, EnrichRBP employs a dynamic interactive histogram to analyze the quantity of AGCUs in user-input sequences.

For results visualization, EnrichRBP presents calculated probabilities that determine the likelihood of each input sequence acting as an RNA-binding protein (RBP) binding site. Additionally, to illustrate the variation in algorithm performance, EnrichRBP also displays a kernel density plot showing the prediction confidence levels.

For biological interpretability, EnrichRBP offers a sequence motif analysis for both circRNA and linear RNA, allowing users to discern conservation across sequence positions and to identify binding site patterns. Additionally, EnrichRBP includes a saliency map for RNA-RBP binding across different cell lines, depicted as follows: the top bar illustrates potential binding motifs, the middle bar displays a heatmap of sequence attention scores, and the bottom bar represents the user-input sequence. This comprehensive analysis allows users to detect patterns in binding sites and understand the model feature weights, thereby exploring the biological implications of their predictions.

By integrating these components, EnrichRBP not only offers predictive insights into the probability of RBP interactions but also enables users to deepen their understanding of the mechanisms underlying binding site identification.

In the custom prediction module

To help users to visualize and better understand the model's underlying features, EnrichRBP combines the aforementioned predictions with intermediate data analysis. The server creates and produces multiple visualization charts, providing users with an easily understandable and thorough overview of the research findings. The components of the visualization analysis include (i) data statistics, (ii) training process analysis, (iii) results analysis, and (iv) feature analysis, as shown in Table S11. Details of each component are as follows:

For data analysis, EnrichRBP integrates interactive charts that can count all the

sequences entered by the user in the form of pie charts or histograms, from which it is possible to observe the proportion and number of each base in the RNA sequence data set.

To elucidate the training process, EnrichRBP incorporates the following key plots. Firstly, the learning curve plot illustrates the convergence and performance of the model at each training stage in the five-fold cross-validation by examining the model's training score and test score. Concurrently, the cross-validation score plot provides a comprehensive assessment of the model's generalization by evaluating the model's performance on different subsets of the dataset. Ultimately, EnrichRBP provides a discriminant threshold plot that illustrates how the model adjusts its sensitivity to false reporting factors during the training process, visualizing the adjustment process for finding the optimal F1-score.

For results visualization and analysis, EnrichRBP utilizes a range of interactive performance visualization charts, allowing for detailed comparisons of different models based on the evaluation metrics presented in the fifth step. Specifically, EnrichRBP employs ROC curves to offer a comprehensive view of model performance across various machine-learning models. Additionally, EnrichRBP provides confusion matrix plots that compare each test value's predicted categories with their actual categories, aiding in the understanding of the model's classification accuracy in non-normalized scenarios. Furthermore, EnrichRBP provides category prediction error graphs in the form of stacked bars showing the proportion of predictions for the four categories (TP, TN, FP, FN) in the fitted classification model, providing a quick insight into the classifier's ability to predict the correct category method. An Upset plot is also provided to visually depict the relationships between models based on their overlapping predictions. Moreover, a Kernel Density Plot is presented, illustrating the prediction confidence levels across different models. This graphical representation allows users to examine and analyze the distribution of prediction confidences, providing valuable insights into variations in model performance. Overall, this section enables users to comprehensively compare the prediction performances of different models.

In the model's interpretability component, visualization techniques based on Shapley Additive Explanations (SHAP) [59] offer knowledge into the importance and interplay of features. SHAP beeswarm plots show the contribution of individual features to the prediction of being a binding or non-binding site according to the importance of the feature; while SHAP bar plots succinctly show the feature contributions, which helps to interpret the model outputs; SHAP waterfall plots and SHAP heatmaps illustrate the cumulative impact of features on predictions from the perspective of a single feature and multiple features, respectively; SHAP scatter plots are used to show the interactions between different features; Dependence Scatter Plots reveal the relationship between each feature and its SHAP value, helping to understand the relationship between feature values and model predictions.

In addition, EnrichRBP uses Seaborn [60] to create a feature heatmap that directly represents the relationship between features, thereby identifying how the predictor variables associate with the target variable. Partial dependency plots elucidate the relationship between features and target values, highlighting the impact of individual features on predictions. EnrichRBP employs it to visualize how the top five features influence the predicted values.

In summary, the module provides a comprehensive analysis of the results to enrich the user's understanding of the learning process of the model and its reliability.

Section S7: Model evaluation

To evaluate the performance of the customized methods, we employed commonly used performance metrics such as the area under the receiver operating characteristic curve (AUC), accuracy (ACC), recall, F1-score (F1), the Matthews correlation coefficient (MCC), precision, specificity (SP), and the area under the precision-recall curve (AP). The calculations for these metrics are as follows:

$$ACC = \frac{TP + TN}{TP + TN + FP + FN}$$

$$Recall \quad = \frac{TP}{TP + FN}$$

$$F1 = \frac{2 \times TP}{2 \times TP + FP + FN}$$

$$MCC = \frac{TP \times TN - FP \times FN}{\sqrt{(TP + FP)(TP + FN)(TN + FP)(TN + FN)}}$$

$$Precision \quad = \frac{TP}{TP + FP}$$

$$SP = \frac{TN}{TN + FP}$$

$$AP = \sum_{n} (R_n - R_{n-1}) P_n$$

where TP, TN, FP and FN denote the numbers of true positives, true negatives, false positives, and false negatives, respectively. R_n and P_n represent the recall and precision at the n-th threshold. The Area Under the Receiver Operating Characteristic Curve (AUC) and the Area Under the Precision-Recall Curve (AP) serve as scalar metrics to evaluate the discriminative capability of a model. The Receiver Operating Characteristic (ROC) curve illustrates the trade-off between true positive and false positive rates across varying thresholds, while the PR curve, especially pertinent in unbalanced datasets, depicts the trade-off between precision and recall at different thresholds. Higher values of AUC and AP indicate superior model performance in distinguishing RBP binding sites.

Section S8: Custom prediction module: integrating multiple fusion information for the RNA-RBP binding site identification

We developed a Custom Prediction Module to allow users to autonomously select specialized feature selection algorithms and machine learning techniques tailored to their specific research needs. As an illustration, we investigated the application of EnrichRBP in identifying binding sites of linear RNA to the hnRNPC-2 protein. We integrated diverse biological features, including pseudoKNC, Positional Gapped K-M

Tuple Pairs (PGKM), and Dinucleotide Physicochemical Properties (DPCP), as well as the ReliefF feature selection method to effectively capture relevant feature information. Subsequently, we utilized conventional machine learning algorithms, including Regression, Bagging, Support Vector Machines (SVM), and Extra Trees, to identify RNA-RBP binding sites.

EnrichRBP provides a detailed analysis, enabling users to conduct comprehensive performance comparisons between various machine learning models. As illustrated in Figure S4A, the models were compared based on AUC, accuracy (ACC), recall, F1-score, Matthew's correlation coefficient (MCC), precision, specificity, and average precision (AP) metrics. Among the four evaluated methods, Extra Trees emerged as the superior model, demonstrating robust performance with an AUC of 0.947, ACC of 0.905, recall of 0.689, F1-score of 0.743, MCC of 0.689, precision of 0.808, specificity of 0.959, and AP of 0.847. Detailed Scores for the other models can be found in the Supplementary Table S7.

Regarding the visualization of prediction results, Figure S4B offers a comparative analysis of the four machine learning models, assessing their performance via ROC and PR curves. All models demonstrated an AUC exceeding 0.93, reflecting their robust ability to discriminate between positive and negative categories. Notably, Extra Trees stood out with the highest AP value of 0.847, indicating its superior performance. These visualizations provide a detailed understanding of model performance, emphasizing significant differences in prediction confidence and classification ability across models. This analysis is critical in strategically selecting and tuning models to particular real-world applications.

In terms of visualization of the training process, the learning curve plot (Figure S4C) indicates that the model converges to an accuracy score of approximately 0.88 at a later stage, suggesting that the model has fully converged. Notably, the cross-validation score reflects greater variability on test data compared to training data, suggesting that incorporating additional training data could enhance model performance. Furthermore,

the discriminant threshold plot (Figure S4D) reveals that the model was constantly being fine-tuned to optimize the F1-score and that a specific threshold of 0.39 was identified as the optimal value allowing the model to perform optimally in RNA-RBP binding site prediction.

To validate the credibility of different models, Figure S4E depicts the upset plots for predicted positive and negative samples across the various models, highlighting predictive behavior and consensus. The plots illustrate both unique and shared contributions to the classification task. Figure S4F demonstrates the various confidence levels and densities in classification, aligning with observations from the upset plot (Figure S4E). Additional information on other plots of the platform, such as the prediction error plot and the confusion matrix plot, can be found in figures S4-S8 of the Supplementary Material.

Section S9: Data Availability

As an online platform for RNA-RBP binding site identification and analysis, EnrichRBP is freely available to all users at https://airbp.aibio-lab.com/. All codes used for data analysis and manuscript preparation, alongside a description of necessary steps to reproduce the results, can be found in a GitHub repository accompanying this manuscript: https://github.com/wangyb97/EnrichRBP. In terms of the circRNA sequences, 37 circRNA datasets were extracted from the circRNA interactome database (https://circinteractome.nia.nih.gov/). We also utilized a collection of 740 full-length circRNAs from CRIP . In addition, linear RNA datasets were downloaded from iCount (http://icount.biolab.si/), including 31 experimental CLIP datasets with 19 RBPs involved in splicing. Moreover, we collected 261 RBP binding site datasets from multiple databases, including 172 RBPs constructed in the K562, HepG2, HEK293, HEK293T, HeLa and H9 cell lines. These datasets include 65 CLIP-seq datasets for 61 RBPs from the POSTAR database and 196 eCLIP datasets for 111 RBPs from the ENCODE project. All datasets can be downloaded from our platform at

https://airbp.aibio-lab.com/

Section S10: Venn diagrams illustrate the comparison of circRNA and linear RNA predictions across various methods.

The analysis in Supplementary Figure S19 focuses on AGO family binding site predictions to examine the performance differences of various models on circRNA and linear RNA datasets. In the circRNA dataset, models such as CRIP, iCircRBP-DHN, circSLNN, CSCRSites, and HCRNet exhibit a high degree of overlap in their predicted binding sites for AGO family members. For instance, among 1000 annotated positive binding sites, the predictions for AGO1 show an overlap of 590 sites across the five models, while AGO2 and AGO3 exhibit overlaps of 364 and 616 sites, respectively. These results indicate that current models can generally identify a substantial proportion of AGO binding sites in circRNA.

In contrast, the linear RNA dataset reveals a markedly lower overlap among predictions, highlighting significant variability in model performance. Models such as DeepBind, HCRNet, PrismNet, iCircRBP-DHN, and iDeep display minimal overlap in their predictions for linear RNAs. For instance, among 1000 annotated positive binding sites, the predictions for AGO2 show an overlap of 3 sites, AGO2-M of 1 site, and AGO1234 of 4 sites. Notably, iDeep misclassifies all positive samples in the AGO2 dataset as negatives. This discrepancy reflects sequence context and local structural variations of linear RNA have a greater impact on binding site recognition, thereby posing additional challenges for computational algorithms.

These findings highlight the relative consistency of model predictions for circRNA and the significant variability in predictions for linear RNA. The results further underscore the importance of a unified prediction platform that integrates multiple algorithms to address these challenges. By leveraging the complementary strengths of diverse prediction methods, such a platform can overcome the limitations of individual models,

offering robust and comprehensive support for RBP binding site prediction and functional analysis.

Section S11: Impact of Sequence Variants on RNA Secondary Structure in High-Attention Binding Regions

To investigate the impact of structural variants on high-attention binding regions, we employed the RNAfold tool [61] to depict the secondary structures between pre- and post-mutation. We observed marked folding state changes at specific mutation sites (A to G and U to G in Figure S17A; G to C in Figure S17B). These changes disrupt the natural functional regulatory regions, weakening the ability of RNA to interact with RBPs by altering the stability of specific structural elements. However, as illustrated in Figure S17C and Figure S17D, some mutations did not lead to significant structural changes. This has been attributed to the conservation of crucial RNA regulatory elements during evolution by negative selection pressure, which maintains their structural integrity even in the presence of mutations, thereby preventing drastic alterations in RNA structure.

Section S12: Tables

Table S1. Existing computational models in the non-custom prediction module.

Category	Methods	Code	Year
CircRNA-RBP Binding Prediction	CSCRSites	https://github.com/wangyb97/CSCRSites	2019
	CRIP	https://github.com/kavin525zhang/CRIP	2019
	circSLNN	https://github.com/JuYuqi/circSLNN	2019
	iCircRBP-DHN	https://github.com/houzl3416/iCircRBP-DHN	2021

	HCRNet	https://github.com/yangyn533/HCRNet	2022
Linear RNA-RBP Binding Prediction	DeepBind	https://github.com/jisraeli/DeepBind	2015
	iDeep	https://github.com/xypan1232/iDeep	2017
	PrismNet	https://github.com/kuixu/PrismNet	2021
	iCircRBP-DHN	https://github.com/houzl3416/iCircRBP-DHN	2021
	HCRNet	https://github.com/yangyn533/HCRNet	2022
Dynamic RNA-RBP Binding Prediction in Several Cells	PrismNet	https://github.com/kuixu/PrismNet	2021
	HDRNet	https://github.com/zhuhr213/HDRNet	2023

Table S2. The AUC scores for predicting the interaction of 261 RNA-Binding Proteins (RBPs) with TIA1 transcripts in K562 cells using EnrichRBP.

Model	AUC	Model	AUC	Model	AUC	Model	AUC
AARS_K562	0.8257	EIF4G2_K562	0.9373	LIN28A_HEK293	0.8541	SLBP_K562	0.885
AATF_K562	0.8626	ELAVL1_HEK293	0.839	LIN28B_HEK293	0.8125	SLTM_HepG2	0.8503
ABCF1_K562	0.8367	ELAVL1_Hela	0.9618	LIN28B_HepG2	0.9292	SLTM_K562	0.9244
AGGF1_HepG2	0.8562	EWSR1_HEK293	0.9304	LIN28B_K562	0.9343	SMNDC1_HepG2	0.8529
AGGF1_K562	0.8528	EXOSC5_HepG2	0.8477	LSM11_HepG2	0.8372	SMNDC1_K562	0.8814
AGO_HEK293	0.9415	FAM120A_HepG2	0.8911	LSM11_K562	0.9164	SND1_HepG2	0.8514
AKAP1_HepG2	0.9101	FAM120A_K562	0.9268	METAP2_K562	0.9358	SND1_K562	0.9232
AKAP1_K562	0.8933	FASTKD2_HepG2	0.8426	METTL14_Hela	0.9112	SRRM4_HEK293T	0.8822
AKAP8L_K562	0.836	FASTKD2_K562	0.8818	METTL3_Hela	0.8998	SRSF1_HepG2	0.9333
ALKBH5_HEK293	0.8082	FBL_HEK293	0.8851	MOV10_HEK293	0.9107	SRSF1_K562	0.9347
APOBEC3C_K562	0.9415	FIP1L1_HEK293	0.7765	MTPAP_K562	0.8355	SRSF7_HepG2	0.8899
AQR_HepG2	0.8975	FKBP4_HepG2	0.878	NCBP2_HepG2	0.9651	SRSF7_K562	0.9258
AQR_K562	0.845	FMR1_HEK293	0.7969	NCBP2_K562	0.9178	SRSF9_HepG2	0.9068

ATXN2_HEK293T	0.8768	FMR1_K562	0.9121	NIP7_HepG2	0.7885	SUB1_HepG2	0.9052
AUH_HepG2	0.9016	FTO_HepG2	0.8896	NIPBL_K562	0.8713	SUPV3L1_HepG2	0.786
AUH_K562	0.9352	FTO_K562	0.9094	NKRF_HepG2	0.8738	SUPV3L1_K562	0.8382
BCCIP_HepG2	0.8903	FUS_HEK293	0.9462	NOL12_HepG2	0.8238	TAF15_HEK293	0.9842
BCLAF1_HepG2	0.919	FUS_HEK293T	0.8349	NOLC1_HepG2	0.804	TAF15_HepG2	0.9052
BUD13_HepG2	0.8845	FUS_K562	0.8553	NOLC1_K562	0.8745	TAF15_K562	0.9423
BUD13_K562	0.8693	FXR1_HEK293	0.9418	NONO_K562	0.9236	TARDBP_HEK293T	0.8671
C17ORF85_HEK293	0.7981	FXR1_K562	0.8868	NOP56_HEK293	0.9053	TARDBP_K562	0.9533
C22ORF28_HEK293	0.8669	FXR2_HEK293	0.8673	NOP58_HEK293	0.96	TBRG4_HepG2	0.7497
CAPRIN1_HEK293	0.8345	FXR2_HepG2	0.8178	NPM1_K562	0.9504	TBRG4_K562	0.8125
CDC40_HepG2	0.8155	FXR2_K562	0.9125	NUDT21_HEK293	0.7522	TIA1_Hela	0.9689
CPEB4_K562	0.8451	G3BP1_HepG2	0.8837	PABPC4_K562	0.9584	TIA1_HepG2	0.9605
CPSF1_HEK293	0.8056	GEMIN5_K562	0.9277	PABPN1_HepG2	0.8163	TIA1_K562	0.9306
CPSF2_HEK293	0.9038	GNL3_K562	0.9524	PCBP1_HepG2	0.8646	TIAL1_Hela	0.9341
CPSF3_HEK293	0.8203	GPKOW_K562	0.9021	PCBP1_K562	0.9546	TNRC6A_HEK293	0.8369
CPSF4_HEK293	0.8404	GRWD1_HepG2	0.8976	PCBP2_HepG2	0.9588	TNRC6A_K562	0.9272
CPSF6_HEK293	0.8721	GRWD1_K562	0.9331	PHF6_K562	0.9042	TRA2A_HepG2	0.9328
CPSF6_K562	0.948	GTF2F1_HepG2	0.8837	PPIG_HepG2	0.9161	TRA2A_K562	0.9437
CPSF7_HEK293	0.9333	GTF2F1_K562	0.9466	PRPF4_HepG2	0.8438	TROVE2_K562	0.9486
CSTF2_HEK293	0.9376	HLTF_HepG2	0.7638	PRPF8_HepG2	0.8582	U2AF1_HepG2	0.8924
CSTF2_Hela	0.9483	HLTF_K562	0.9372	PRPF8_K562	0.8742	U2AF1_K562	0.8974
CSTF2T_HEK293	0.8732	HNRNPA1_HEK293T	0.9145	PTBP1_HEK293T	0.9408	U2AF2_Hela	0.9108
CSTF2T_HepG2	0.8995	HNRNPA1_HepG2	0.9307	PTBP1_Hela	0.9323	U2AF2_HepG2	0.8942
CSTF2T_K562	0.9068	HNRNPA1_K562	0.941	PTBP1_HepG2	0.8469	U2AF2_K562	0.8822
DDX21_K562	0.8337	HNRNPC_Hela	0.9431	PTBP1_K562	0.9326	U2AF65_Hela	0.9586
DDX24_K562	0.8442	HNRNPC_HepG2	0.9168	PTBP1PTBP2_Hela	0.8953	UCHL5_HepG2	0.8337
DDX3X_HepG2	0.9542	HNRNPD_HEK293	0.9603	PUM1_K562	0.8963	UCHL5_K562	0.9284
DDX3X_K562	0.9572	HNRNPF_HEK293T	0.9246	PUM2_HEK293	0.9807	UPF1_Hela	0.9775
DDX42_K562	0.9248	HNRNPK_HepG2	0.931	PUM2_K562	0.9478	UPF1_HepG2	0.9218
DDX51_K562	0.8631	HNRNPK_K562	0.9446	PUS1_K562	0.851	UPF1_K562	0.9336
DDX52_HepG2	0.8687	HNRNPM_HepG2	0.8825	QKI_HEK293	0.8416	UTP18_HepG2	0.7938
DDX52_K562	0.8585	HNRNPM_K562	0.9446	RBFOX2_HepG2	0.8762	UTP18_K562	0.8461
DDX55_HepG2	0.915	HNRNPU_Hela	0.8952	RBFOX2_K562	0.8297	UTP3_K562	0.7993
DDX55_K562	0.9328	HNRNPU_HepG2	0.8646	RBM15_HepG2	0.83	WDR3_K562	0.9341
DDX59_HepG2	0.8389	HNRNPU_K562	0.8998	RBM15_K562	0.8536	WDR33_HEK293	0.7493
DDX6_HepG2	0.8161	HNRNPUL1_HepG2	0.9039	RBM22_HepG2	0.7794	WDR43_HepG2	0.8076
DDX6_K562	0.9258	HNRNPUL1_K562	0.9214	RBM22_K562	0.9289	WDR43_K562	0.8413
DGCR8_HEK293T	0.8987	IGF2BP1_HEK293	0.8922	RBM27_K562	0.9421	WRN_K562	0.8612
DGCR8_HepG2	0.816	IGF2BP1_HepG2	0.9142	RBPMS_HEK293	0.967	WTAP_Hela	0.8867
DGCR8_K562	0.9209	IGF2BP1_K562	0.9478	RPS11_K562	0.8493	XRCC6_K562	0.8667
DHX30_HepG2	0.822	IGF2BP2_HEK293	0.9393	RPS3_HepG2	0.8759	XRN2_HepG2	0.8371
DHX30_K562	0.8907	IGF2BP2_K562	0.9453	RPS3_K562	0.9305	XRN2_K562	0.9028

DKC1_HepG2	0.8178	IGF2BP3_HEK293	0.9319	RTCB_HEK293	0.7689	YBX3_HepG2	0.8695
DROSHA_HepG2	0.8334	IGF2BP3_HepG2	0.8776	SAFB2_K562	0.9359	YBX3_K562	0.9504
DROSHA_K562	0.9286	ILF3_K562	0.9397	SBDS_K562	0.9404	YTHDF2_Hela	0.8331
EFTUD2_HepG2	0.832	KHDRBS1_K562	0.9636	SDAD1_HepG2	0.8194	YWHAG_K562	0.9198
EFTUD2_K562	0.8223	KHSRP_K562	0.9316	SDAD1_K562	0.8806	ZC3H11A_HepG2	0.8883
EIF3D_HepG2	0.8385	LARP4_HepG2	0.9475	SERBP1_K562	0.9052	ZC3H11A_K562	0.8932
EIF3G_K562	0.9417	LARP4_K562	0.9145	SF3A3_HepG2	0.8538	ZC3H7B_HEK293	0.9339
EIF3H_HepG2	0.8189	LARP7_HepG2	0.7995	SF3B1_K562	0.7796	ZNF622_K562	0.9109
EIF4A3_Hela	0.9061	LARP7_K562	0.9465	SF3B4_HepG2	0.8468	ZNF800_HepG2	0.864
eIF4AIII_Hela	0.9209	LIN28A_H9	0.9151	SF3B4_K562	0.847	ZNF800_K562	0.9366
						ZRANB2_K562	0.8739

Table S3. HNRNPH1 transcript ENST00000524180 binding score to TIA1 (genomic context: intron).

Tissue type	RBS position	CLIP-seq tech	PhastCons score	PhyloP score	Score
HepG2	chr5:179620290-179620317,chr5	eCLIP	0.928	2.238	3.253
HepG2	chr5:179620294-179620327,chr5	eCLIP	0.935	2.246	4.991
HepG2	chr5:179620406-179620436,chr5	eCLIP	0.003	-0.505	3.992
K562	chr5:179620480-179620520,chr5	eCLIP	0.051	0.142	8.134
HepG2	chr5:179620484-179620571,chr5	eCLIP	0.038	0.066	19.549
HepG2	chr5:179620517-179620552,chr5	eCLIP	0.005	-0.093	14.894
HepG2	chr5:179620552-179620575,chr5	eCLIP	0.047	0.218	14.484
K562	chr5:179621730-179621802,chr5	eCLIP	0.005	0.025	18.87
K562	chr5:179621743-179621816,chr5	eCLIP	0.006	0.026	27.703
HepG2	chr5:179621768-179621818,chr5	eCLIP	0.028	0.135	17.765
HepG2	chr5:179621769-179621819,chr5	eCLIP	0.031	0.115	13.219
K562	chr5:179621802-179621812,chr5	eCLIP	0.001	0.061	8.498
HepG2	chr5:179622076-179622101,chr5	eCLIP	0.02	0.156	3.248
K562	chr5:179622270-179622308,chr5	eCLIP	0	-0.356	11.63
K562	chr5:179622279-179622304,chr5	eCLIP	0	-0.26	9.505

Table S4. Performance evaluation in terms of average AUCs for EnrichRBP, HCRNet, iCircRBP-DHN, DeepCLIP, CRIP, CSCRSites, CircSLNN and CircRB on the 31 linear RNA datasets.

LinRNA-31	EnrichRBP	HCRNet	iCircRBP-DHN	CRIP	CSCRSites	DeepCLIP	CircSLNN	CircRB
AGO1234	0.856	0.909	0.788	0.737	0.708	0.667	0.662	0.588
AGO2	0.818	0.804	0.8	0.638	0.636	0.624	0.562	0.609
AGO2-M	0.822	0.809	0.736	0.598	0.583	0.573	0.557	0.538
Binding_1	0.945	0.967	0.925	0.862	0.842	0.802	0.795	0.588

Binding_2	0.962	0.959	0.929	0.852	0.828	0.816	0.754	0.676
eIF4AIII_1	0.978	0.97	0.963	0.952	0.937	0.92	0.894	0.769
eIF4AIII_2	0.973	0.973	0.963	0.954	0.944	0.931	0.897	0.775
ELVAL1-1	0.969	0.946	0.939	0.918	0.91	0.889	0.882	0.808
ELVAL1-2	0.973	0.954	0.943	0.926	0.925	0.906	0.898	0.784
ELVAL1-A	0.944	0.938	0.922	0.898	0.876	0.852	0.845	0.762
ELVAL1-M	0.825	0.825	0.695	0.604	0.581	0.579	0.52	0.525
EWSR1	0.956	0.937	0.918	0.912	0.884	0.83	0.851	0.765
FUS	0.965	0.951	0.947	0.941	0.907	0.844	0.905	0.791
mut-FUS	0.983	0.961	0.946	0.939	0.907	0.844	0.907	0.76
IGF2BP1-3	0.881	0.888	0.781	0.693	0.703	0.686	0.597	0.523
hnRNPC-1	0.971	0.965	0.952	0.963	0.936	0.915	0.935	0.863
hnRNPC-2	0.987	0.98	0.974	0.985	0.967	0.942	0.962	0.863
hnRNPL-1	0.883	0.842	0.829	0.748	0.65	0.647	0.67	0.584
hnRNPL-2	0.868	0.8	0.761	0.74	0.636	0.655	0.654	0.583
HnRNPL-L	0.868	0.824	0.779	0.685	0.632	0.63	0.636	0.555
MOV10	0.914	0.919	0.885	0.814	0.803	0.797	0.764	0.588
NSUN2	0.923	0.898	0.832	0.865	0.798	0.769	0.776	0.672
PUM2	0.978	0.977	0.969	0.963	0.959	0.936	0.92	0.814
QKI	0.984	0.971	0.962	0.967	0.956	0.942	0.929	0.818
SFRS1	0.949	0.941	0.912	0.886	0.885	0.853	0.794	0.659
TAF1S	0.985	0.972	0.971	0.963	0.922	0.849	0.925	0.796
TDP-43	0.937	0.948	0.928	0.911	0.913	0.891	0.841	0.762
TIA1	0.959	0.955	0.945	0.93	0.891	0.862	0.894	0.817
TIAL1	0.957	0.928	0.915	0.898	0.864	0.833	0.847	0.804
U2AF65	0.98	0.978	0.971	0.968	0.918	0.849	0.932	0.852
Y2AF65	0.974	0.962	0.951	0.935	0.906	0.851	0.893	0.792
Avg.	0.935	0.924	0.895	0.86	0.833	0.806	0.803	0.719

Table S5. Performance evaluation in terms of average AUCs for EnrichRBP, HCRNet, iCircRBP-DHN, PASSION, CRIP, CSCRSites, CircSLNN and CircRB on the 37 circRNA datasets.

CircRNA-37	EnrichRBP	HCRNet	iCircRBP-DHN	PASSION	CRIP	CSCRSites	CircSLNN	CircRB
AGO1	0.985	0.928	0.898	0.909	0.905	0.851	0.844	0.75
AGO2	0.972	0.864	0.797	0.822	0.811	0.755	0.715	0.624
AGO3	0.893	0.952	0.92	0.909	0.895	0.82	0.86	0.718
ALKBH5	0.998	0.99	0.979	0.752	0.721	0.798	0.583	0.593
AUF1	0.986	0.982	0.985	0.979	0.98	0.94	0.971	0.938
C17ORF85	0.987	0.987	0.987	0.86	0.813	0.815	0.721	0.634
C22ORF28	0.983	0.932	0.913	0.894	0.876	0.878	0.797	0.731
CAPRIN1	0.981	0.911	0.858	0.86	0.843	0.827	0.745	0.685

DGCR8	0.926	0.92	0.906	0.917	0.914	0.87	0.846	0.77
EIF4A3	0.883	0.855	0.799	0.823	0.812	0.82	0.717	0.662
EWSR1	0.975	0.946	0.942	0.938	0.936	0.882	0.906	0.805
FMRP	0.972	0.937	0.892	0.9	0.898	0.89	0.826	0.737
FOX2	0.978	0.966	0.958	0.83	0.815	0.755	0.602	0.535
FUS	0.842	0.885	0.855	0.859	0.858	0.799	0.77	0.697
FXR1	0.997	0.99	0.994	0.959	0.952	0.871	0.942	0.838
FXR2	0.987	0.959	0.939	0.941	0.938	0.868	0.896	0.774
HNRNPC	0.983	0.979	0.977	0.976	0.972	0.973	0.98	0.941
HUR	0.886	0.905	0.867	0.879	0.874	0.85	0.796	0.666
IGF2BP1	0.918	0.891	0.843	0.845	0.843	0.835	0.76	0.679
IGF2BP2	0.894	0.875	0.831	0.827	0.821	0.752	0.74	0.644
IGF2BP3	0.893	0.871	0.816	0.831	0.822	0.743	0.706	0.635
LIN28A	0.924	0.898	0.857	0.875	0.865	0.84	0.777	0.671
LIN28B	0.948	0.913	0.892	0.889	0.882	0.758	0.822	0.731
METTL3	0.923	0.899	0.852	0.878	0.854	0.808	0.772	0.731
MOV10	0.896	0.875	0.838	0.845	0.849	0.778	0.777	0.698
PTB	0.869	0.854	0.822	0.829	0.826	0.692	0.738	0.663
PUM2	0.986	0.979	0.97	0.952	0.953	0.936	0.932	0.854
QKI	0.994	0.983	0.971	0.927	0.921	0.866	0.866	0.807
SFRS1	0.983	0.977	0.964	0.965	0.964	0.963	0.926	0.836
TAF15	0.992	0.992	0.992	0.967	0.965	0.941	0.968	0.883
TDP43	0.965	0.936	0.926	0.934	0.93	0.923	0.896	0.829
TIA1	0.979	0.965	0.961	0.935	0.932	0.915	0.901	0.827
TIAL1	0.967	0.934	0.917	0.906	0.902	0.898	0.871	0.82
TNRC6	0.991	0.976	0.967	0.785	0.741	0.729	0.662	0.55
U2AF65	0.969	0.934	0.926	0.93	0.928	0.911	0.899	0.787
WTAP	0.976	0.969	0.967	0.794	0.793	0.808	0.732	0.621
ZC3H7B	0.898	0.857	0.804	0.804	0.792	0.794	0.697	0.634
Avg.	0.951	0.932	0.908	0.884	0.876	0.842	0.809	0.729

Table S6. Performance evaluation for the prediction of Linear RNA and RNA-Binding Protein (RBP) QKI binding sites.

Methods	AUC	ACC	MCC	Recall	F1-Score	Precision	Specificity	AP
Bert_3mer+CNN	0.95	0.923	0.776	0.897	0.82	0.755	0.93	0.899
Bert_3mer+RNN	0.922	0.921	0.737	0.736	0.783	0.837	0.965	0.824
Bert_3mer+ResNet-1D	0.967	0.943	0.822	0.87	0.857	0.844	0.961	0.911

Table S7. Performance evaluation for the prediction of Linear RNA and RNA-Binding Protein (RBP) hnRNPC-2 binding sites.

Model	AUC	ACC	Recall	F1-Score	Precision	Specificity	AP	MCC
Logistic Regression	0.928	0.884	0.602	0.674	0.765	0.954	0.737	0.611
Bagging	0.903	0.879	0.601	0.666	0.747	0.949	0.72	0.599
SVM	0.918	0.883	0.593	0.669	0.77	0.955	0.765	0.608
Extra Trees	0.947	0.905	0.689	0.743	0.808	0.959	0.847	0.689

* The physicochemical features of RNA were generated using PseudoKNC, PGKM, and DPCP methods. Feature filtering was carried out with ReliefF to refine the features. Subsequently, the performance of the four machine learning methods in predicting the binding sites of linear RNA and hnRNPC-2 was evaluated using the improved features.

Table S8. A total of 28 different characterization methods and dimensions in EnrichRBP.

Feature name	Feature dimensions
3、4、5、6mer RBPBERT semantic information	(number of sequences, number of tokens per sequence, 768)
3、4、5、6mer FastText semantic information	(number of sequences, number of tokens per sequence, 100)
3、4、5、6mer GloVe semantic information	(number of sequences, number of tokens per sequence, 100)
3、4、5、6mer Word2Vec semantic information	(number of sequences, number of tokens per sequence, 100)
3、4、5、6mer Doc2Vec semantic information	(number of sequences, number of tokens per sequence, 100)
Secondary structure information	For the default (number of sequences, W, 5)
pseudoKNC	(number of sequences, 84) for ktuple=3
Zigzag coding	(number of sequences, 1)
Guanine cytosine Quantity	(number of sequences, 1)
Nucleotides tilt	(number of sequences, 1)
Percentage of bases	(number of sequences, 1)
Positional gapped k-m-tuple pairs	(number of sequences, 256) for gapValue=1, kValue=2 and mValue=2
Dinucleotide physicochemical properties	(number of sequences, 101, 11)

Table S9. Summary of feature selection methods for the Custom Prediction Module in EnrichRBP.

<table>
<tr><td rowspan="2">Feature selection methods</td><td rowspan="2">Information-theoretic</td><td>JMI</td></tr>
<tr><td>MIM</td></tr>
</table>

		CIFE
		CMIM
		DISR
		ICAP
		MIFS
		mRMR
	Similarity-based	ReliefF
		Trace Ratio
		Fisher Score
	Statistics-based	CFS
		t-Score
		F-Score
		Chi-Square
		Gini Index
	Sparse learning-based	llL21
		lsL21

Table S10. Summary of model methods for the Custom Prediction Module in EnrichRBP.

Methods Type	Method Name
Machine Learning	Logistic Regression
	K-Nearest Neighbor
	Decision Tree
	GaussianNB
	Bagging
	Random Forest (RF)
	AdaBoost
	Gradient Boosting
	Support vector machine (SVM)
	Linear Discriminant Analysis (LDA)
	Extra Trees
Deep Learning	Convolutional neural network (CNN)
	Recurrent neural network (RNN)
	MultiLayer Perceptron Neural Network (MLP)
	Residual Networks (ResNet)

Table S11. Result visualization analysis in the prediction modules.

Category	Type	Purpose
Data statistic	Pie chart / Histogram	The number and percentage of AGCUs inputted by users
Training processing	Learning curve	The model's performance (accuracy) changes over training iterations
	Cross validation score	Different scores for the 5-fold cross-validation
	Descrimination threshold	Adjusting the precision and recall balance using the threshold
Result analysis	Confusion matrix	Algorithm Performance via TP, TN, FP, FN
	Roc curve	The trade-off between sensitivity and specificity
	Prediction error bar	The Bar graph showing deviation between predicted and actual values
	Upset plot	The overlapping predictions among different models
	Density plot	The prediction confidence by different models
	Performance metrics plot	Various performance metrics of different models
	Precision recall curve	The trade-off between precision and recall in a classification model
	Det curve	The trade-off between FP and FN
	Violin plot / Box plot	Detailed insight into the distribution of model performance
	Bar plot / Point plot	A concise and visually summary of the overall performance
Feature analysis	Partial dependence plot	The relationship between a feature and the predicted outcome
	SHAP bar plot	The SHAP for the top ten important features in a model
	SHAP scatter plot	The interaction between two different features
	Dependence scatter plot	The impact of different features on model predictions using SHAP
	Feature heatmap plot	The correlation or importance of different features in a heatmap format
	SHAP beeswarm plot	The SHAP in a beeswarm distribution to visualize feature importance
	SHAP heatmap plot	The SHAP in a heatmap, providing an overview of feature importance
	SHAP waterfall plot	The contribution of each feature to the

		final prediction
Bio-interpretability	Motif discovery	Conserved sequence patterns discovered by the MEME method
	Saliency map	Each position’s contribution to binding predictions

Table S12. Visualization and analysis of machine learning results in the Custom Prediction Module.

Category	Type	Purpose
Data statistic	pie chart	demonstrate the number and percentage of AGCUs
Training processing analysis	learning curve	Show how a model's performance (accuracy) changes over training iterations or with different amounts of training data
	cross validation score	Evaluate a model's performance by splitting the data into multiple subsets and training/validating the model on different combinations
	descrimination threshold	Determine the threshold for classification in a binary model, impacting the balance between sensitivity and specificity
Result visualization & analysis	confusion matrix	Display the performance of a classification algorithm, showing TP, TN, FP, FN
	roc curve	Illustrate the trade-off between sensitivity and specificity for different thresholds
	prediction error bar	Visualize the accuracy or error of predictions by displaying bars indicating the deviation between predicted and actual values
	upset plot	Depict the overlapping predictions among different models
	density plot	Show the prediction confidence by different models
	performance metrics plot	Showcase various performance metrics of different models
Feature analysis	partial dependence	Illustrate the relationship between a feature and the predicted outcome while keeping other features constant
	SHAP bar plot	Show the Shapley values (a measure of feature importance) for each feature in a model
	SHAP scatter plot	Demonstrate the interaction between two different features
	SHAP	Visualize the interactions between different features

	interactio n	and their combined impact on model predictions using Shapley values
	feature heatmap plot	Represent the correlation or importance of different features in a heatmap format
	SHAP beeswarm plot	Show Shapley values in a beeswarm distribution to visualize feature importance
	SHAP heatmap plot	Visualize Shapley values in a heatmap, providing an overview of feature importance
	SHAP waterfall plot	Illustrate the contribution of each feature to the final prediction

Table S13. Visualization and analysis of deep learning results in the Custom Prediction Module.

Category	Type	Purpose
Data statistic	pie chart	Demonstrate the number and percentage of AGCUs
Result visualization & analysis	roc curve	Illustrate the trade-off between sensitivity and specificity for different thresholds
	precision recall curve	Demonstrate the trade-off between precision and recall for different threshold settings of a classification model
	det curve	Visualize the trade-off between false acceptance rate and false rejection rate
	violin plot	Provide detailed insight into the distribution and central tendency of model performance
	box plot	
	bar plot	Prvide a concise and visually accessible summary of the overall performance
	point plot	
	upset plot	Depict the overlapping predictions among different models
	density plot	Show the prediction confidence by different models
	performanc e metrics plot	Showcase various performance metrics of different models

Table S14. Visualization and analysis of the results in the Non-Custom Prediction Module.

Category	Type	Purpose
Data statistic	histogram	Demonstrate the number of AGCUs
Result	density plot	Show the prediction confidence by different

visualization & analysis		models
	performance metrics plot	Showcase various performance metrics of different models
Biological interpretability	motif discovery	Highlight conserved sequence patterns learned by the model
	saliency map	Quantify each position's contribution to binding predictions, facilitating motif identification

Table S15. AUC performance for different k-mer sizes in the circRNA dataset.

CircRNA-37	3mer	4mer	5mer	6mer
AGO1	0.985	0.981	0.934	0.917
AGO2	0.972	0.965	0.933	0.957
AGO3	0.893	0.889	0.881	0.878
ALKBH5	0.998	0.994	0.986	0.977
AUF1	0.986	0.978	0.974	0.971
C17ORF85	0.987	0.979	0.973	0.932
C22ORF28	0.983	0.979	0.965	0.963
CAPRIN1	0.981	0.977	0.939	0.966
DGCR8	0.926	0.913	0.899	0.911
EIF4A3	0.883	0.879	0.862	0.857
EWSR1	0.975	0.962	0.963	0.944
FMRP	0.972	0.959	0.96	0.947
FOX2	0.978	0.974	0.931	0.963
FUS	0.842	0.8	0.83	0.827
FXR1	0.997	0.959	0.977	0.971
FXR2	0.987	0.972	0.961	0.972
HNRNPC	0.983	0.959	0.971	0.968
HUR	0.886	0.882	0.874	0.871
IGF2BP1	0.918	0.896	0.906	0.889
IGF2BP2	0.894	0.862	0.865	0.872
IGF2BP3	0.893	0.889	0.875	0.87
LIN28A	0.924	0.916	0.912	0.879
LIN28B	0.948	0.944	0.936	0.933
METTL3	0.923	0.891	0.901	0.908
MOV10	0.896	0.881	0.88	0.881
PTB	0.869	0.865	0.835	0.849
PUM2	0.986	0.959	0.95	0.942
QKI	0.994	0.99	0.982	0.97
SFRS1	0.983	0.967	0.971	0.961
TAF15	0.992	0.982	0.978	0.961
TDP43	0.965	0.949	0.953	0.946
TIA1	0.979	0.975	0.967	0.964

TIAL1	0.967	0.963	0.942	0.952
TNRC6	0.991	0.966	0.979	0.976
U2AF65	0.969	0.965	0.957	0.954
WTAP	0.976	0.948	0.963	0.959
ZC3H7B	0.898	0.894	0.886	0.844
Avg.	0.950783784	0.937919	0.931108	0.927081

Table S16. AUC performance for different k-mer sizes in the linear RNA dataset.

linearRNA-31	3mer	4mer	5mer	6mer
AGO1234	0.856	0.853	0.845	0.816
AGO2	0.818	0.812	0.807	0.776
AGO2-M	0.822	0.819	0.79	0.804
Binding_1	0.945	0.942	0.934	0.927
Binding_2	0.962	0.954	0.927	0.942
eIF4AIII_1	0.978	0.97	0.967	0.96
eIF4AIII_2	0.973	0.97	0.923	0.955
ELVAL1-1	0.969	0.966	0.932	0.938
ELVAL1-2	0.973	0.96	0.962	0.955
ELVAL1-A	0.944	0.941	0.933	0.926
ELVAL1-M	0.825	0.813	0.814	0.806
EWSR1	0.956	0.944	0.943	0.938
FUS	0.965	0.962	0.948	0.894
mut-FUS	0.983	0.942	0.943	0.965
IGF2BP1-3	0.881	0.843	0.856	0.863
hnRNPC-1	0.971	0.957	0.951	0.947
hnRNPC-2	0.987	0.964	0.976	0.969
hnRNPL-1	0.883	0.88	0.872	0.825
hnRNPL-2	0.868	0.847	0.822	0.845
HnRNPL-L	0.868	0.837	0.857	0.85
MOV10	0.914	0.911	0.895	0.896
NSUN2	0.923	0.915	0.899	0.894
PUM2	0.978	0.975	0.967	0.944
QKI	0.984	0.952	0.973	0.956
SFRS1	0.949	0.935	0.938	0.931
TAF1S	0.985	0.982	0.957	0.967
TDP-43	0.937	0.911	0.92	0.908
TIA1	0.959	0.956	0.948	0.941
TIAL1	0.957	0.942	0.946	0.939
U2AF65	0.98	0.971	0.96	0.962
Y2AF65	0.974	0.959	0.959	0.942
Avg.	0.934419	0.922097	0.914968	0.909065

Table S17. Overview of databases used in EnrichRBP.

Dataset Type	Number of RBP Binding Site Datasets Within Each Cell Line	Source/Database	Key Features
CircRNA-RBP binding site fragment datasets	37	CircRNA interactome database [62]	CircRNA sequences with associated binding proteins
Linear RNA-RBP binding site datasets	31	iONMF database [63]	Including 19 RBPs involved in splicing and 3'UTR processing
mRNA data across six cell lines:	65	POSTAR database [64]	CLIP-seq data for 61 RBPs
K562, HepG2, HEK293, HEK293T, HeLa, H9	196	ENCODE project [65]	eCLIP data for 111 RBPs

Section S13: Figures

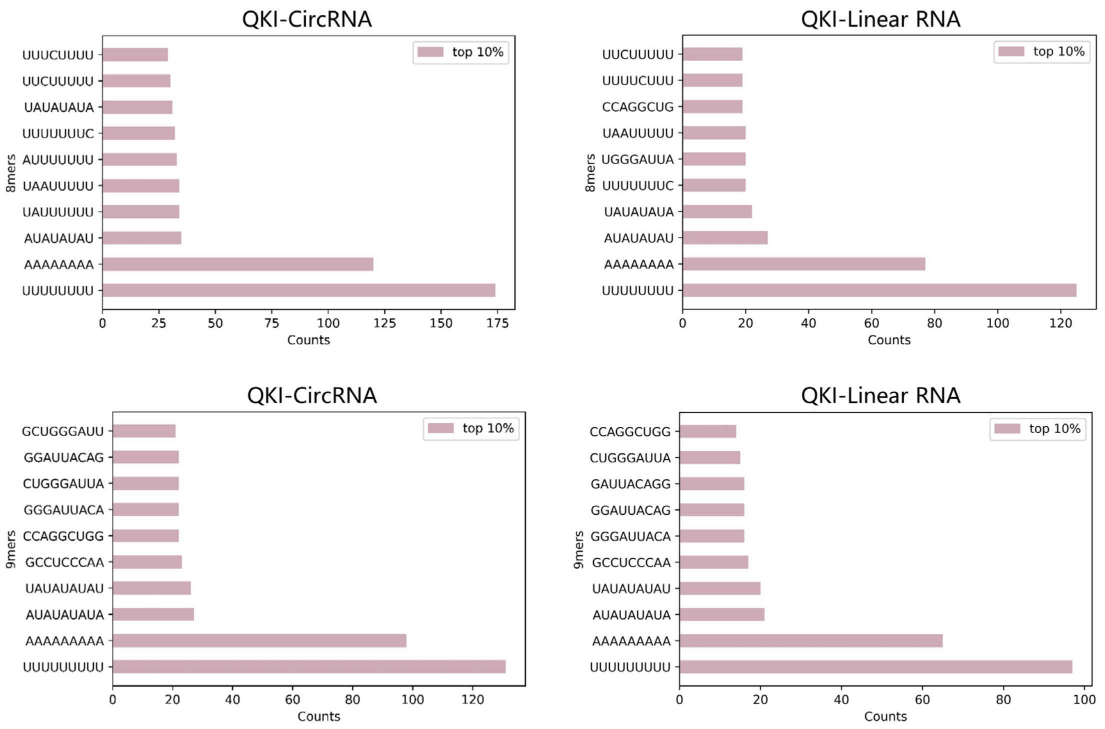

Figure S1. Statistics of the top 10 significant 8-mer and 9-mer regions identified by the MEME method on the QKI dataset.

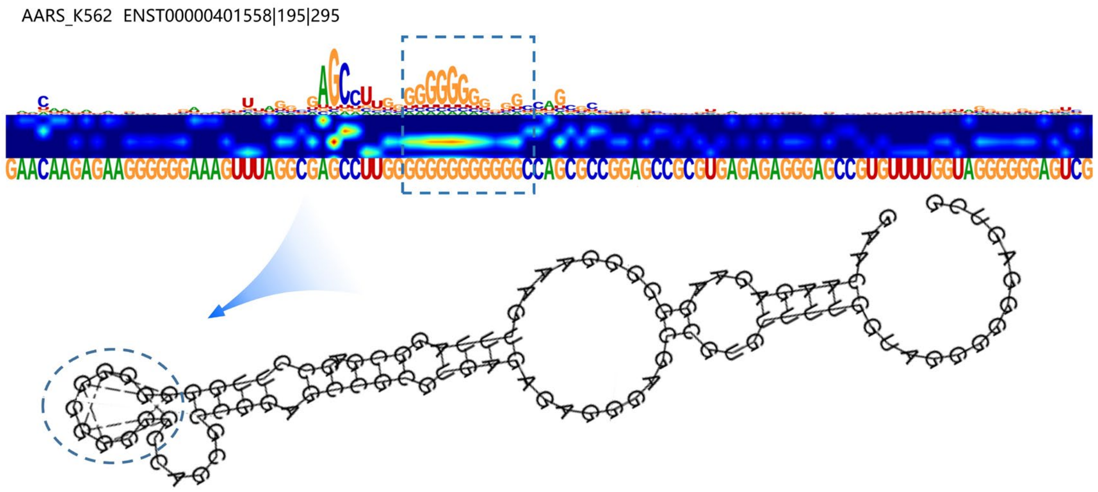

Figure S2. EnrichRBP identifies the salient AARS binding region of G-rich segment with the G-quadruplex structure.

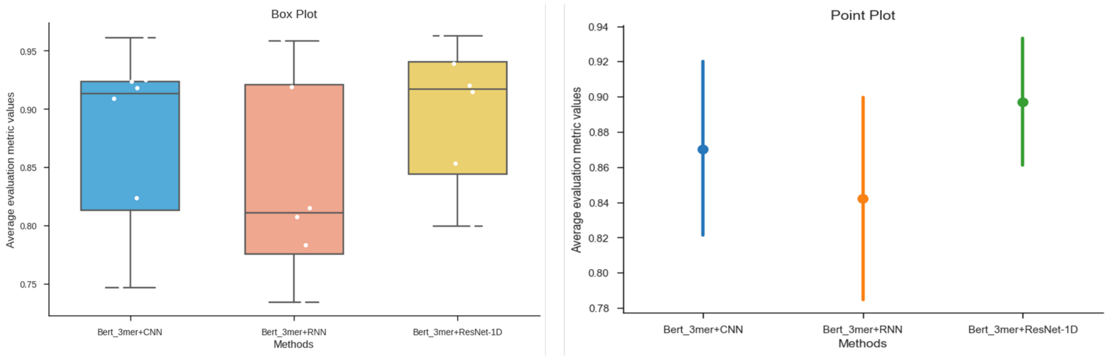

Figure S3. Box plots and point plots of the eight metrics for the different models. This figure combines box plots and point plots to compare the performance of three different models (Bert_3mer+CNN, Bert_3mer+RNN, and Bert_3mer+ResNet-1D) used for RNA-RBP binding site prediction. Eight key performance metrics are illustrated: AUC (Area Under the Curve), ACC (Accuracy), MCC (Matthews Correlation Coefficient), Recall, F1 Score, Precision, Specificity, and AP (Average Precision). Boxes show the quartiles of the dataset, while whisker line extensions show the rest of the distribution, except for points identified as "outliers" using a function of the inter-quartile range. The position of the point indicates the central tendency of the estimated numerical variable (the mean of the eight indicators) and the uncertainty of the estimate is indicated by an error bar.

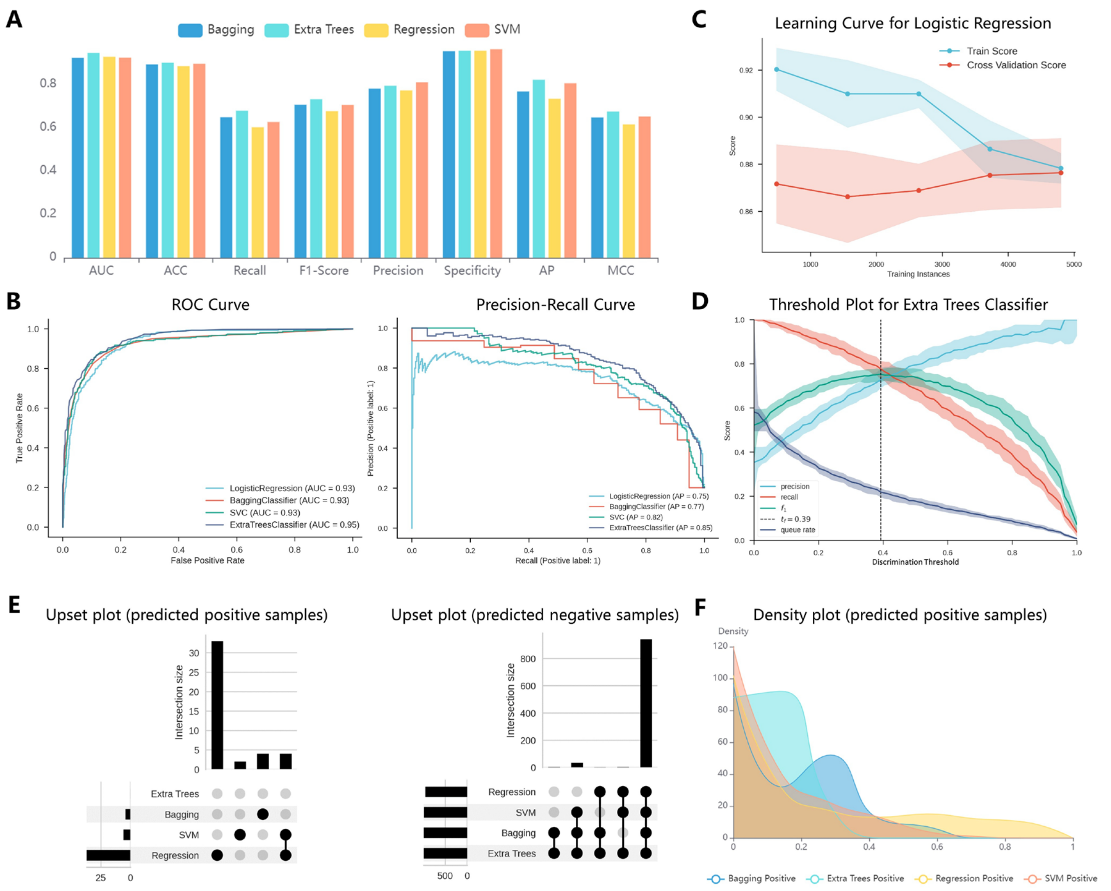

Figure S4. (A) Performance comparison of different machine-learning models using ACC, AUC, Recall, F1-score, Precision, Specificity, AP and MCC. (B) ROC and PR curves of the different models. (C and D) The trends of the learning curve and calculation threshold with each epoch, showing the ACC change and the search for an optimal F1-score process on the specific model. (E) Upset plot to express the relationships of prediction results for the different models. (F) Density distribution of the prediction confidence of different models.

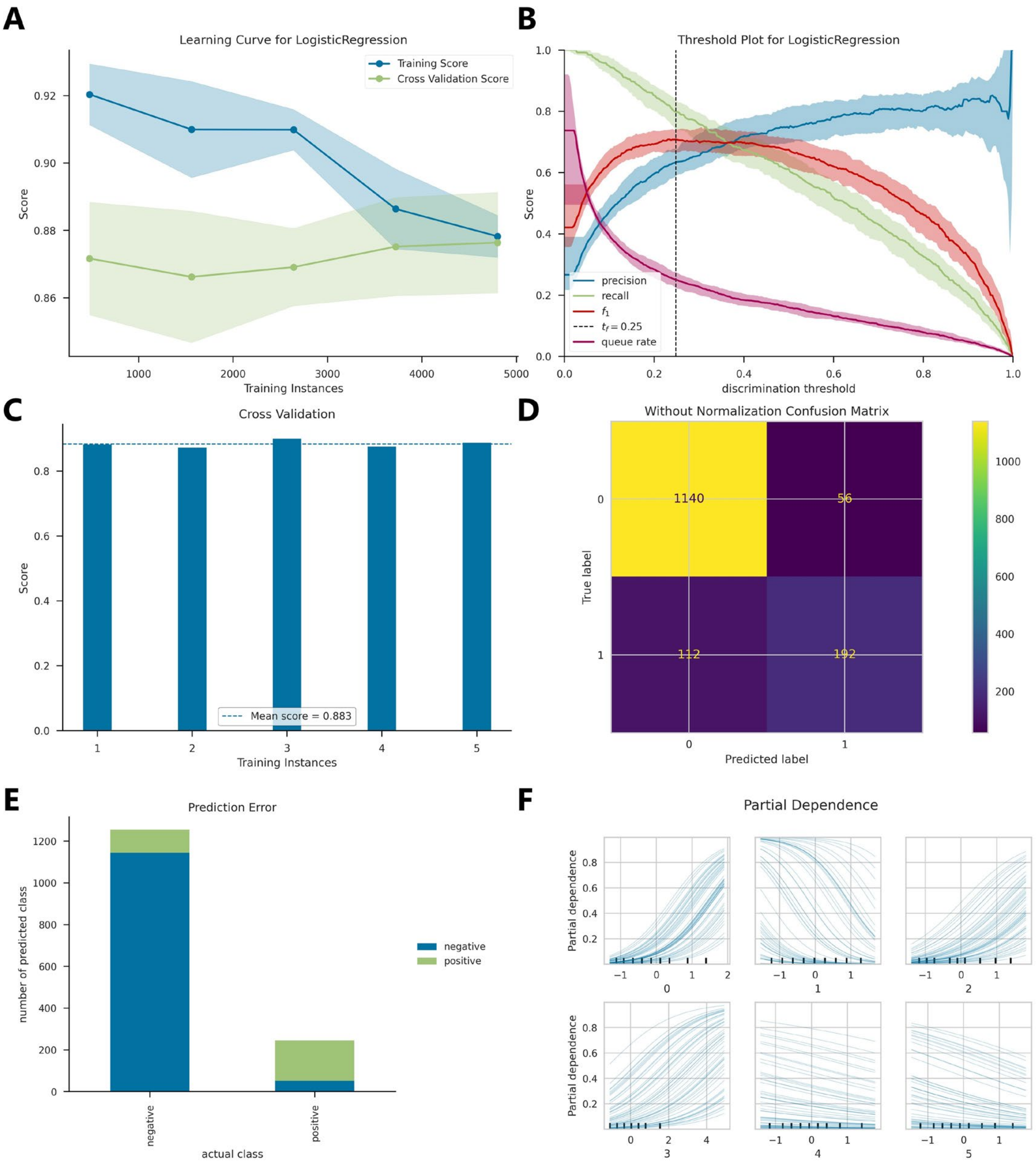

Figure S5. (A) The learning curve plot for the Logistic Regression classifier. With the number of training iterations, the model has fully converged. It is worth noting that the test data reflected in the cross-validation score, i.e. the error bars, shows greater variability than the training data, meaning that additional training data has the potential to improve the performance of the model. (B) The discriminant threshold plot. It shows that the model is constantly being fine-tuned to optimise F1-Score and that a specific threshold of 0.25 is identified as the optimal value to allow the model to perform optimally in RNA-RBP prediction. (C) The Cross-validation Scores (CV scores) plot for the Logistic Regression classifier. The evaluation is based on five-fold cross-

validation with an average performance score of 0.883 for the classifier. The CV scores plot reveals that Logistic Regression has a high average cross-validation accuracy score, but a slight decrease in the performance of the second-fold classifier. (D) Without normalization confusion matrix plot for the Logistic Regression classifier. Each row of the matrix represents the instances in an actual class while each column represents the instances in a predicted class. (E) Class prediction error plot of the Logistic Regression classifier. Both bars are segmented to show the proportion of predictions in each category (including false negatives and false positives, true negatives and true positives). (F) Partial Dependence plot (PDP). The overall trend in PDP for Feature 0 is that the probability of RNA-RBP binding increased as Feature 0 is elevated. A similar analysis is performed for Feature 1 with the opposite trend. The RNA-RBP binding probability decreases as Feature 1 increases. The other features can be analyzed similarly.

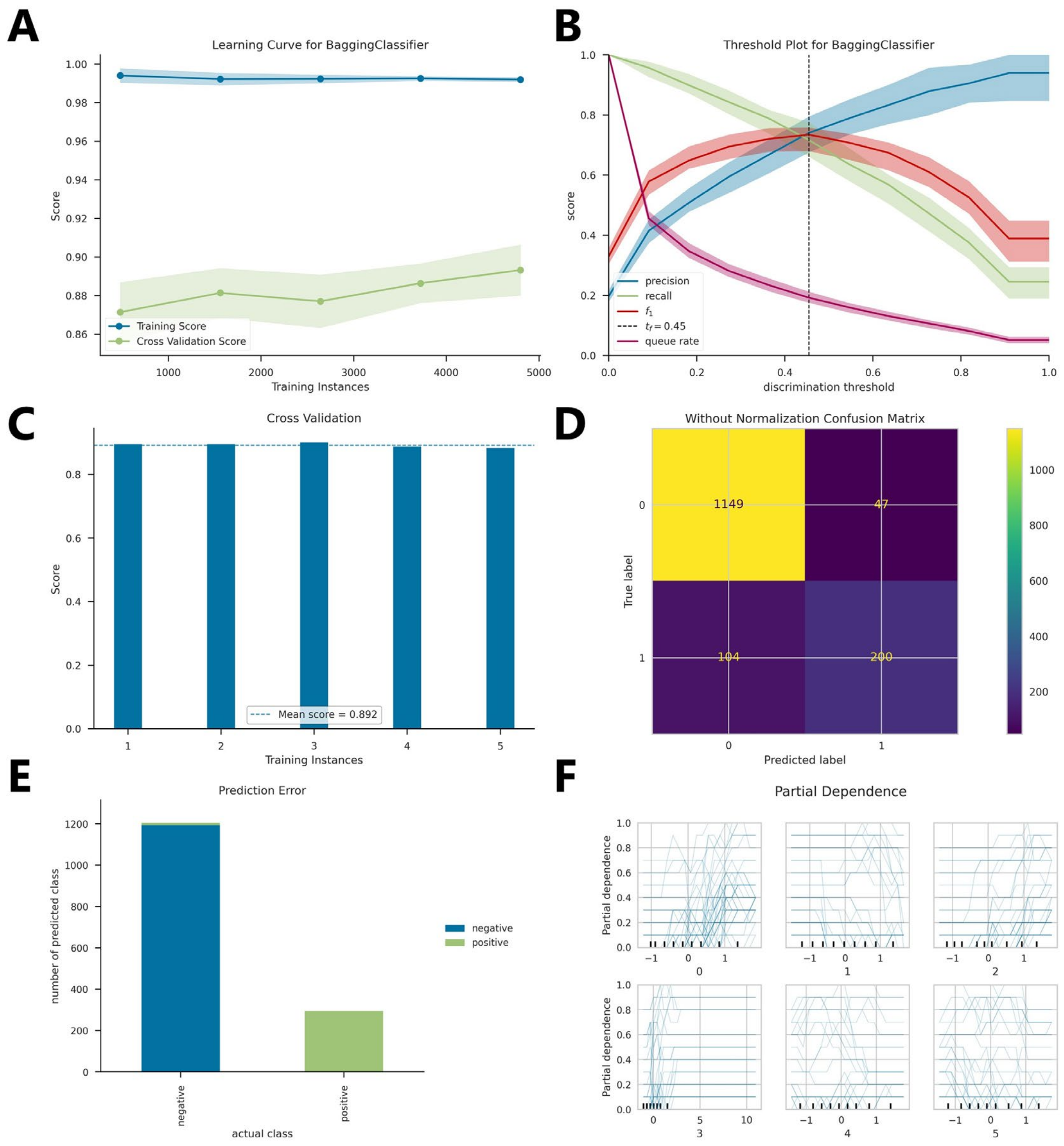

Figure S6. (A) The learning curve plot for the Bagging classifier. The results show that although the model converges to an accuracy of around 0.89 at a later stage, there is still a gap between the training score and the test score, indicating that the model is overfitted. (B) The discriminant threshold plot. It shows that the model is constantly being fine-tuned to optimize F1-Score and that a specific threshold of 0.45 is identified as the optimal value to allow the model to perform optimally in RNA-RBP prediction. (C) The Cross-validation Scores (CV scores) plot for the Bagging classifier. The evaluation is based on five-fold cross-validation with an average performance score of 0.892 for the classifier. The CV scores plot reveals that the Bagging classifier has a high average cross-validation accuracy score, but a slight decrease in the performance of the

fifth-fold classifier. (D) Without normalization confusion matrix plot for the Bagging classifier. Each row of the matrix represents the instances in an actual class while each column represents the instances in a predicted class. (E) Class prediction error plot of the Bagging classifier. Both bars are segmented to show the proportion of predictions in each category (including false negatives and false positives, true negatives and true positives). (F) Partial Dependence plot (PDP). For Feature 0, the partial dependence curve shows variability in the predicted outcome as the feature value ranges from -1 to 1. For Feature 3, the plot has a different x-axis scale (0 to 10), suggesting it might be a categorical or discrete numerical feature. The partial dependence values change significantly across different feature values. The other features can be analyzed similarly.

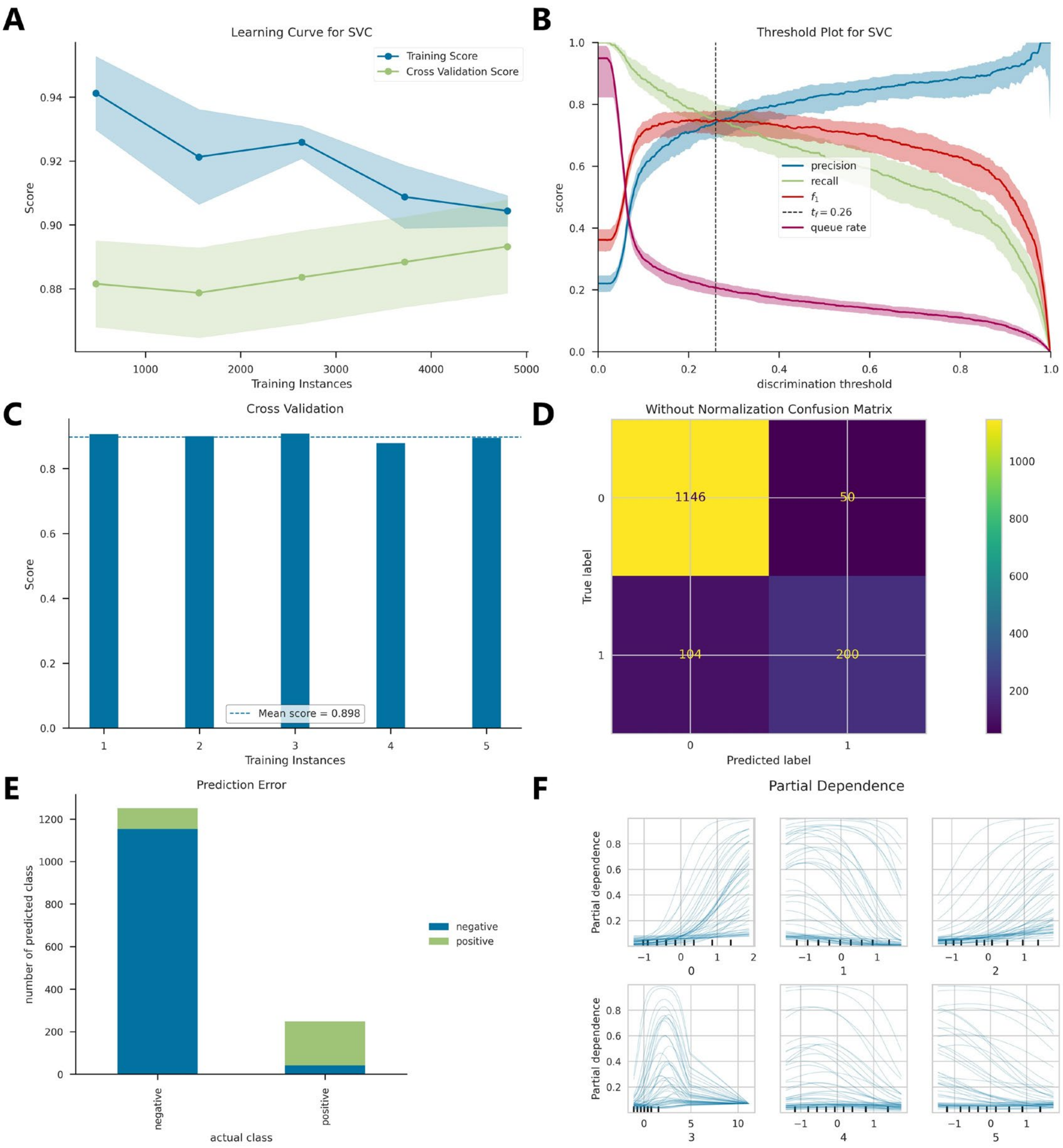

Figure S7. (A) The learning curve plot for the SVM classifier. It shows that although the model converges to an accuracy score of around 0.89 at a later stage, there is still a gap between the training and test scores, suggesting that the model has not yet fully converged. It is worth noting that the test data reflected in the cross-validation score, i.e. the error bars, shows greater variability than the training data, meaning that additional training data has the potential to improve the performance of the model. (B) The discriminant threshold plot the SVM classifier. It shows that the model is constantly being fine-tuned to optimize F1-Score and that a specific threshold of 0.26 is identified as the optimal value to allow the model to perform optimally in RNA-RBP prediction. (C) The Cross-validation Scores (CV scores) plot for the SVM classifier. The evaluation

is based on five-fold cross-validation with an average performance score of 0.898 for the classifier. The CV scores plot reveals that the SVM classifier has a high average cross-validation accuracy score, but a slight decrease in the performance of the forth-fold classifier. (D) Without normalization confusion matrix plot for the SVM classifier. Each row of the matrix represents the instances in an actual class while each column represents the instances in a predicted class. (E) Class prediction error plot of the SVM classifier. Both bars are segmented to show the proportion of predictions in each category (including false negatives and false positives, true negatives and true positives). (F) Partial Dependence plot (PDP). The overall trend in PDP for Feature 0 is that the probability of RNA-RBP binding increased as Feature 0 is elevated. A similar analysis is performed for Feature 1 with the opposite trend. The RNA-RBP binding probability decreases as Feature 1 increases. The other features can be analyzed similarly.

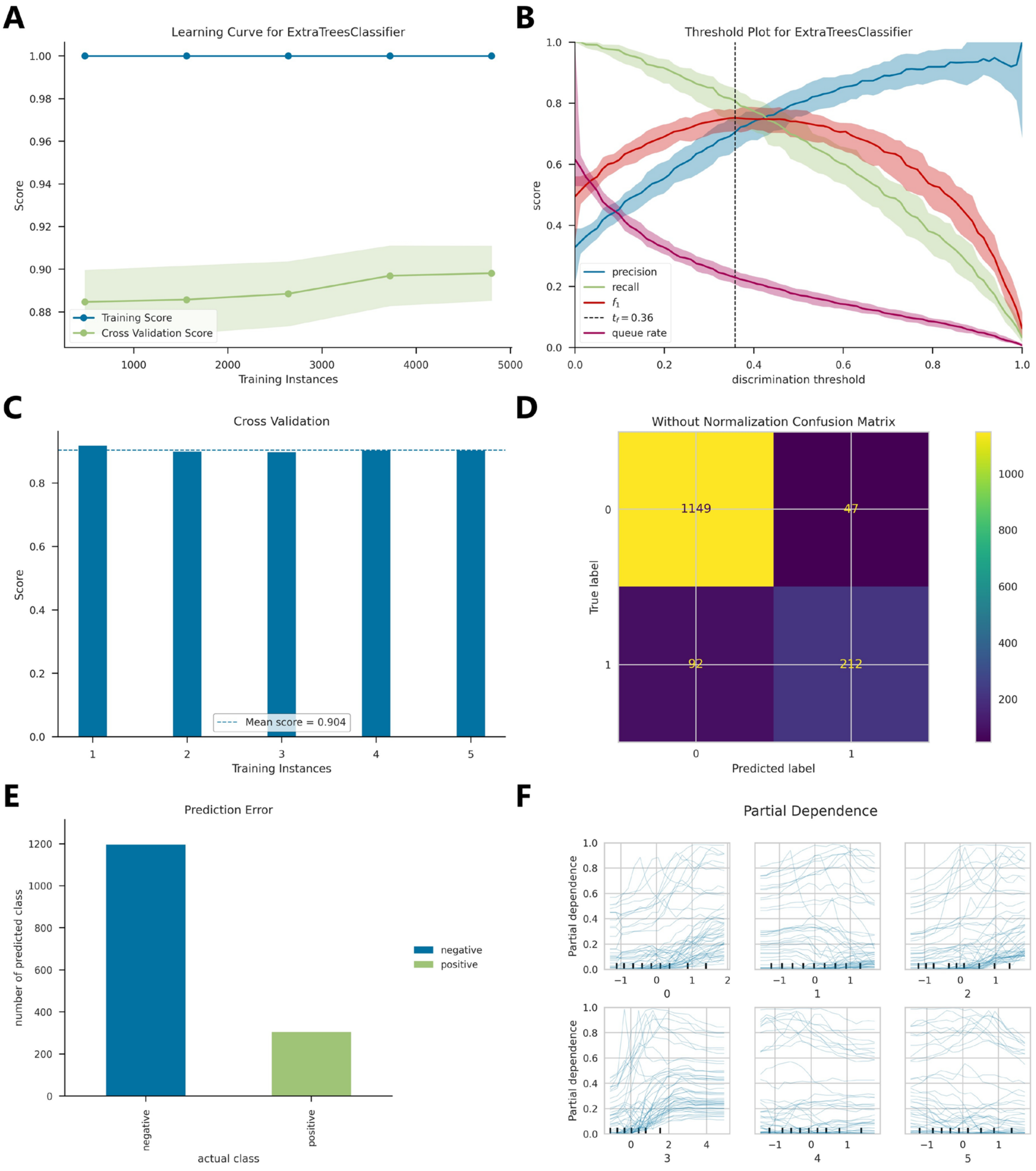

Figure S8. (A) The learning curve plot for the Extra Trees classifier. The results show that although the model converges to an accuracy of around 0.90 at a later stage, there is still a gap between the training score and the test score, indicating that the model is overfitted. (B) The discriminant threshold plot. It shows that the model is constantly being fine-tuned to optimize F1-Score and that a specific threshold of 0.36 is identified as the optimal value to allow the model to perform optimally in RNA-RBP prediction. (C) The Cross-validation Scores (CV scores) plot for the Extra Trees classifier. The evaluation is based on five-fold cross-validation with an average performance score of 0.904 for the classifier. The CV scores plot reveals that the Extra Trees classifier has a

high average cross-validation accuracy score, but a slight decrease in the performance of the third-fold classifier. (D) Without normalization confusion matrix plot for the Extra Trees classifier. Each row of the matrix represents the instances in an actual class while each column represents the instances in a predicted class. (E) Class prediction error plot of the Extra Trees classifier. Both bars are segmented to show the proportion of predictions in each category (including false negatives and false positives, true negatives and true positives). (F) Partial Dependence plot (PDP). For Feature 3, the x-axis for this feature ranges from 0 to 4. The plot shows significant changes in partial dependence values, indicating that Feature 3 has a substantial impact on the model's predictions. For Feature 4, the x-axis ranges from -1 to 1. The curves indicate variability in the predicted outcome as the feature value changes, though the relationship appears less pronounced than in some other feature plots. The other features can be analyzed similarly.

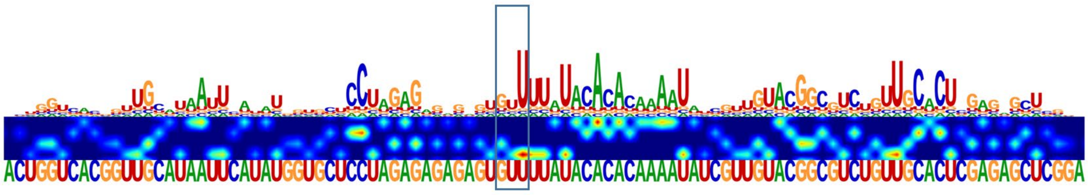

Figure S9. The Saliency map for one significant RBP-propensity binding site, where the top strip plots the potential binding motifs; the second strip is the heatmap of the sequence attention scores; the third strip indicates the specific sequences input by users.

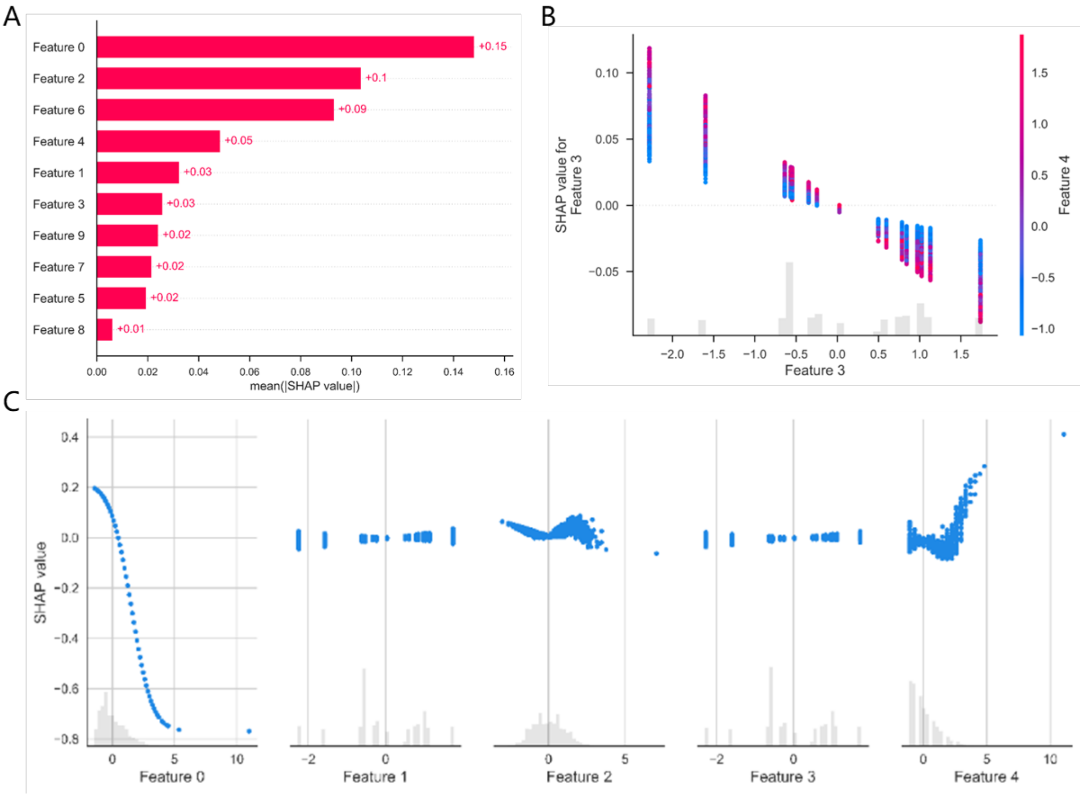

Figure S10. (A) SHAP Bar plot for the Gradient Boosting classifier. The bars illustrate the magnitude of the impact of the different features obtained after filtering by the ReliefF method (user selection) on the model decision process. (B) SHAP Scatter plot for the interaction between feature 3 and the other feature. More than the effect of individual features on the model, the interaction between features is more important, as shown in this figure, where there is a strong correlation between Feature 3 and Feature 4. Notably, as feature 3 increases, larger feature 4 values demonstrate a heightened sensitivity to changes, while smaller feature 4 values show the opposite trend. The light grey area at the bottom of the figure is a histogram showing the distribution of data values. The interactions shown in the figure are a result of the fact that the analyzed features contain global contextual dependencies, and this interaction facilitates the representation of the features, thus further validating the effectiveness of the generated features. (C) Dependence Scatter plot. This figure shows the effect of each of the first five features obtained after filtering by the ReliefF method (user selection) on the predictive effectiveness of the model.

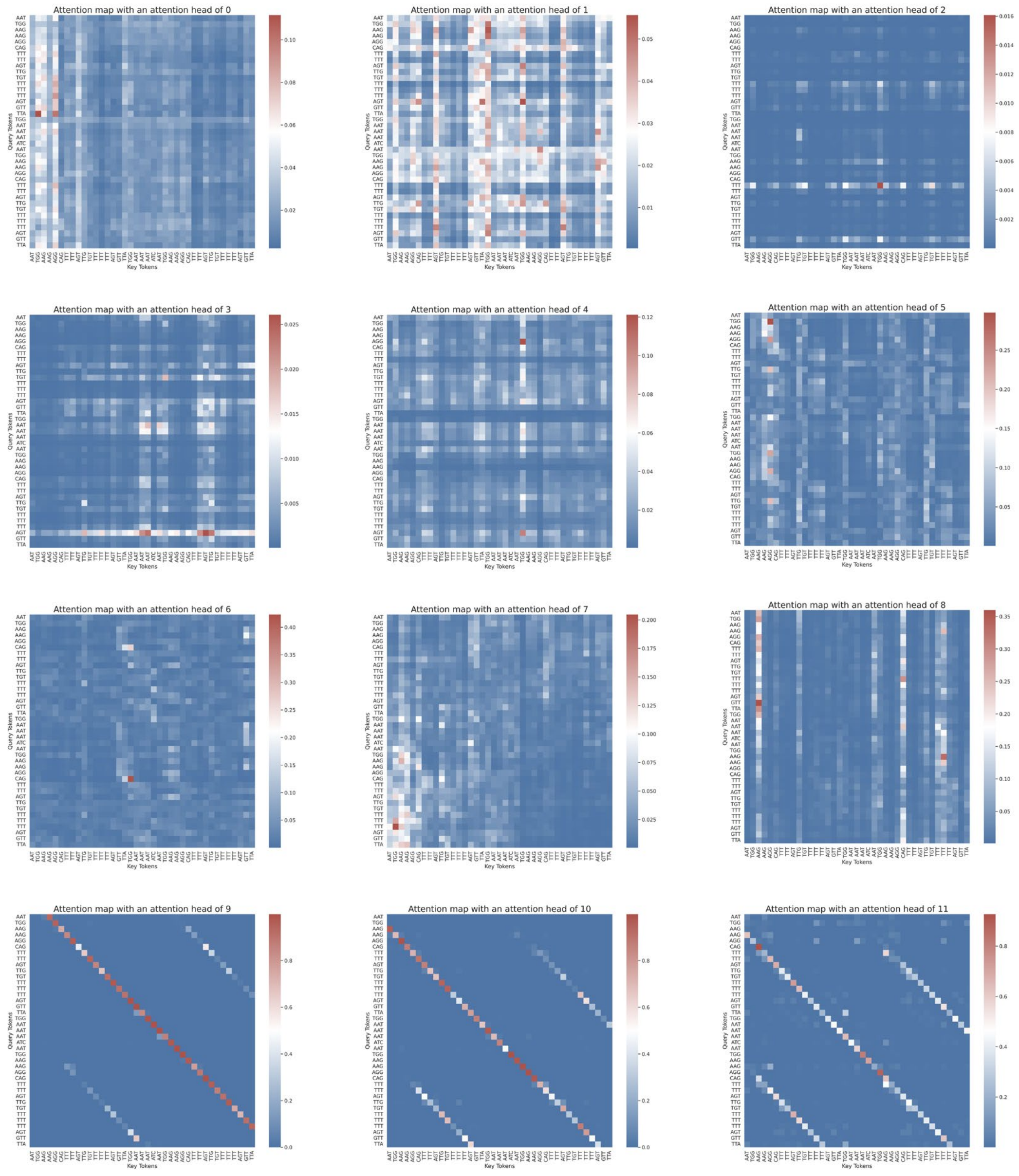

Figure S11. Attention maps for the 0th-11th attention head with a 3-mer split sequence.

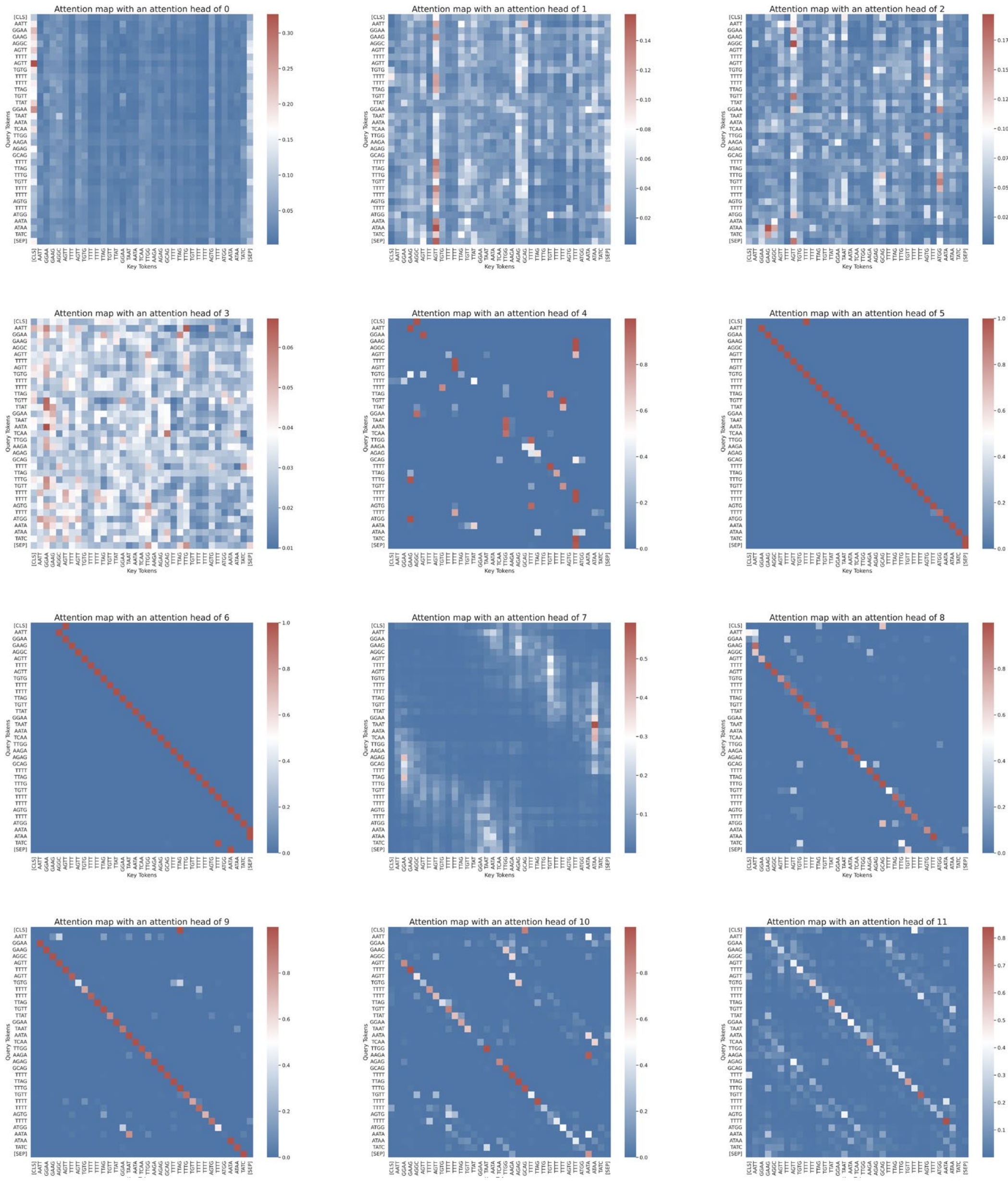

Figure S12. Attention maps for the 0th-11th attention head with a 4-mer split sequence.

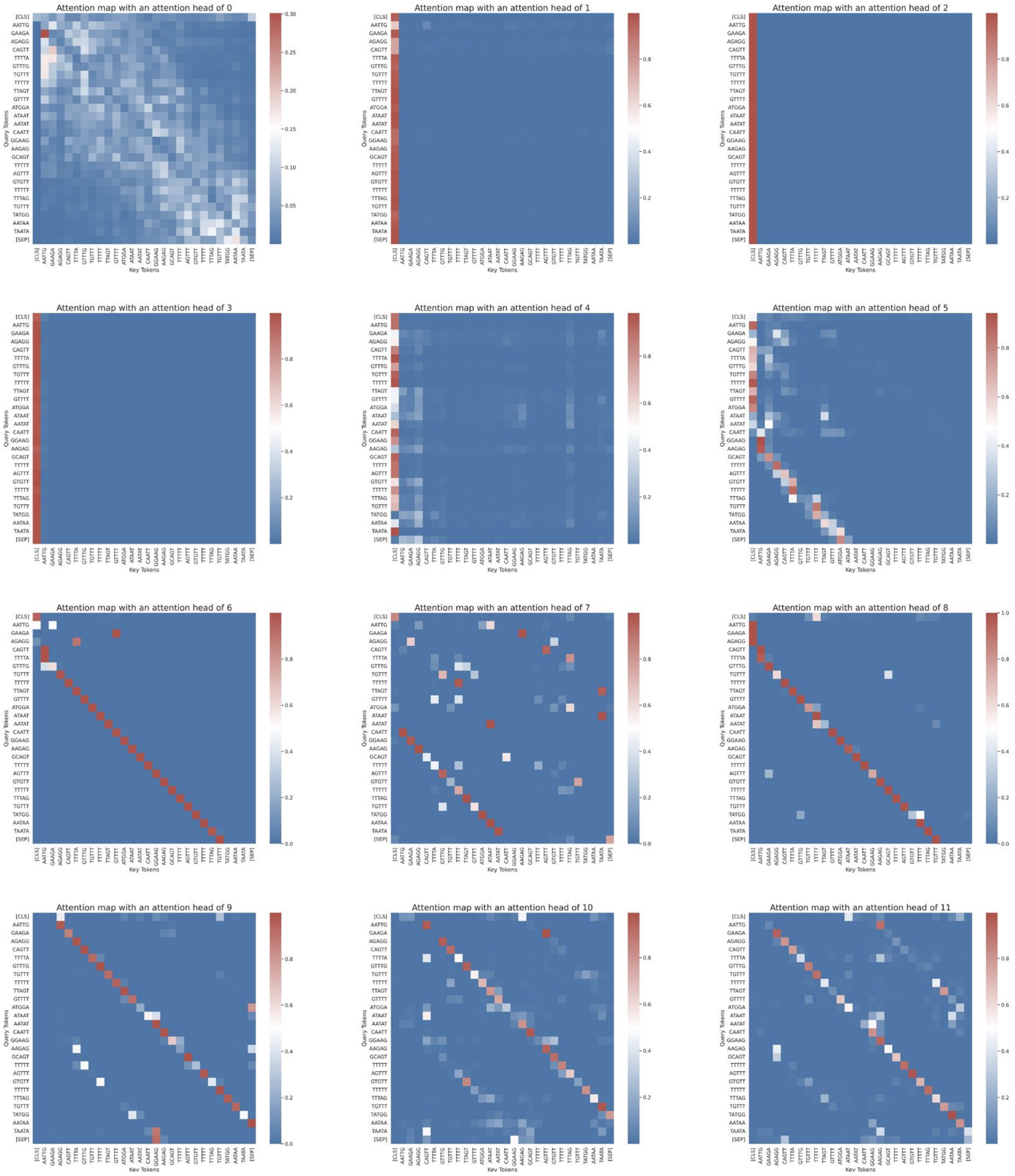

Figure S13. Attention maps for the 0th-11th attention head with a 5-mer split sequence.

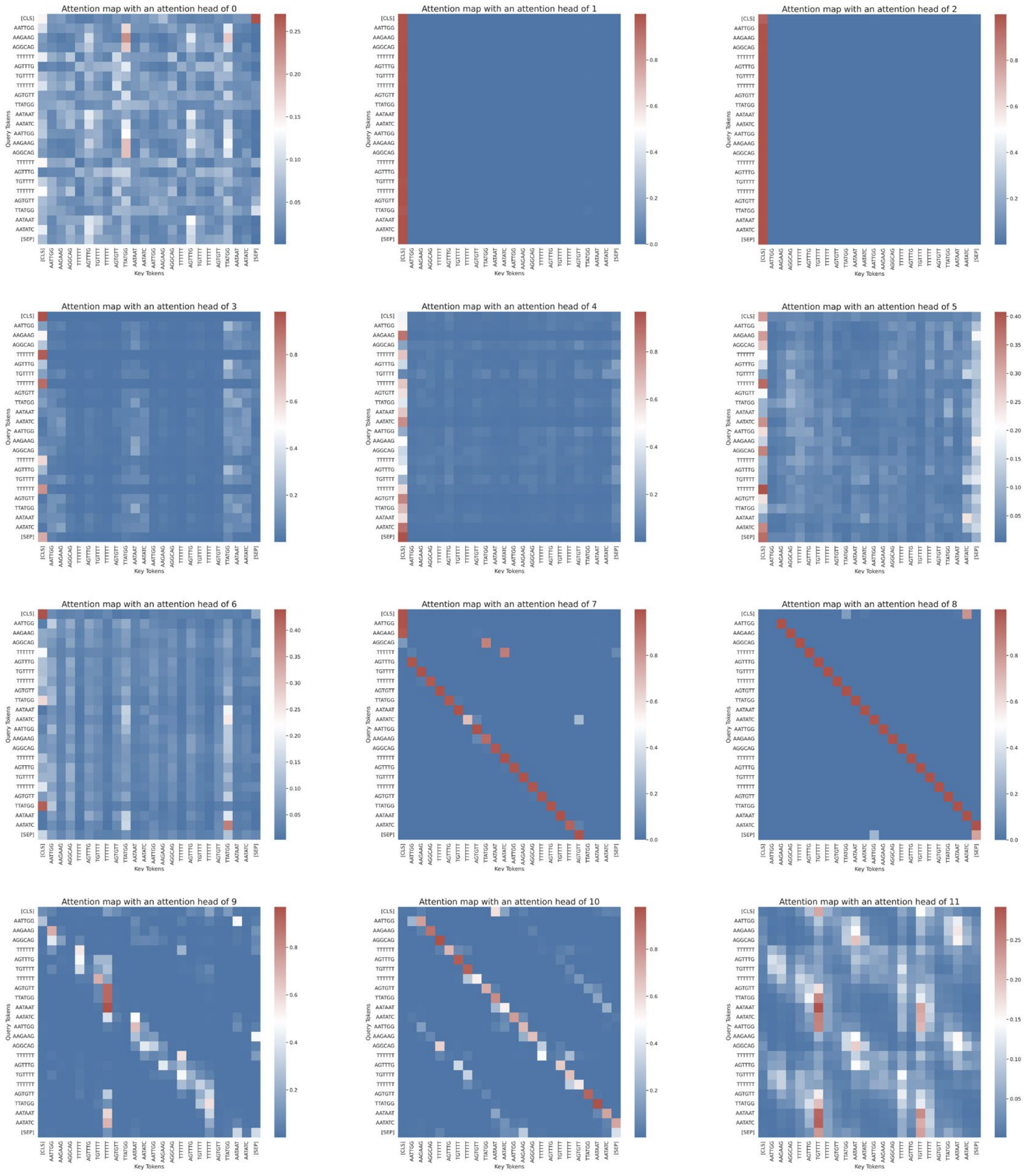

Figure S14. Attention maps for the 0th-11th attention head with a 6-mer split sequence.

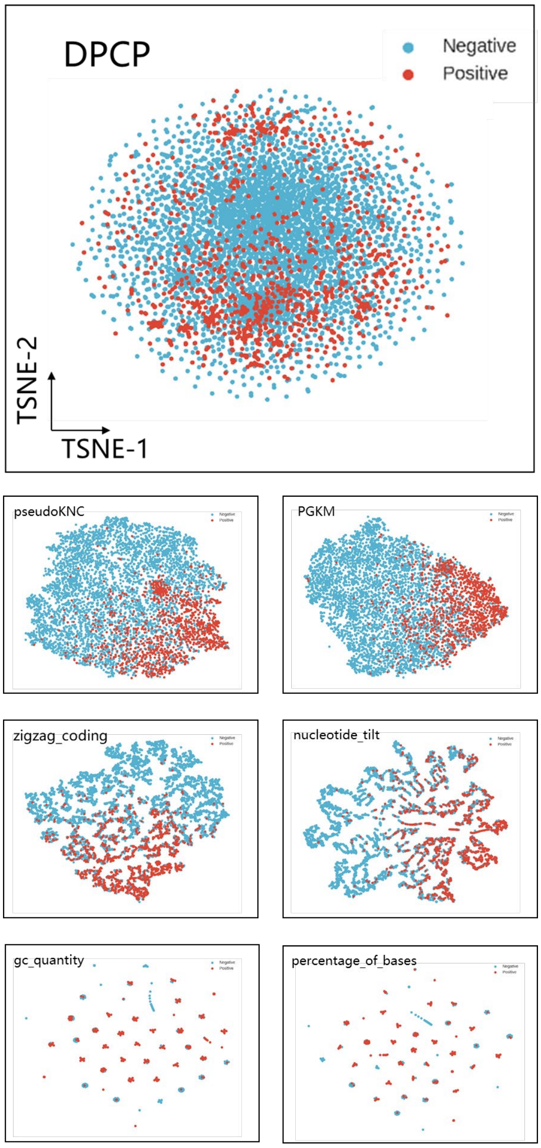

Figure S15. Visualization of t-SNE projections of seven feature extraction methods distinguishing between non-interaction sites (blue) and interaction sites (red): DPCP, pseudoKNC, PGKM, zigzag_coding, nucleotide_tilt, gc_quantity and percentage_of_bases, demonstrating the classification ability of each method in high-dimensional space.

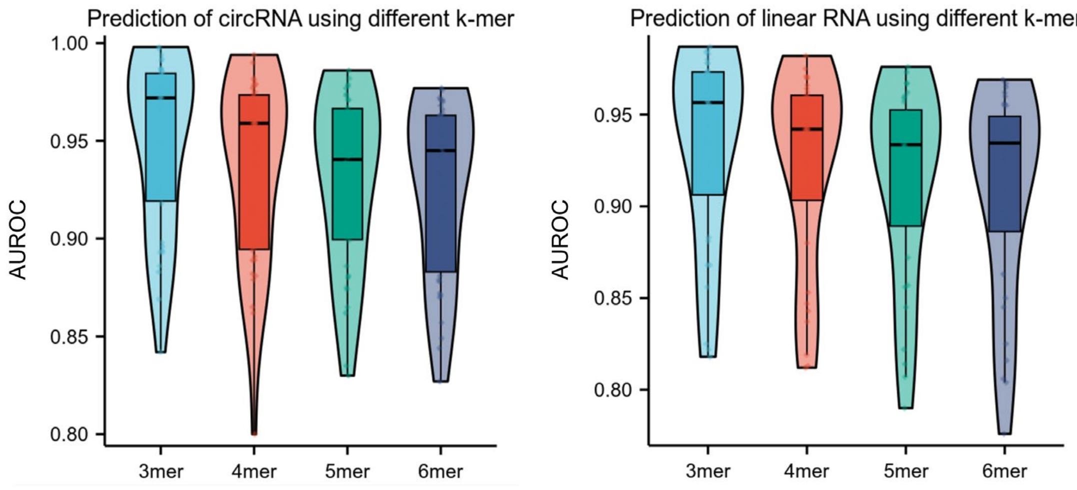

Figure S16. Overall performance comparison of EnrichRBP using different k-mer representations of RBPBERT (n = 37 for circRNA, n = 31 for linear RNA; center line, median; box limits, upper and lower quartiles; whiskers, 1.5 × interquartile range).

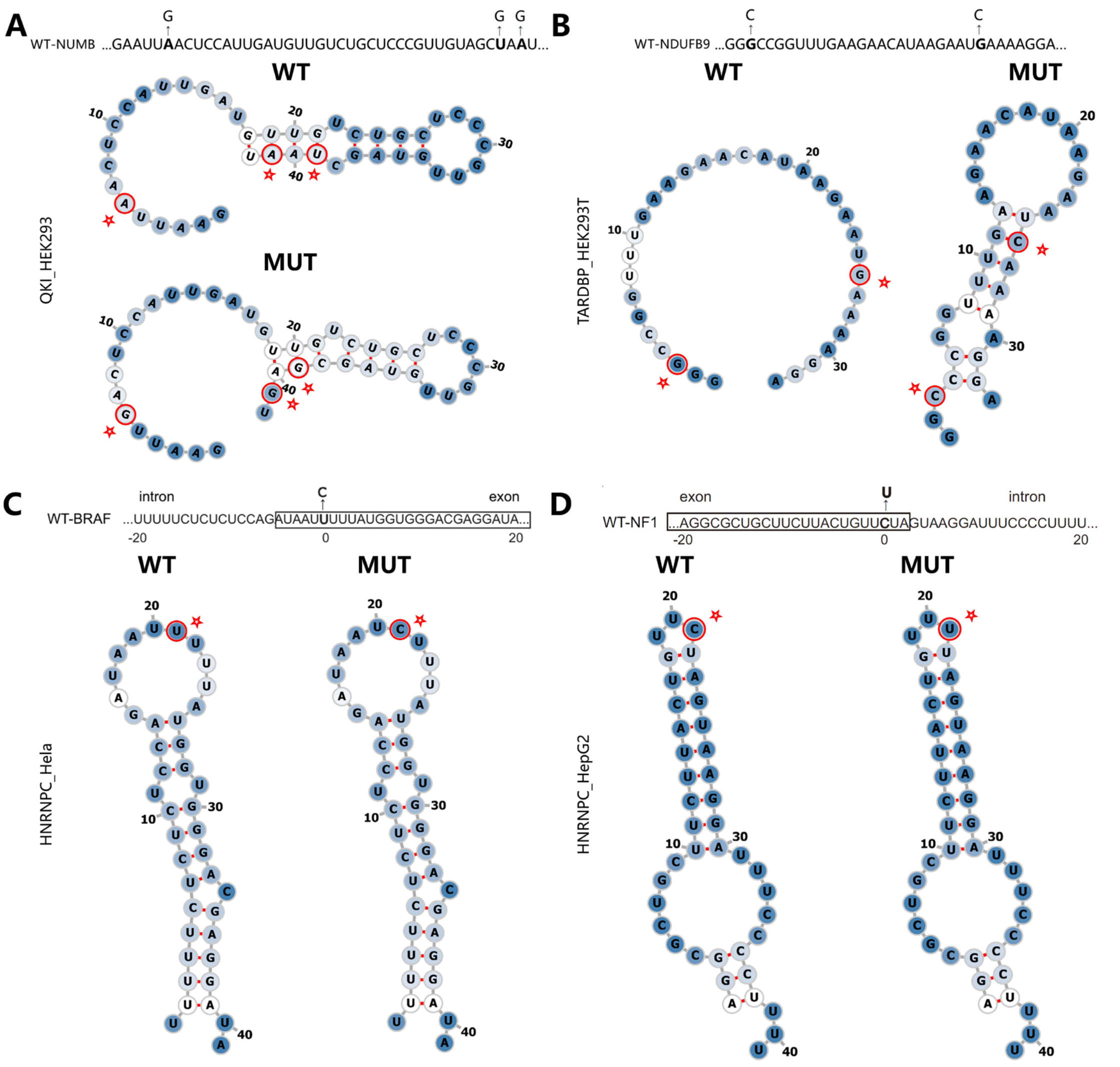

Figure S17. Impact of sequence variants on RNA secondary structure in high-attention binding regions. (A and B) Comparison of wild-type (left/top) and mutant (right/bottom) RNA secondary structures for a region with significant structural changes. The mutation leads to noticeable disruptions in key functional regions, potentially affecting RNA-RBP binding. (C and D) Comparison of wild-type (left) and mutant (right) RNA secondary structures for a region with minimal structural changes. The mutation has little impact on the RNA structure due to negative selective pressure.

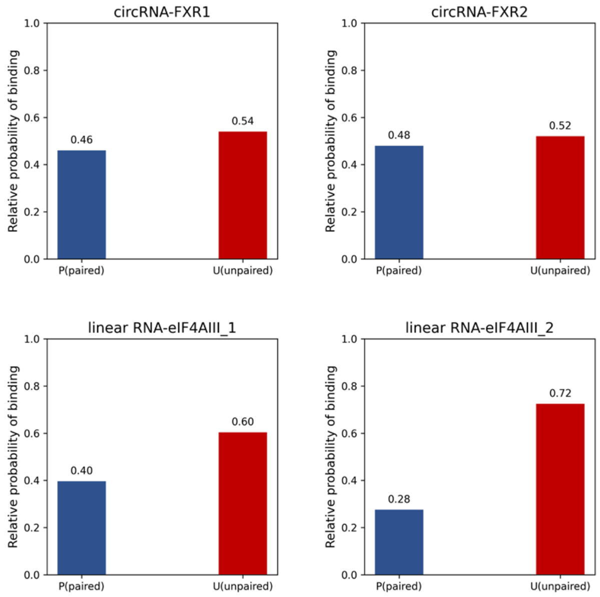

Figure S18. Structural preferences of circRNA binding with FXR family members (FXR1 and FXR2) and linear RNA binding with eIF4AIII_1 and eIF4AIII_2.

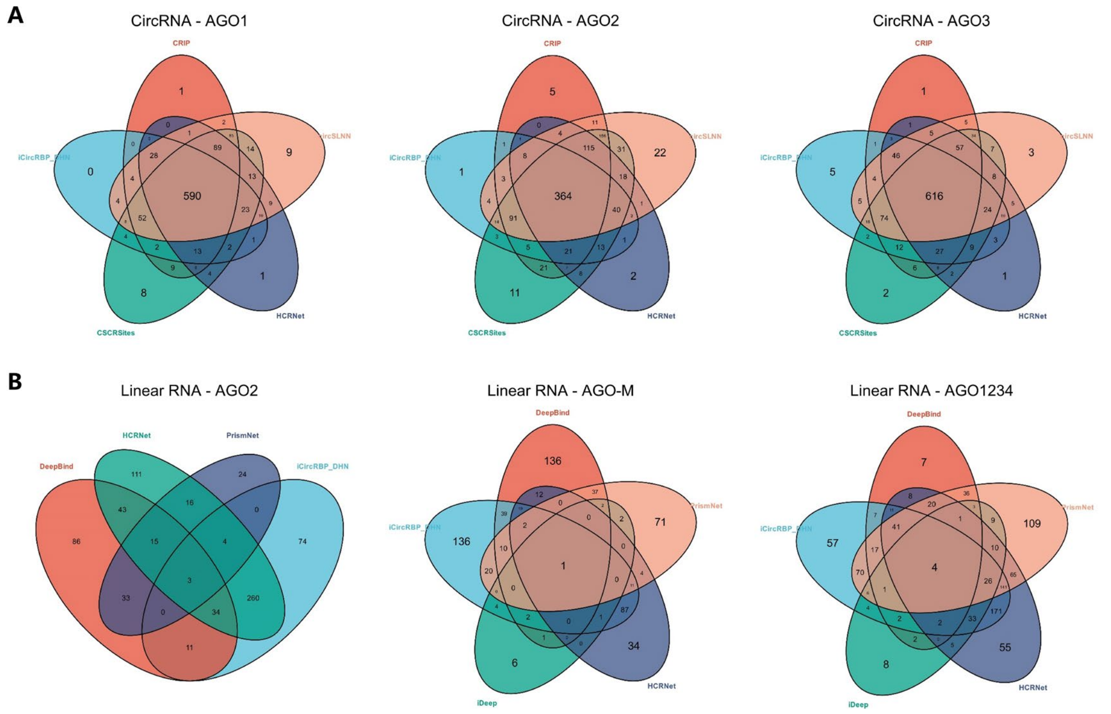

Figure S19. (A) Venn diagrams comparing the results of different methods for circRNA and AGO family interactions. (B) Venn diagrams comparing the results of different methods for linear RNA and AGO family interactions.

References

[1] Timothy Bailey and Charles Elkan. Bailey, t.l. and elkan, c. fitting a mixturemmodel by expectation maximization to discover motifs in biopolymers. proc. int. conf. intell. syst. mol. biol. 2, 28-36. Proceedings of the Second International Conference on Intelligent Systems for Molecular Biology, 2:28–36, 02 1994

[2] Lei Sun, Kui Xu, Wenze Huang, Yucheng T Yang, Pan Li, Lei Tang, Tuanlin Xiong, and Qiangfeng Cliff Zhang. Predicting dynamic cellular protein–rna interactions by deep learning using in vivo rna structures. Cell research, 31(5):495–516, 2021

[3] Haoran Zhu, Yuning Yang, Yunhe Wang, Fuzhou Wang, Yujian Huang, Yi Chang, Ka-chun Wong, and Xiangtao Li. Dynamic characterization and interpretation for protein-rna interactions across diverse cellular conditions using hdrnet. Nature Communications, 14(1):6824, 2023

[4] Wang, Z., Lei, X. and Wu, F.X., 2019. Identifying cancer-specific circRNA–RBP binding sites based on deep learning. Molecules, 24(22), p.4035.

[5] Zhang, K., Pan, X., Yang, Y. and Shen, H.B., 2019. CRIP: predicting circRNA–RBP-binding sites using a codon-based encoding and hybrid deep neural networks. Rna, 25(12), pp.1604-1615.

[6] Ju, Y., Yuan, L., Yang, Y. and Zhao, H., 2019. CircSLNN: identifying RBP-binding sites on circRNAs via sequence labeling neural networks. Frontiers in genetics, 10, p.1184.

[7] Yang, Y., Hou, Z., Ma, Z., Li, X. and Wong, K.C., 2021. iCircRBP-DHN: identification of circRNA-RBP interaction sites using deep hierarchical network. Briefings in Bioinformatics, 22(4), p.bbaa274.

[8] Yang, Y., Hou, Z., Wang, Y., Ma, H., Sun, P., Ma, Z., Wong, K.C. and Li, X., 2022. HCRNet: high-throughput circRNA-binding event identification from CLIP-seq data using deep temporal convolutional network. Briefings in Bioinformatics, 23(2), p.bbac027.

[9] Alipanahi, B., Delong, A., Weirauch, M.T. and Frey, B.J., 2015. Predicting the sequence specificities of DNA-and RNA-binding proteins by deep learning. Nature biotechnology, 33(8), pp.831-838.

[10] Pan, X. and Shen, H.B., 2017. RNA-protein binding motifs mining with a new hybrid deep learning based cross-domain knowledge integration approach. BMC bioinformatics, 18, pp.1-14.

[11] Chen, W., Lin, H. and Chou, K.C., 2015. Pseudo nucleotide composition or PseKNC: an effective formulation for analyzing genomic sequences. Molecular BioSystems, 11(10), pp.2620-2634.

[12] Gao, F. and Zhang, C.T., 2004. Comparison of various algorithms for recognizing short coding sequences of human genes. Bioinformatics, 20(5), pp.673-681.

[13] Zhang, R. and Zhang, C.T., 2014. A brief review: The z-curve theory and its application in genome analysis. Current genomics, 15(2), pp.78-94.

[14] Zhang, C.T. and Zhang, R., 1991. Analysis of distribution of bases in the coding sequences by a digrammatic technique. Nucleic Acids Research, 19(22), pp.6313-6317.

[15] Ghandi, M., Lee, D., Mohammad-Noori, M. and Beer, M.A., 2014. Enhanced regulatory sequence prediction using gapped k-mer features. PLoS computational biology, 10(7), p.e1003711.

[16] Bazzini, A.A., Johnstone, T.G., Christiano, R., Mackowiak, S.D., Obermayer, B., Fleming, E.S., Vejnar, C.E., Lee, M.T., Rajewsky, N., Walther, T.C. and Giraldez, A.J., 2014. Identification of small ORF s in vertebrates using ribosome footprinting and evolutionary conservation. The EMBO journal, 33(9), pp.981-993.

[17] Kang, Y.J., Yang, D.C., Kong, L., Hou, M., Meng, Y.Q., Wei, L. and Gao, G., 2017. CPC2: a fast and accurate coding potential calculator based on sequence intrinsic features. Nucleic acids research, 45(W1), pp.W12-W16.

[18] Yang, C., Yang, L., Zhou, M., **e, H., Zhang, C., Wang, M.D. and Zhu, H., 2018. LncADeep: an ab initio lncRNA identification and functional annotation tool based on deep learning. Bioinformatics, 34(22), pp.3825-3834.

[19] Liu, K. and Chen, W., 2020. iMRM: a platform for simultaneously identifying multiple kinds of RNA modifications. Bioinformatics, 36(11), pp.3336-3342.

[20] Tomas Mikolov, Ilya Sutskever, Kai Chen, Greg Corrado, and Jeffrey Dean. Distributed representations of words and phrases and their compositionality. Advances in neural information processing systems, 26, 2013.

[21] Quoc Le and Tomas Mikolov. Distributed representations of sentences and documents. In Proceedings of the 31st International Conference on International Conference on Machine Learning - Volume 32, ICML'14, page II–1188–II–1196. JMLR.org, 2014

[22] Piotr Bojanowski, Edouard Grave, Armand Joulin, and Tomas Mikolov. Enriching word vectors with subword information. Transactions of the Association for Computational Linguistics, 5:135–146, 2017.

[23] Jeffrey Pennington, Richard Socher, and Christopher Manning. Glove: Global vectors for word representation. In Proceedings of the 2014 Conference On Empirical Methods In Natural Language Processing (EMNLP), 2014.

[24] Ronny Lorenz, Stephan H Bernhart, Christian H¨oner zu Siederdissen, Hakim Tafer, Christoph Flamm, Peter F Stadler, and Ivo L Hofacker. Viennarna package 2.0. Algorithms for molecular biology, 6:1–14, 2011

[25] Bennasar, M., Hicks, Y. and Setchi, R., 2015. Feature selection using joint mutual information maximisation. Expert Systems with Applications, 42(22), pp.8520-8532.

[26] Viola, P. and Wells III, W.M., 1997. Alignment by maximization of mutual information. International journal of computer vision, 24(2), pp.137-154.

[27] Lin, D. and Tang, X., 2006. Conditional infomax learning: An integrated framework for feature extraction and fusion. In Computer Vision–ECCV 2006: 9th European Conference on Computer Vision, Graz, Austria, May 7-13, 2006. Proceedings, Part I 9 (pp. 68-82). Springer Berlin Heidelberg.

[28] Wang, G. and Lochovsky, F.H., 2004, November. Feature selection with conditional mutual information maximin in text categorization. In Proceedings of

the thirteenth ACM international conference on Information and knowledge management (pp. 342-349).

[29] Meyer, P.E., Schretter, C. and Bontempi, G., 2008. Information-theoretic feature selection in microarray data using variable complementarity. IEEE Journal of Selected Topics in Signal Processing, 2(3), pp.261-274.

[30] Jakulin, A., 2005. Machine learning based on attribute interactions (Doctoral dissertation, Univerza v Ljubljani).

[31] Battiti, R., 1994. Using mutual information for selecting features in supervised neural net learning. IEEE Transactions on neural networks, 5(4), pp.537-550.

[32] Peng, H., Long, F. and Ding, C., 2005. Feature selection based on mutual information criteria of max-dependency, max-relevance, and min-redundancy. IEEE Transactions on pattern analysis and machine intelligence, 27(8), pp.1226-1238.

[33] Robnik-Šikonja, M. and Kononenko, I., 2003. Theoretical and empirical analysis of ReliefF and RReliefF. Machine learning, 53, pp.23-69.

[34] Jia, Y., Nie, F. and Zhang, C., 2009. Trace ratio problem revisited. IEEE Transactions on Neural Networks, 20(4), pp.729-735.

[35] Gu, Q., Li, Z. and Han, J., 2012. Generalized fisher score for feature selection. arxiv preprint arxiv:1202.3725.

[36] Hall, M. A. (1999). Correlation-based feature selection for machine learning. Ph.D. Thesis, The University of Waikato.

[37] Budak, H. and Taşabat, S.E., 2016. A modified t-score for feature selection. Anadolu University Journal of Science and Technology A-Applied Sciences and Engineering, 17(5), pp.845-852.

[38] Polat, K. and Güneş, S., 2009. A new feature selection method on classification of medical datasets: Kernel F-score feature selection. Expert Systems with Applications, 36(7), pp.10367-10373.

[39] Franke, T.M., Ho, T. and Christie, C.A., 2012. The chi-square test: Often used and more often misinterpreted. American journal of evaluation, 33(3), pp.448-458.

[40] Gastwirth, J.L., 1972. The estimation of the Lorenz curve and Gini index. The review of economics and statistics, pp.306-316.

[41] Liu, J., Ji, S. and Ye, J., 2009. Multi-task feature learning via efficient ℓ2, 1-norm minimization. In Proceedings of the 25th Conference on Uncertainty in Artificial Intelligence, UAI 2009 (pp. 339-348). AUAI Press.

[42] Liguo Wang, Hyun Jung Park, Surendra Dasari, Shengqin Wang, Jean-Pierre Kocher, and Wei Li. CPAT: Coding-Potential Assessment Tool using an alignment-free logistic regression model. Nucleic Acids Research, 41(6):e74–e74, 01 2013. ISSN 0305-1048.

[43] Thomas Cover and Peter Hart. Nearest neighbor pattern classification. IEEE transactions on information theory, 13(1):21–27, 1967.

[44] J. Ross Quinlan. Induction of decision trees. Machine learning, 1:81–106, 1986.

[45] Irina Rish et al. An empirical study of the naive bayes classifier. In IJCAI 2001 workshop on empirical methods in artificial intelligence, volume 3, pages 41–46. Citeseer, 2001.

[46] Leo Breiman. Bagging predictors. Machine learning, 24:123–140, 1996.

[47] Rulan Wang, Chia-Ru Chung, Hsien-Da Huang, and Tzong-Yi Lee. Identification of species-specific RNA N6-methyladinosine modification sites from RNA sequences. Briefings in Bioinformatics, 24(2):bbac573, 01 2023. ISSN 1477-4054. doi: 10.1093/bib/bbac573.

[48] Yoav Freund and Robert E Schapire. A decision-theoretic generalization of on-line learning and an application to boosting. Journal of computer and system sciences, 55(1):119–139, 1997.

[49] Jerome H Friedman. Greedy function approximation: a gradient boosting machine. Annals of statistics, pages 1189–1232, 2001.

[50] Xiang HF Zhang, Katherine A Heller, Ilana Hefter, Christina S Leslie, and Lawrence A Chasin. Sequence information for the splicing of human pre-mrna identified by support vector machine classification. Genome Research, 13(12):2637–2650, 2003.

[51] Ronald A Fisher. The use of multiple measurements in taxonomic problems. Annals of eugenics, 7(2):179–188, 1936.

[52] Pierre Geurts, Damien Ernst, and Louis Wehenkel. Extremely randomized trees. Machine learning, 63:3–42, 2006.

[53] Xiaoyong Pan and Hong-Bin Shen. Predicting rna–protein binding sites and motifs through combining local and global deep convolutional neural networks. Bioinformatics, 34(20):3427–3436, 2018.

[54] Ilan Ben-Bassat, Benny Chor, and Yaron Orenstein. A deep neural network approach for learning intrinsic protein-RNA binding preferences. Bioinformatics, 34(17):i638–i646, 09 2018. ISSN1367-4803.

[55] Graves, A. and Graves, A., 2012. Long short-term memory. Supervised sequence labelling with recurrent neural networks, pp.37-45.

[56] Chung, J., Gulcehre, C., Cho, K. and Bengio, Y., 2014. Empirical evaluation of gated recurrent neural networks on sequence modeling. arxiv preprint arxiv:1412.3555.

[57] Bin Yu, Xue Wang, Yaqun Zhang, Hongli Gao, Yifei Wang, Yushuang Liu, and Xin Gao. Rpi-mdlstack: Predicting rna–protein interactions through deep learning with stacking strategy and lasso. Applied Soft Computing, 120:108676, 2022.

[58] Mengting Niu, Quan Zou, and Chen Lin. Crbpdl: Identification of circrna-rbp interaction sites using an ensemble neural network approach. PLoS computational biology, 18(1):e1009798, 2022.

[59] Lundberg, S.M. and Lee, S.I., 2017. A unified approach to interpreting model predictions. Advances in neural information processing systems, 30.

[60] Waskom, M.L., 2021. Seaborn: statistical data visualization. Journal of Open Source Software, 6(60), p.3021.

[61] Gruber, A.R., Lorenz, R., Bernhart, S.H., Neuböck, R. and Hofacker, I.L., 2008. The vienna RNA websuite. Nucleic acids research, 36(suppl_2), pp.W70-W74.

[62] Dudekula, D.B., Panda, A.C., Grammatikakis, I., De, S., Abdelmohsen, K. and Gorospe, M., 2016. CircInteractome: a web tool for exploring circular RNAs and

their interacting proteins and microRNAs. RNA biology, 13(1), pp.34-42.

[63] Stražar, M., Žitnik, M., Zupan, B., Ule, J. and Curk, T., 2016. Orthogonal matrix factorization enables integrative analysis of multiple RNA binding proteins. Bioinformatics, 32(10), pp.1527-1535.

[64] Zhu, Y., Xu, G., Yang, Y.T., Xu, Z., Chen, X., Shi, B., Xie, D., Lu, Z.J. and Wang, P., 2019. POSTAR2: deciphering the post-transcriptional regulatory logics. Nucleic acids research, 47(D1), pp.D203-D211.

[65] Van Nostrand, E.L., Freese, P., Pratt, G.A., Wang, X., Wei, X., Xiao, R., Blue, S.M., Chen, J.Y., Cody, N.A., Dominguez, D. and Olson, S., 2020. A large-scale binding and functional map of human RNA-binding proteins. Nature, 583(7818), pp.711-719.
